# The *Caenorhabditis elegans* microbiome impacts microsporidia infection through nutrient limitation and spore inactivation

**DOI:** 10.1038/s41467-026-77106-x

**Published:** 2026-08-26

**Authors:** Hala Tamim El Jarkass, Stefanie Castelblanco, Winnie Zhao, Manpreet Kaur, Yin Chen Wan, Abigail E. Ellis, Ryan D. Sheldon, Barbara Pees, Katja Dierking, Evan C. Lien, Nick O. Burton, Gerard D. Wright, Aaron W. Reinke

**Affiliations:** 1 Department of Molecular Genetics, University of Toronto, Toronto, ON Canada; 2 Michael G. DeGroote Institute for Infectious Disease Research & David Braley Centre for Antibiotic Discovery. McMaster University, Hamilton, ON Canada; 3 Mass Spectrometry Core, Van Andel Research Institute, Grand Rapids, MI USA; 4 Department of Evolutionary Ecology and Genetics, Zoological Institute, Kiel University, Kiel, Germany; 5 Department of Metabolism and Nutritional Programming, Van Andel Research Institute, Grand Rapids, MI USA

**Keywords:** Parasitology, Microbial communities

## Abstract

Microsporidia are fungal parasites that can only reproduce inside the cells of a host, and they infect most animal groups, including humans and agriculturally important species. Animals also harbor a microbiome, which can shape resistance to infection, but the mechanisms by which bacteria influence microsporidia infection are poorly understood. To address this, we tested how bacteria associated with the nematode *Caenorhabditis elegans* affect infection by its natural microsporidian parasite, *Nematocida parisii*. Here we show that nematodes grown on *Chryseobacterium scophthalmum* or *Sphingobacterium multivorum* exhibit increased initial parasite invasion but reduced subsequent growth. Broad profiling of host lipids reveals that these bacteria disrupt levels of unsaturated fatty acids, and adding back one such fatty acid, linoleic acid, restores parasite growth in animals raised on *S. multivorum*. We also found that *Pseudomonas lurida* and *Pseudomonas mendocina* secrete molecules that inactivate *N. parisii* spores, with *P. lurida* activity depending on the antimicrobial lipopeptide massetolide. We tested 53 additional *Pseudomonas* strains, 64% of which significantly reduced *N. parisii* infection. Our results indicate that bacterial interference with microsporidia is widespread, acting both by reshaping host metabolism and by directly inactivating parasite spores.

## Abstract (web summary)

Here, the authors show that gut bacteria can protect nematodes from microsporidia via altering host fatty acids the parasites need to grow, and secreting molecules that inactivate parasite spores, revealing widespread bacterial impact on infection.

## Introduction

Organisms are tightly connected with the microbial entities that exist both within and around them. Some members of the microbiome act as pathogens, causing disease, whereas others play important roles in host health^1^. Beneficial members of the microbiome can protect against pathogens through limiting host resources, secretion of antimicrobial compounds, or stimulating host immunity^2–4^. These beneficial microbes can both protect the host and influence how pathogens evolve^5,6^. Understanding these mechanisms of host protection can lead to harnessing bacteria to prevent infectious disease^7^.

The model organism *Caenorhabditis elegans* has become a powerful system to mechanistically study complex interactions between a host, beneficial microbes, and pathogens^8^. This invertebrate is commonly isolated from rotting plant matter and is associated with over 250 distinct bacterial isolates consisting of mostly Gammaproteobacteria and Bacteroidetes^9,10^. These associated bacteria predominantly act as a food source for *C. elegans*, though 20% of these bacterial isolates can be pathogenic^11^. A community effort combining sampling efforts from distinct geographical locations resulted in the generation of a common set of bacteria that frequently co-occur with this nematode in the wild. This resulting bacterial collection, known as CeMbio, is composed of 12 different species that represent ~60% of the known diversity of *C. elegans*-associated bacteria^12^. Members of this collection can defend against bacterial and viral pathogens. Examples include *Pseudomonas mendocina* MSPm1 protecting against *Pseudomonas aeruginosa* through activation of the p38 Mitogen-activated protein kinase pathway, *Pseudomonas lurida* MYb11 producing the antimicrobial compound massetolide E to inhibit *Bacillus thuringiensis* infection, and *Chryseobacterium scophthalmum* JUb44 providing resistance to Orsay virus infection that is independent of known antiviral pathways^13–17^.

Microsporidia are ubiquitous obligate intracellular fungal parasites that infect most types of animals^18–20^. These parasites can cause disease in humans as well as agriculturally important animals such as fish, shrimp, honey bees, and silkworms^21–23^. The presence of microsporidia in the microbiome has been extensively studied, with reported prevalence rates of over 60% in animal populations^24–27^. Possessing the smallest known eukaryotic genome sizes, these parasites have lost many metabolic enzymes and are extremely host dependent, utilizing a variety of transporters to uptake nutrients, including ATP^28–33^. The host microbial diet can impact microsporidia, such as bacterially-produced vitamin B12, providing tolerance to infection^34^. Bacteria can also produce molecules that inhibit microsporidia and bacteria engineered to produce double-stranded RNA protect against microsporidia infection^35–38^.

Microsporidia are common parasites of *C. elegans* in nature, and the most frequently observed species is *Nematocida parisii*^33,39–44^. Infection begins when *C. elegans* ingest dormant *N. parisii* spores. These spores contain a unique invasion apparatus called the polar tube. When spores come into contact with a host cell, the polar tube rapidly emerges and is thought to inject the intracellular components of the spore, the sporoplasm, inside of the target host cell, which can occur as early as two minutes after being exposed to spores^41,45^. In approximately 12–18 h, meronts will begin to form, resulting in the formation of mature dormant spores as early as 48 h post-infection^46^. *N. parisii* infections in the wild happen within the context of the large collection of bacterial species that are associated with *C. elegans*, but how the bacterial diet influences *N. parisii* growth or if these bacteria produce molecules that inhibit microsporidia infection is unknown.

To understand how the *C. elegans* microbiome impacts *N. parisii* infection, we utilized the CeMbio collection of bacteria. We found that *Sphingobacterium multivorum* BIGb0170 and *C. scophthalmum* JUb44 delay *N. parisii* growth through nutrient limitation. Growth on *S. multivorum* BIGb0170 causes disruption of unsaturated fatty acids within *C. elegans*, and that supplementation of linoleic acid can restore *N. parisii* growth. Our results indicate that linoleic acid is a limiting factor for *N. parisii* to complete its lifecycle as an infected *C. elegans* mutant that is unable to make this polyunsaturated (PUFA) fatty acid, experiences delayed differentiation into spores. Additionally, we show that *P. lurida* MYb11 and *P. mendocina* MSPm1 secrete distinct compounds that inhibit *N. parisii* spores. We tested an additional 53 *C. elegans*-associated *Pseudomonas* isolates, demonstrating that 64% of them can inhibit *N. parisii*. We also show that a mixture of eight *Pseudomonas* species reduced *N. parisii* infection by 80% and that this protection lasts at least 10 generations. Altogether, our study highlights how the *C. elegans* microbiome can impact *N. parisii* infection by disrupting unsaturated fatty acid availability and that many *Pseudomonas* species secrete molecules that inactivate *N. parisii* spores.

## Results

### Members of the *C. elegans* microbiome increase *C. elegans* feeding and reduce *N. parisii* growth through nutritional deficiency

**Fig. 1:**
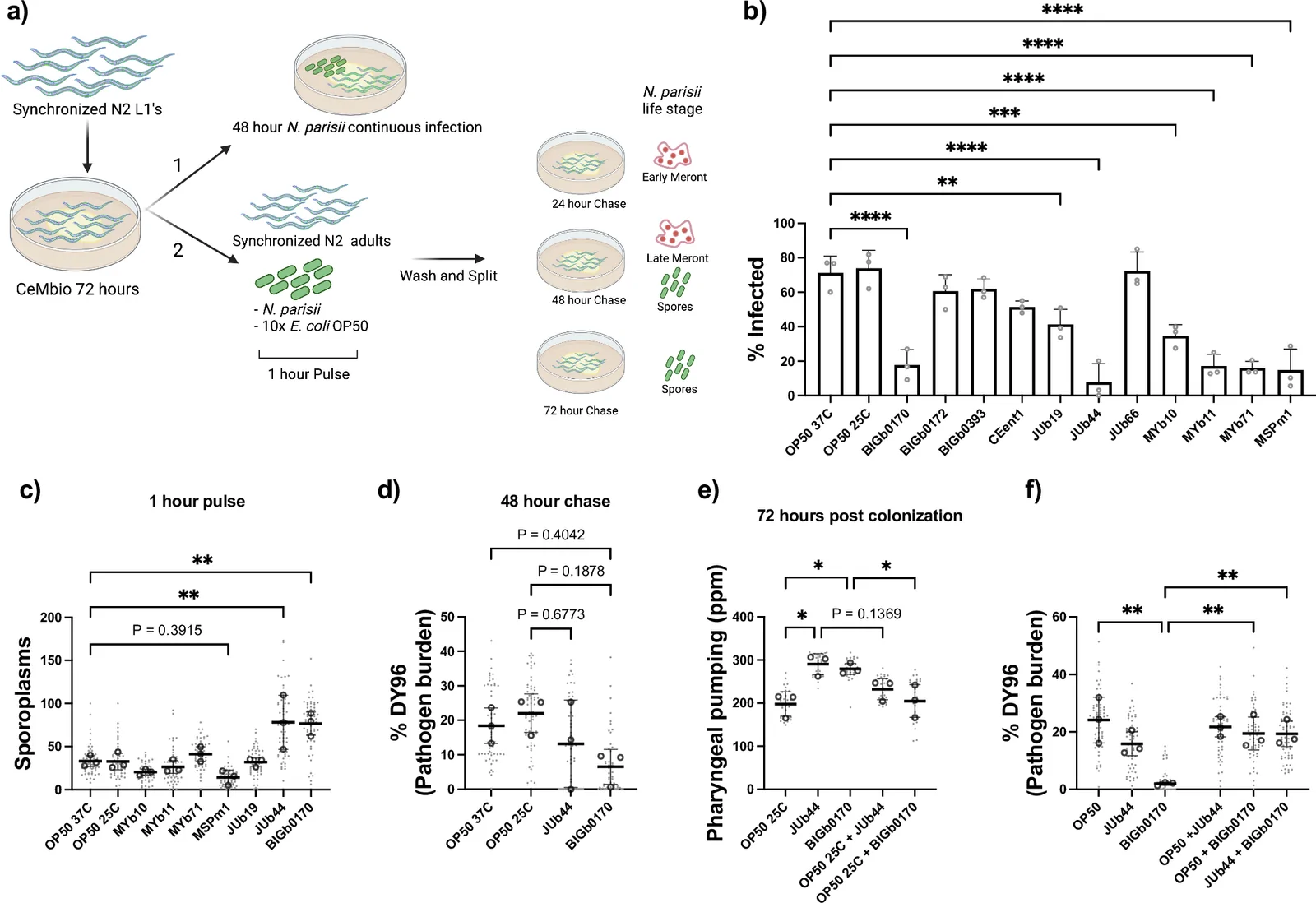
Growth on *C. scophthalmum* JUb44 and *S. multivorum* BIGb0170 results in decreased *N. parisii* infection burden. **a** Schematic depicting the workflow involved in the colonization of nematodes with CeMbio strains and the experimental pipeline. Synchronized L1 animals were grown on individual bacterial strains for 72 h. Synchronized adult animals were (1) continuously infected with *N. parisii* on CeMbio lawns or (2) exposed to a 1-h pulse, followed by a 24- or 48-h chase. Sporoplasms and meronts can be detected at earlier time points using FISH and spores can be detected at later time points using DY96. **b** The fraction of worms displaying spores 48 hpi. **c**, **d** 72-h old adults were pulse infected with *N. parisii* and *E. coli* OP50 for one hour and fixed at 1 hpi (**c**) or 48 hpi (**d**) and stained with a FISH probe and DY96 and the amount of pathogen load was quantified. **e** Bacterial strains were used in isolation or in a 1:1 ratio with *E. coli* OP50 and nematodes were grown for 72 h. The number of pharyngeal pumps per minute was quantified. **f** 72-h old adults grown on the indicated bacteria were pulse infected for one hour and spores quantified at 48 hpi. Data are from *n* = 3 biological replicates of 100 (**b**), 15–22 (**c**, **d**, and **f**) or 10 worms each (**e**). Mean ± SD represented by horizontal bars. *P*-values determined via one-way ANOVA with Dunnett’s multiple comparisons test (**b**, **c**) or with Šidák’s multiple comparisons test (**d**–**f**). All test conditions were compared to *E. coli* OP50 37 °C (**b**, **c**) or to both *E. coli* OP50 37 °C and 25 °C (**d**). Comparisons were also made between mixed lawns and their individual bacteria components (**e**, **f**). Significance defined as * *p* < 0.05, ** *p* < 0.01, *** *p* < 0.001, **** *p* < 0.0001 and all *p*-values are included in the source data file. Schematic created in BioRender. Reinke, A. (2026) https://BioRender.com/lhybxpd.

To investigate how growth on different bacterial isolates impacts *N. parisii* infection, we exposed wild-type N2 L1 stage *C. elegans* to individual lawns of CeMbio bacterial strains for 72 h. All CeMbio strains were cultured at 25 °C and as a control we included the typical lab diet of *Escherichia coli* OP50 grown at the routine temperature of 37 °C or at 25 °C (see “Methods”). These young adult animals were then exposed to 48 h of continuous infection with *N. parisii* spores, fixed, and stained with direct yellow 96 (DY96), a dye that binds to the chitin-containing spores (Fig. 1a). *N. parisii* growth was hindered when nematodes were grown on 7 of the 11 CeMbio strains, as determined by a reduction in the fraction of worms producing spores (Fig. 1b).

The most critical step in microsporidia infection is the invasion of host cells by microsporidian sporoplasms. To determine if decreased pathogen burdens are a result of impaired invasion when grown on CeMbio strains, we exposed *C. elegans* adults grown on bacterial strains for 72 h to *N. parisii* spores. Altered invasion may be a result of direct effects of bacteria in the lumen on microsporidia spores or alterations in host physiology by bacteria. After 1 h of exposure, animals were fixed and stained with an *N. parisii*-specific 18S rRNA Fluorescence in situ Hybridization (FISH) probe. Nematodes grown on *S. multivorum* BIGb0170 or *C. scophthalmum* JUb44 displayed a significant increase in the number of sporoplasms (Fig. 1c). Although animals grown on *S. multivorum* BIGb0170 and *C. scophthalmum* JUb44 were initially more infected, they surprisingly produced fewer spores, so we chose to investigate the basis of these phenotypes in subsequent experiments (Fig. 1b).

To understand how growth on *S. multivorum* BIGb0170 or *C. scophthalmum* JUb44 results in decreased *N. parisii* proliferation, we performed a pulse-chase assay, monitoring how *N. parisii* infection progresses with time (Fig. 1a). Seventy-two-hour old *C. elegans* adults were exposed to a one-hour pulse infection with *N. parisii* and un-ingested spores were washed away prior to placing the animals back on the corresponding lawns of bacteria. Animals were then washed and fixed at various time points and stained using a FISH probe and DY96. At either 24 h post-infection (hpi) or 48 hpi, there was no significant reduction of meronts of worms grown on either *S. multivorum* BIGb0170 or *C. scophthalmum* JUb44 (Supplementary Fig. 1a, b). At 48 hpi a small, but non-significant reduction in spores was observed when worms were grown on *S. multivorum* BIGb0170 (Fig. 1d). L1 stage *C. elegans* grown on *S. multivorum* BIGb0170 or *C. scophthalmum* JUb44 for only 24 h prior to infection did not display increased invasion or decreased pathogen proliferation (Supplementary Fig. 1c–f).

*S. multivorum* BIGb0170 and *C. scophthalmum* JUb44 were previously reported to result in developmental delay of *C. elegans*, suggesting they may not be optimal food sources^12^. Poor quality diets can influence nematode feeding behaviors, including pharyngeal pumping^47–49^. Nematodes grown on *S. multivorum* BIGb0170 or *C. scophthalmum* JUb44 for 72 h displayed significantly increased levels of pharyngeal pumping (Fig. 1e). Of the seven bacteria that caused a reduction in the fraction of worms producing spores in Fig. 1b, only *S. multivorum* BIGb0170 and *C. scophthalmum* JUb44 increased the rate of pharyngeal pumping (Supplementary Fig. 1g). To assess if this behavioural change was a result of altered nutritional quality, we grew nematodes on lawns of either bacterial species mixed with *E. coli* OP50 in a 1:1 ratio, which resulted in significantly reduced pharyngeal pumping rates for nematodes grown on BIGb0170 (Fig. 1e). Animals grown on BIGb0170 alone displayed significantly reduced spore production (Fig. 1f). Animals grown on mixed lawns of either BIGb0170 mixed with OP50 or JUb44 resulted in wild-type levels of *N. parisii* spore production (Fig. 1f). Together, our results suggest that more active pharyngeal pumping results in increased *N. parisii* invasion and a nutritional component of the *E. coli* OP50 diet may be missing from *S. multivorum* BIGb0170 and *C. scophthalmum* JUb44.

### Linoleic acid is a limiting factor for *N. parisii* growth

**Fig. 2:**
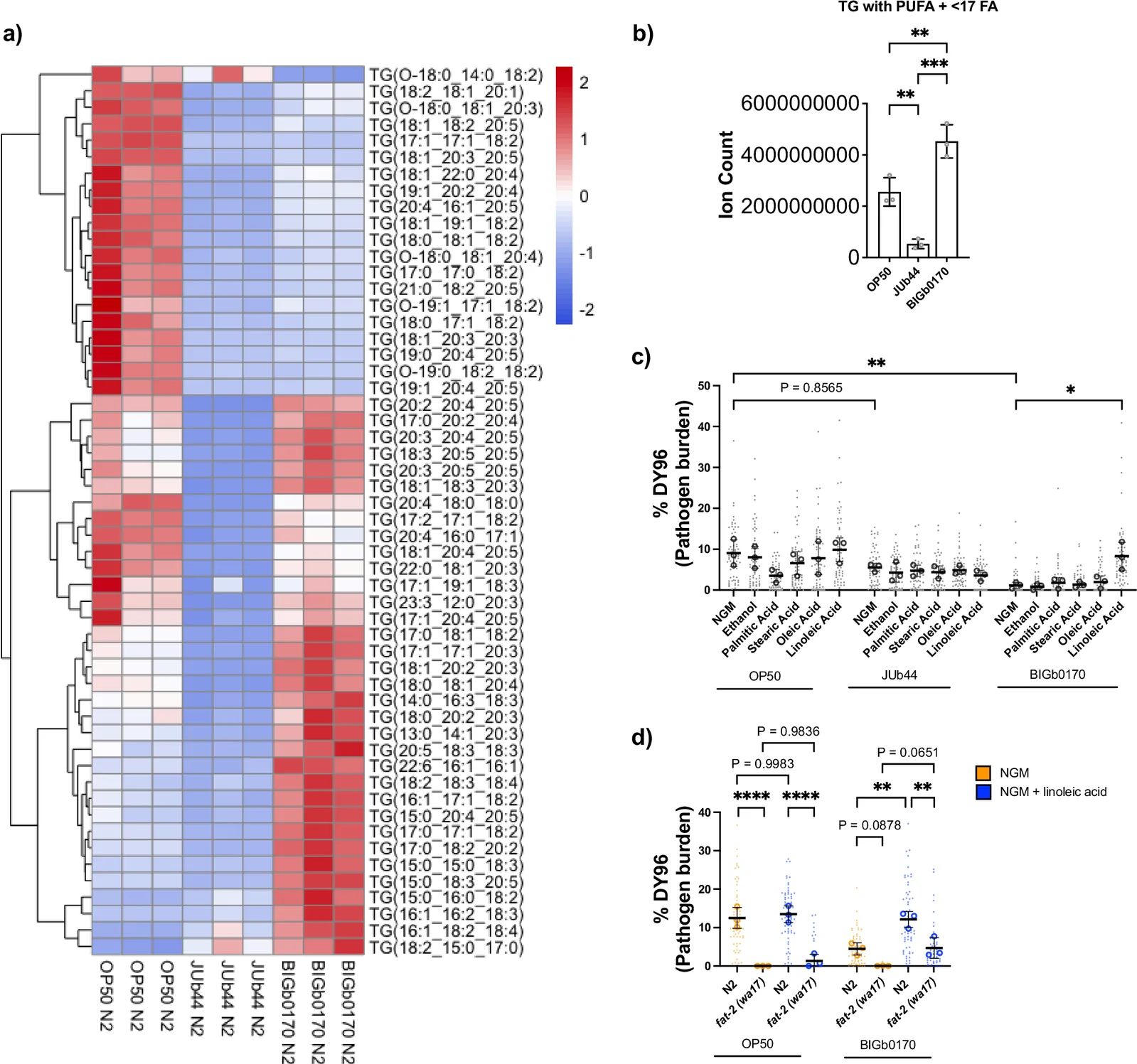
Linoleic acid supplementation rescues *N. parisii* growth on *S. multivorum* BIGb0170. **a** A heat map depicting the abundance of triglycerides (TG) containing a polyunsaturated fatty acid (PUFA) acyl chain in N2 animals grown on *E. coli* OP50, *C. scophthalmum* JUb44 or *S. multivorum* BIGb0170 from three independent replicates. Scale bar represents *Z*-score values. **b** Ion counts of triglycerides containing both a PUFA acyl chain and an acyl chain containing fewer than 17 carbons in N2 animals grown on the different bacterial diets. **c** Various mono- and polyunsaturated fatty acids were supplemented in NGM media when nematodes were colonized with bacterial species denoted on the *X*-axis and spores quantified. **d** N2 or *fat-2 (wa17)* animals were grown on plates with (blue) or without (orange) linoleic acid for 72 h. These animals were then infected for one hour with *N. parisii* and fixed at 48 hpi. The bacterial diet is denoted with a solid line below the *X*-axis. *P*-values from all comparisons are shown above the graph. Data are from *n* = 3 biological replicates (**a**–**d**), with 20 worms counted (**c**, **d**). Mean ± SD represented by horizontal bars. *P*-values determined via one-way ANOVA with Tukey’s multiple comparisons test (**b**, **c**) or with Šidák’s multiple comparisons test (**d**). Comparisons were made between all possible pairs (**b**, **c**). Significance defined as * *p* < 0.05, ** *p* < 0.01, *** *p* < 0.001, **** *p* < 0.0001 and all *p*-values are included in the source data file.

Because growth on *S. multivorum* BIGb0170 and *C. scophthalmum* JUb44 caused delayed development and altered feeding behavior, we hypothesized that they were altering the metabolism of the nematodes. To determine if growth on these two bacterial isolates altered the metabolites present in *C. elegans*, we performed untargeted metabolomics. We grew animals for 72 h on either *E. coli* OP50, *S. multivorum* BIGb0170, or *C. scophthalmum* JUb44. Analysis of the polar metabolic data did not reveal strong differences that occurred upon growth of worms on either of these CeMbio strains (Supplementary Data 1). In contrast, analysis of the lipid metabolic data revealed that animals grown on either *S. multivorum* BIGb0170 or *C. scophthalmum* JUb44 had altered levels of three PUFA lipid classes: triglycerides (TG), phosphatidylcholine (PC), and phosphatidylethanolamine (PE) relative to animals grown on *E. coli* OP50 (Fig. 2a, Supplementary Fig. 2a, b, and Supplementary Data 2). Animals grown on *C. scophthalmum* JUb44 had largely reduced TG levels, whereas animals grown on *S. multivorum* BIGb0170 contained classes of TGs that were either reduced or elevated compared to animals grown on *E. coli* OP50. Animals grown on *C. scophthalmum* JUb44 had significantly fewer TGs with acyl chains lengths less than 17, whereas those grown on *S. multivorum* BIGb0170 had significantly more (Fig. 2b).

As our metabolism data showed differences in triglyceride levels, we tested whether fatty acids are necessary for microsporidia growth on *S. multivorum* BIGb0170 and *C. scophthalmum* JUb44 by supplementing bacterial diets with various fatty acids. Supplementation of palmitic (C16:0), stearic (C18:0), oleic (C18:1), or linoleic (C18:2) acid revealed that only the PUFA linoleic acid was able to restore *N. parisii* growth in animals grown on *S. multivorum* BIGb0170 (Fig. 2c). We did not observe an effect of fatty acid supplementation on *N. parisii* growth on animals grown on *C. scophthalmum* JUb44 (Fig. 2c). The abundance of C18:2-containing TGs differed between animals grown on *C. scophthalmum* JUb44 and *S. multivorum* BIGb0170 whereas C18:2-containing PE and PCs were generally lower overall (Supplementary Fig. 2c–e).

To test the importance of linoleic acid on *N. parisii* proliferation, we used a mutant of *fat-2*, which encodes a △12 desaturase and plays an essential role in the conversion of oleic acid (C18:1) to linoleic acid (C18:2)^50^. Animals lacking FAT-2 contain depleted amounts of linoleic acid^51^. When *fat-2 (wa17*) animals were grown on *E. coli* OP50 or *S. multivorum* BIGb0170 for 72 h, we observed a complete block in *N. parisii* sporulation 48 h later (Fig. 2d). To confirm that this phenotype was due to a lack of linoleic acid, we performed the same experiment with supplementation of linoleic acid. *fat-2 (wa17)* animals displayed improved, but non-significant, *N. parisii* sporulation on both bacterial sources when linoleic acid was supplemented. Linoleic acid supplementation also improved *fat-2 (wa17)* fitness as seen by an increase in the number of embryos after 72 h of growth on both *E. coli* OP50 and *S. multivorum* BIGb0170 (Supplementary Fig. 3a). To determine if a lack of linoleic acid impacted *N. parisii* growth over time, we performed pulse-chase experiments. At 48 hpi, sporulation was also greatly reduced in the *fat-2* mutant (Supplementary Fig. 3b). However, at 72 hpi, *fat-2 (wa17)* animals displayed higher levels of sporulation than observed at 48 h on either diet (Supplementary Fig. 3c). Together, these results suggest that linoleic acid is a limiting nutrient for efficient growth of *N. parisii* in *C. elegans*.

As animals grown on *S. multivorum* BIGb0170 resulted in increased sporoplasm invasion, we investigated if linoleic acid was responsible for this phenotype. We measured the number of sporoplasms in N2 and *fat-2 (wa17)* animals with and without linoleic acid and observed no differences (Supplementary Fig. 4a). Oleic acid has previously been reported to be important for feeding behavior in *C. elegans*^52^. To determine if supplementation with this lipid could rescue the increased invasion, we performed invasion experiments, observing that oleic acid, but not linoleic acid, reduced sporoplasm levels when worms were grown on *S. multivorum* BIGb0170, but not on *C. scophthalmum* JUb44 (Supplementary Fig. 4b). Together these data show that oleic acid can rescue the feeding behaviour of animals grown on *S. multivorum* BIGb0170 and provides further support that these two Bacteroidetes bacteria display different nutritional deficiencies.

### Growth on *P. lurida* MYb11 improves *C. elegans* fitness and reduces *N. parisii* proliferation

**Fig. 3:**
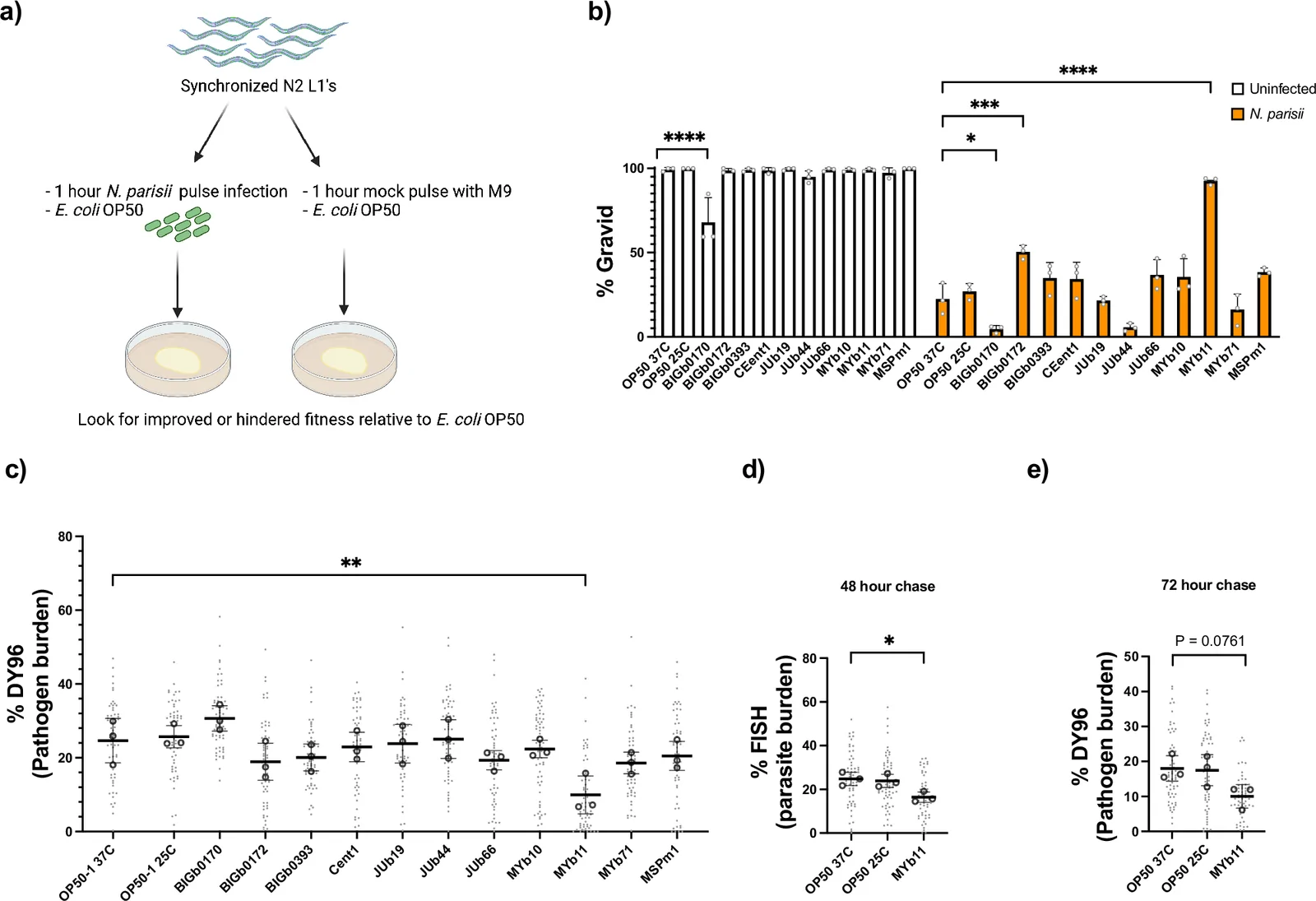
Growth on *P. lurida* MYb11 inhibits *N. parisii* proliferation. **a** Schematic depicting the experimental pipeline. Synchronized N2 L1 animals were either infected with *N. parisii* or mock-treated for 1 h on *E. coli* OP50-1 prior to washing and plating onto various CeMbio strains. Seventy-two hours later, population fitness (**b**) and pathogen load (**c**) were quantified. **d**, **e** Synchronized N2 L1s were pulse infected with *N. parisii* for 1 h on *E. coli* OP50-1 prior to washing and splitting the populations onto *P. lurida* MYb11 seeded plates. Animals were fixed and then stained with an *N. parisii* 18S rRNA FISH probe at 48 hpi (**d**) or DY96 at 72 hpi (**e**). Data are from *n* = 3 biological replicates of at least 100 (**b**) or 15–20 (**c**–**e**) worms each. Mean ± SD represented by horizontal bars. *P*-values determined via one-way ANOVA with Dunnett’s multiple comparisons test, with a single pooled variance. All test conditions were compared to the corresponding *E. coli* OP50 37 °C. Significance defined as * *p* < 0.05, ** *p* < 0.01, *** *p* < 0.001, **** *p* < 0.0001 and all *p*-values are included in the source data file. Schematic created in BioRender. Reinke, A. (2026) https://BioRender.com/5x9sv1h.

In addition to providing nutrients to hosts, bacteria also can produce compounds that can protect against infection. Like many microsporidia, *N. parisii* infection reduces embryo formation and overall progeny produced^34,53^. To determine how *C. elegans* is impacted during *N. parisii* infection while feeding on these diets, we pulse-infected animals for one hour with *N. parisii* and *E. coli* OP50 and placed infected animals onto individual CeMbio lawns (Fig. 3a). This approach allows us to rule out any effects that the CeMbio diet may have on *N. parisii* invasion. Growth on most of the CeMbio strains in the absence of infection resulted in the ability of almost all animals to become gravid (containing embryos) 72 h post L1, except for *S. multivorum* BIGb0170, which is consistent with previous findings (Fig. 3b)^11,12^. However, when exposed to *N. parisii*, populations of nematodes grown on *P. lurida* MYb11 displayed ~fourfold higher gravidity than those grown on *E. coli* OP50, with population gravidity near that of uninfected animals. We also observed that *Comamonas piscis* BIGb0172 displayed a moderate increase in fitness and those grown on *S. multivorum* BIGb0170 displayed reduced fitness (Fig. 3b).

We also determined the impact of these diets on *N. parisii* growth. Culturing of nematodes on *P. lurida* MYb11 resulted in ~twofold decrease in pathogen load (Fig. 3c). To monitor infection progression on *P. lurida* MYb11, we pulse infected N2 L1 animals for one hour, with 48- (late meront) and 72-h (spores) chase timepoints. Infected N2 animals grown on *P. lurida* MYb11 consistently displayed reduced pathogen loads throughout development (Fig. 3d, e). Lastly, to assess if the contributions of the *P. lurida* MYb11 diet were only advantageous at specific timepoints during infection, animals were grown on either *E. coli* OP50 or *P. lurida* MYb11 for varying amounts of time. We observed a significant increase in the percentage of gravid worms when initially grown on MYb11 for any amount of time and significantly decreased pathogen loads only when worms were grown on MYb11 the entire time (Supplementary Fig. 5a, b).

Previously, it was shown that bacteria producing vitamin B12 induce developmental acceleration in *C. elegans* and several of the CeMbio isolates, including *P. lurida* MYb11, are predicted to contain pathways for vitamin B12 production^12,54,55^. We have previously shown that parents exposed to vitamin B12 produce progeny that have enhanced tolerance to *N. parisii*^34^. To determine if vitamin B12 is produced in *P. lurida* MYb11, we monitored the expression levels of acyl-CoA dehydrogenase *acdh-1*, a dietary sensor of vitamin B12 deficiency^55^. When *Pacdh-1::gfp* L1 animals were grown on lawns of *Comamonas aquatica* DA1877, a bacterial species known to produce vitamin B12, or *P. lurida* MYb11, we observed repressed GFP expression (Supplementary Fig. 6a). Our results are consistent with what has been recently shown for this reporter strain grown on *P. lurida* MYb11^56^. To determine if the vitamin B12 produced by *P. lurida* MYb11 influenced the enhanced fitness we observed during *N. parisii* infection, we tested mutants in two pathways that utilize vitamin B12: the methionine/SAM cycle *(metr-1)* and the propionyl-CoA breakdown pathway *(mmcm-1)*. Although infected N2 and *mmcm-1(ok1637)* animals display increased fitness when grown on *C. aquatica* DA1877 and *P. lurida* MYb11, only worms grown on *P. lurida* MYb11 display reduced pathogen loads. The *metr-1(ok521)* animals grown on *C. aquatica* DA1877 or *P. lurida* MYb11 no longer display increased fitness, but these mutant animals reared on lawns of *P. lurida* MYb11 still display reduced pathogen loads (Supplementary Fig. 6b, c). This supports a model where the enhanced fitness is mediated through production of vitamin B12, but the resistance to infection is not.

### Conditioned media from CeMbio isolates attenuate *N. parisii* spore infectivity

**Fig. 4:**
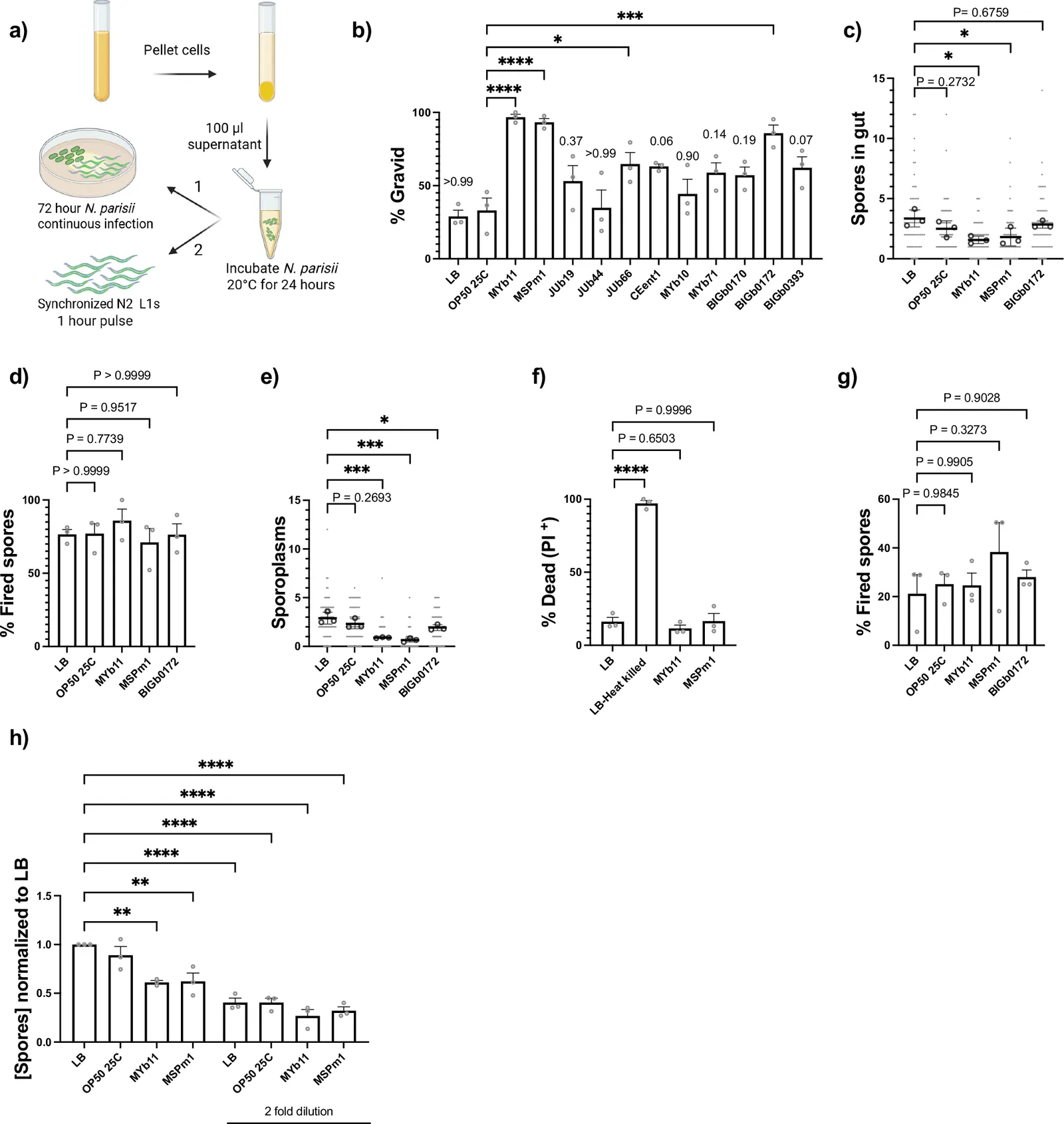
*P. lurida* MYb11 and *P. mendocina* MSPm1 secrete molecules which cause *N. parisii* spore destruction. **a** Schematic depicting the experiment. LB media as a control or supernatants from various CeMbio strains of interest were incubated with *N. parisii* spores for 24 h at 20 °C. Synchronized N2 L1 animals and *E. coli* OP50 were then added to the spore-supernatant mixtures and plated on a 6-cm unseeded NGM plate for 72 h (1) (**b**) or 1 h (2) (**c**–**e**). **b** Quantification of percentage of population that display gravidity at 72 hpi. Each test condition was compared to *E. coli* OP50, with values above bars indicating *p*-values. **c**–**e** Pre-incubated spores were used to infect N2 L1s for one hour. Pathogen invasion was assessed. The number of spores per animal (**c**), the percentage of fired spores (**d**) and the number of sporoplasms (**e**) are displayed. **f**–**h** *N. parisii* live and/or heat-killed spores (**f**) were pre-incubated in bacterial supernatants and treated with propidium iodide (**f**). The fraction of propidium iodide^+^ spores was quantified. **g** The fraction of fired spores post-supernatant incubation was quantified. **h** The concentration of spores 24 h post-incubation was quantified and normalized to spores incubated in LB. Data are from *n* = 3 biological replicates of 100 worms (**b**) or 20 worms each (**c**–**e**) and at least 50 spores each (**c**, **d** and **f**–**h**). Mean ± SD represented by horizontal bars. *P*-values determined via one-way ANOVA with Dunnett’s multiple comparisons test (**b**–**g**) or with Tukey’s multiple comparisons test (**h**), with a single pooled variance. All test conditions were compared to OP50 25 °C (**b**) or to LB control (**c**–**g**) or comparisons were made between all possible pairs (**h**). Significance defined as * *p* < 0.05, ** *p* < 0.01, *** p < 0.001, **** *p* < 0.0001 and all *p*-values are included in the source data file. Schematic created in BioRender. Reinke, A. (2026) https://BioRender.com/nj24j9m.

In addition to inhibiting microsporidia proliferation, it is possible that bacteria could make compounds that inhibit microsporidia spores. To investigate if the CeMbio species target *N. parisii*, we incubated spores in conditioned media from individual bacterial species and infected synchronized N2 L1 animals for 72 h (Fig. 4a). Animals infected with *N. parisii* spores incubated in the supernatants of *P. lurida* MYb11, *P. mendocina* MSPm1, *Lelliottia amnigena* JUb66, and *C. piscis* BIGb0172 had increased proportions of gravid adult animals (Fig. 4b). To assess if these supernatants may influence *N. parisii* infectivity, we performed a one-hour pulse infection with supernatant-treated spores and measured invasion phenotypes (Fig. 4a). Microsporidia invasion can be broken down into three main stages: spore ingestion, spore firing, and sporoplasm deposition. A perturbation in any of these three processes would greatly improve host fitness. The number of DY96-stained spores present in the nematode gut was significantly lower under *P. lurida* MYb11 and *P. mendocina* MSPm1 treatment conditions (Fig. 4c). Next, we quantified the fraction of fired spores, revealing no significant differences between the different treatment conditions (Fig. 4d). Lastly, we quantified the number of intracellular sporoplasms within the nematode intestinal cells. We saw a significant reduction in the number of sporoplasms under *P. lurida* MYb11, *P. mendocina* MSPm1, and *C. piscis* BIGb0172 treatment conditions (Fig. 4e), with the strongest effect being observed with the two *Pseudomonas* species. The activity of the conditioned media is likely acting directly on the spores, as *N. parisii* spores treated with media and then washed are still less infective, as seen by improved host fitness under these conditions (Supplementary Fig. 7).

Molecules could inactivate microsporidia spores by making them less viable, inducing germination, or by reducing spore numbers^57,58^. Propidium iodide (PI) is a dye that only stains non-viable cells. Spores treated with *P. lurida* MYb11 or *P. mendocina* MSPm1 supernatants did not display increased levels of PI staining compared to heat-killed spores, indicating that this was not the mechanism of action (Fig. 4f). We next tested if supernatant treatment induces premature spore firing in vitro. However, the fraction of fired spores was relatively similar under all treatment conditions, making this scenario unlikely (Fig. 4g). Given that *P. lurida* MYb11 and *P. mendocina* MSPm1 supernatants reduce the number of DY96-stained spores in the nematode gut, we tested the possibility of in vitro spore destruction. Incubation of *N. parisii* spores in either supernatant resulted in a decreased concentration of DY96-stained spores in vitro (Fig. 4h).

### Massetolide E and F produced by *P. lurida* MYb11 inhibit *N. parisii* spores

**Fig. 5:**
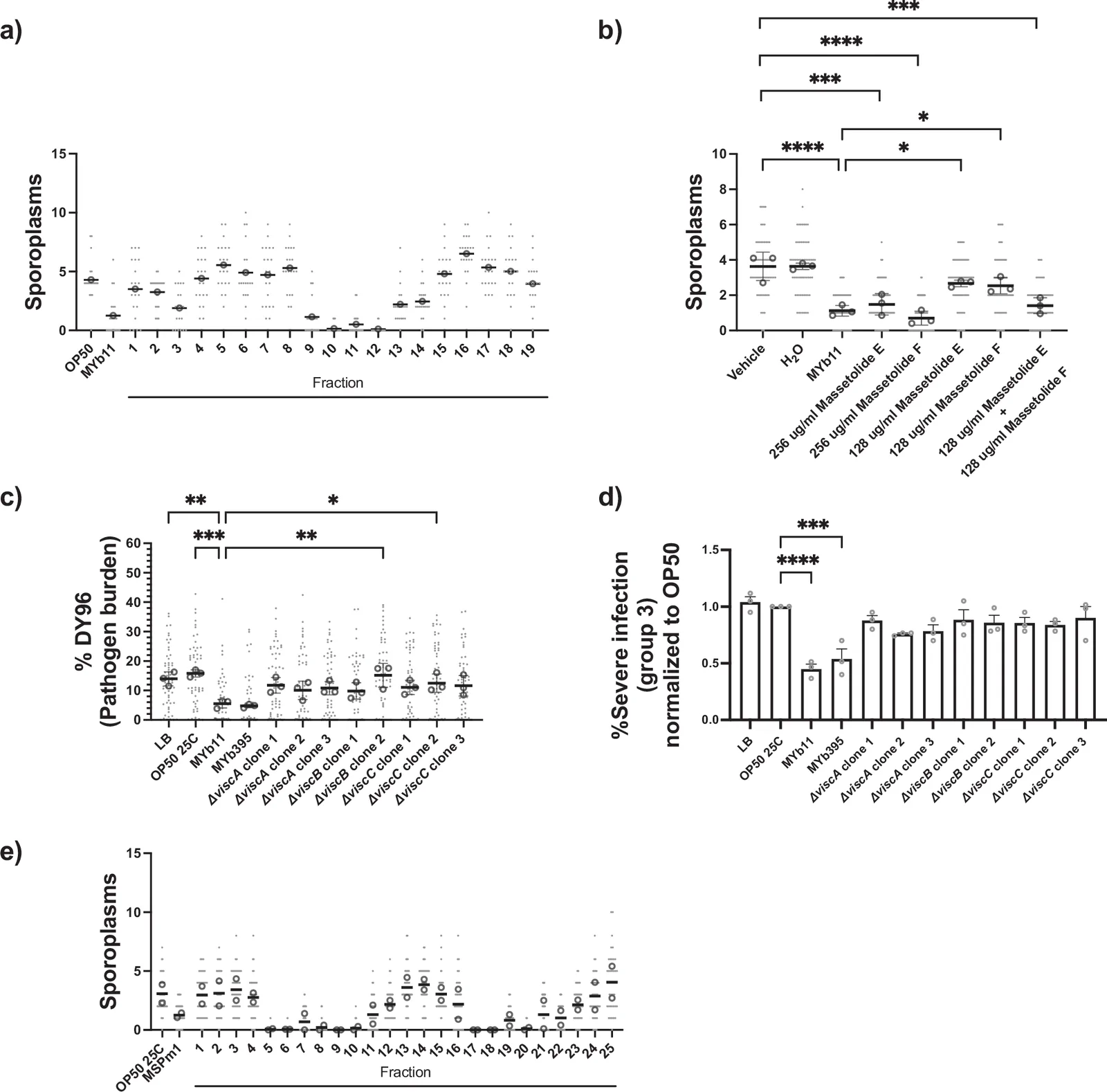
Massetolide E and F and two unknown molecules from *P. mendocina* MSPm1 exhibit anti-microsporidia activity. **a** Liquid chromatography of *P. lurida* MYb11 supernatant with a Sephadex LH-20 column resulted in 19 fractions that were tested with a 1-h infection to identify those containing activity. **b** Spores were incubated in either a vehicle control (0.5% DMSO), water, *P. lurida* MYb11 supernatant or massetolide E and/or F in various concentrations prior to L1 infection. The number of sporoplasms in L1 animals at 1 hpi was determined. *P*-values were determined via one-way ANOVA with Šidák’s multiple comparisons test. Each test condition was compared to the vehicle and each massetolide condition was compared to MYb11. **c**, **d** Spores were incubated in LB or supernatant from the bacterial strains denoted on the *X*-axis for 24 h. Synchronized L1 animals were infected for 72 h and then fixed in acetone and stained with DY96. Pathogen load was quantified (**c**) and the percentage of animals with severe infection, defined as spores covering over 50% of the body (classified as group 3, see Supplementary Fig. 9a) was determined and normalized relative to animals infected with spores incubated in *E. coli* OP50 supernatant (**d**). **e** Liquid chromatography of *P. mendocina* MSPm1 supernatant with a C18 column resulted in 25 fractions that were tested to identify those containing activity. The number of sporoplasms in L1 animals at 1 hpi was quantified. *P*-values determined via one-way ANOVA with Dunnett’s multiple comparisons test, with a single pooled variance (**c**–**d**). Each test condition was compared to) MYb11 (**c**) or to *E. coli* OP50 25 °C (**d**). Data are from *n* = 1 (**a**), *n* = 2 (**e**), or *n* = 3 (**b**–**d**) biological replicates of 100 worms (d) or 20 worms each (**a**–**c**, **e**). Mean ± SD represented by horizontal bars. Significance defined as * *p* < 0.05, ** *p* < 0.01, *** *p* < 0.001, **** *p* < 0.0001 and all *p*-values are included in the source data file.

*Pseudomonas* bacteria are known to produce a wide range of small molecules with antimicrobial activity^59^. To isolate active compounds present within the supernatants of *P. lurida* MYb11 and *P. mendocina* MSPm1, we conducted activity-guided purification followed by liquid chromatography-mass spectrometry. The *P. lurida* MYb11 fractions 9–12, separated by a Sephadex LH-20 column, displayed strong activity (Fig. 5a). Measurement of purified compounds from *P. lurida* MYb11 by mass spectrometry identified two molecules, massetolide E and F (Supplementary Fig. 8a, b). To test if massetolide E and F display anti-microsporidia activity, we incubated *N. parisii* in varying concentrations of these compounds. Although massetolide E and F do not significantly reduce the number of spores in the gut or alter spore firing, there is a reduction in the number of sporoplasms in intestinal cells (Fig. 5b and Supplementary Fig. 8c, d). To test if these two compounds display additive or synergistic effects, 128 μg/ml of each compound was added to *N. parisii* spores. Under these treatment conditions, spores were also less infective as seen by a reduced number of sporoplasms, similar to when each compound was added in isolation at 256 μg/ml. These results indicate that massetolide E and F display anti-microsporidia activity in an additive manner (Fig. 5b). To test if massetolide production is necessary for the anti-microsporidia activity of MYb11, we tested individual mutants in three genes of the viscosin biosynthetic gene cluster required for massetolide E production, *viscA* (CLM75_RS17745), *viscB* (CLM75_RS11010), and *viscC* (CLM75_RS11015). Mutations in any of the genes abolished anti-microsporidia activity, indicating that massetolides are necessary for the secreted anti-microsporidia activity of this bacterial isolate (Fig. 5c, d).

Cultured media from *P. mendocina* MSPm1 inhibits microsporidia spores in a similar fashion to *P. lurida* MYb11, however this strain is not predicted to produce massetolides (Supplementary Table 1). To identify what other anti-microsporidia compounds naturally exist, we performed activity-guided purification. The CombiFlash fractions showed activity in two different sets of fractions, suggesting the presence of two different molecules with anti-microsporidia activity. Two separate fraction intervals, 5–10 and 17–18, inhibit *N. parisii* spore infectivity as seen by a reduction in the number of sporoplasms when spores were incubated with these fractions (Fig. 5e).

### A diverse repertoire of *C. elegans*-associated *Pseudomonas* species display anti-microsporidia activity

*Pseudomonas* species (spp.) are frequently isolated from the native *C. elegans* habitat^8^. We next tested whether other species of *Pseudomonas* also produce anti-microsporidia compounds. We screened a set of 53 *Pseudomonas* spp. associated with *C. elegans* by incubating *N. parisii* spores in cultured media from these bacterial isolates^60–62^. Synchronized L1 N2 animals were infected with pre-treated spores for 72 h, and *N. parisii* infectivity was quantified by classifying nematodes as being lightly, moderately, or severely infected. We observed that 64% (34/53) of the *Pseudomonas* isolates secrete molecules which reduce the proportion of severely infected animals (Supplementary Fig. 9a, b).

**Fig. 6:**
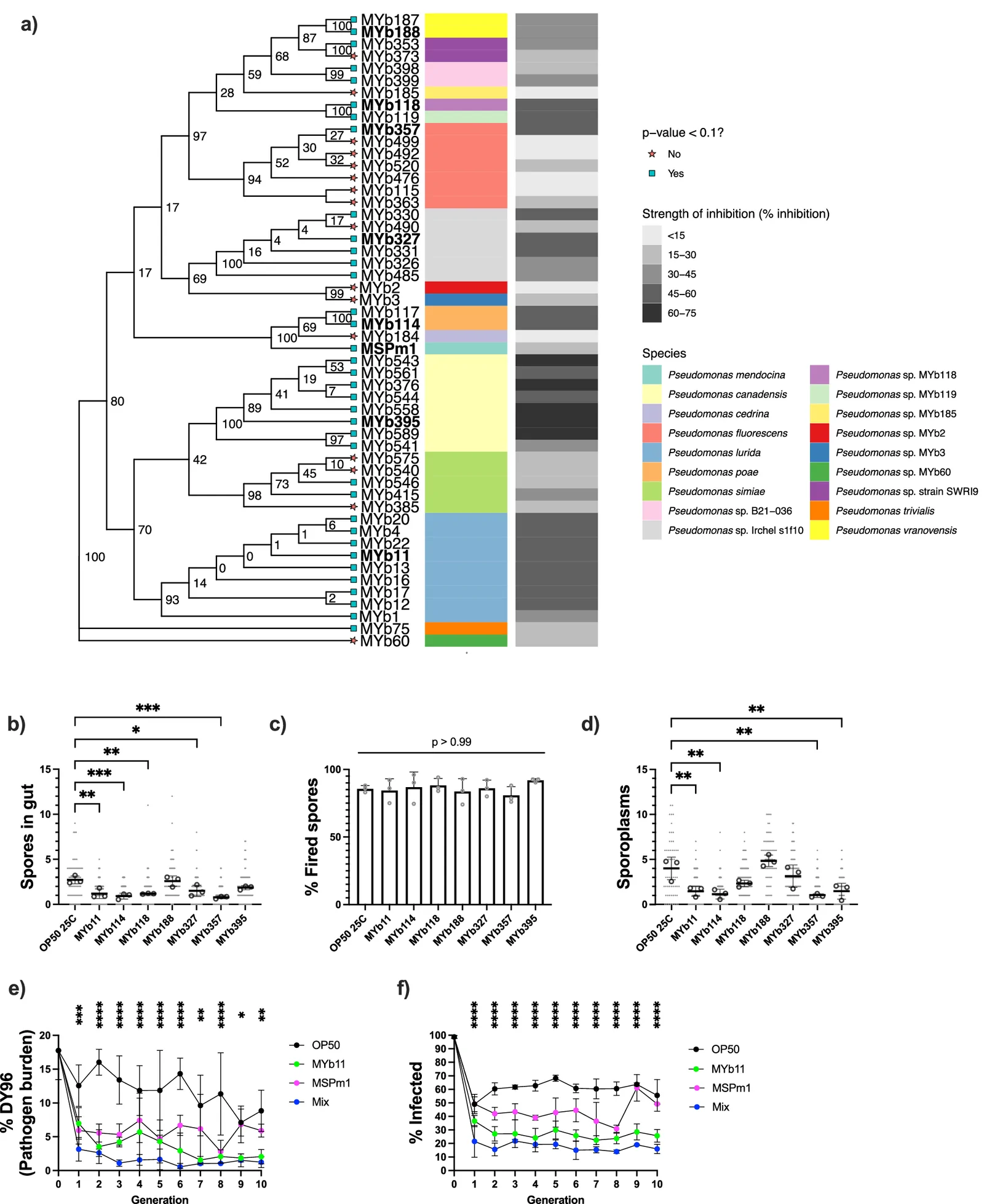
Many *Pseudomonas* species inhibit *N. parisii* spores and infection progression over multiple generations. **a** Phylogenetic tree demonstrating the evolutionary relatedness between *Pseudomonas* isolates using *rpoD* sequences. The isolates used in downstream experiments are bolded. The different *Pseudomonas* species are indicated using color coding. The strength of inhibition exhibited by the cultured media from each strain is indicated in varying shades of grey to black, where black indicates the highest strength of inhibition. Blue squares indicate significant inhibitory effects with *p* < 0.1. **b**–**d** L1 animals were pulse infected for one hour with *N. parisii* spores, incubated in supernatants of bacterial strains depicted on the *X*-axis. The number of spores (**b**), the fraction of fired spores (**c**), and the number of intracellular sporoplasms (**d**) were quantified. **e**, **f** L1 animals were infected for 72 h and 10 randomly selected gravid adult animals were transferred to various lawns of bacteria. After 4 days, 10 randomly selected gravid adult animals were transferred to fresh plates seeded with the same bacteria, while the remaining population was fixed and stained with DY96. This process was repeated for a total of 10 generations. Mix condition contains MYb11, MSPm1 and the six tested isolates from (**b**–**d**) in equal proportion. The spore burden (**e**) and the fraction of population containing spores (**f**) are depicted. All lawn conditions were compared to *E. coli* OP50 25 °C, and the significance between *E. coli* OP50 25 °C and Mix is indicated above the graphs. Data are from *n* = 3 biological replicates of at least 20 (**b**–**e**) or 100 (**f**) worms each and at least 50 spores (**b**, **c**). Mean ± SD represented by horizontal bars. *P*-values determined via ordinary one-way ANOVA (**b**–**d**) or ordinary two-way ANOVA (**e**, **f**) with Dunnett’s multiple comparisons test, with a single pooled variance. Each test condition was compared to *E. coli* OP50 25 °C. Significance defined as * *p* < 0.05, ** *p *< 0.01, *** *p* < 0.001, **** *p* < 0.0001 and all *p*-values are included in the source data file.

To determine the evolutionary relationship between species that displayed anti-microsporidia activity, we created a phylogenetic tree using *rpoD* sequences (Fig. 6a)^62,63^. We observed a distinct phylogenetic relationship between species with activity towards *N. parisii*. For example, all nine *P. lurida* isolates and all eight *P. canadensis* isolates we tested significantly inhibit infection. Conversely, only one out of seven *P. fluorescens* isolates tested has significant activity towards *N. parisii*. In total, we observed eight named species and at least 13 phylogenetically distinct groups of *Pseudomonas* with activity against *N. parisii*. To investigate the arsenal of secreted compounds produced by *C. elegans*-associated *Pseudomonas* species, we used antiSMASH analysis to identify candidate bacterial gene clusters from 21 genomes, including six we sequenced for this study^64^. All seven *P. lurida* isolates’ genomes are predicted with high confidence to produce viscosin, as was the one *P. canadensis* genome analyzed, MYb395. No other genomes are predicted to confidently make viscosin, though they are predicted to make a diversity of natural products, albeit with lower confidence (Supplementary Table 1). Together, these results suggest that a diverse group of *C. elegans*-associated *Pseudomonas* species commonly produce a variety of molecules which inhibit *N. parisii* infection.

In the wild, *C. elegans* are likely to be exposed to both *N. parisii* and *Pseudomonas* isolates, and molecules produced by these bacteria could act on spores as well as intracellular stages after *N. parisii* invasion. To determine mechanisms by which other *Pseudomonas* species inhibit *N. parisii*, we first incubated spores with conditioned media from six phylogenetically distinct *Pseudomonas* isolates and exposed L1 worms to these treated spores. The incubation of *N. parisii* in 5 of the 6 *Pseudomonas* isolates tested resulted in significantly fewer spores or sporoplasms in the nematode gut and no difference in the fraction of fired spores (Fig. 6b–d). We then set up continuous infections on lawns of the different *Pseudomonas* species, such that parasite invasion and proliferation are taking place in the presence of these bacteria. When animals were continuously infected, all *Pseudomonas* spp. resulted in improved host fitness with small, but nonsignificant, decreases in pathogen burden for some of the bacterial isolates (Supplementary Fig. 10).

### *Pseudomonas* species provide strong protection against *N. parisii* infection over multiple generations

Native microbiomes have the potential to limit infectious disease^65,66^. In the wild, *C. elegans* is often exposed to a variety of different bacteria^11,12,60,62^. As individually tested *Pseudomonas* species could reduce microsporidia infection, we asked if combining *Pseudomonas* species together may result in stronger effects. First, we tested if continuous infection of animals on lawns of *P. lurida* MYb11, *P. mendocina* MSPm1, or a combination of the two would similarly impact *N. parisii* infectivity. Host fitness was significantly improved when grown on single or mixed lawns relative to when grown on *E. coli* OP50 (Supplementary Fig. 11a). Growth on either *P. lurida* MYb11 or *P. mendocina* MSPm1 resulted in decreased parasite burden and combining these two strains did not result in a stronger protective effect (Supplementary Fig. 11b).

In natural environments, populations of *N. parisii*-infected *C. elegans* may come in contact with protective bacterial species and this could reduce infection levels of these *C. elegans* populations over multiple generations. To determine how continuous exposure to protective Pseudomonas isolates affects infection over successive cycles of parasite replication and transmission, we continuously infected L1 animals for 72 h and picked 10 randomly selected gravid animals from the same source plate onto lawns seeded with either *E. coli* OP50, *P. lurida* MYb11, *P. mendocina* MSPm1 or a mix of eight *Pseudomonas* species (MYb11, MSPm1, MYb114, MYb118, MYb188, MYb327, MYb357, and MYb395). Animals were propagated for 10 generations, and the pathogen burden and population infectivity were quantified at every generation to monitor infection progression. Growth on *E. coli* OP50 resulted in the highest levels of pathogen burden and ~60% of animals displaying infection over the course of the experiment (Fig. 6e, f). Animals grown on *P. lurida* MYb11, *P. mendocina* MSPm1 or the 8 species mix all showed a reduction in pathogen burdens and population infectivity. Although the pathogen burden continued to decline past generation 8 for *P. lurida* MYb11 and the mix conditions, a sharp increase was observed for those grown on *P. mendocina* MSPm1. Both *P. lurida* MYb11 and mix conditions resulted in continually low levels of *N. parisii* infection, and the mix condition resulted in the lowest levels of infectivity and only ~20% of the population producing spores (Fig. 6e, f). This experiment suggests that under conditions that more closely resemble those found in nature, where infected worms produce spores that infect other members of the population, *Pseudomonas* strains have a protective effect by reducing infection levels over multiple generations.

## Discussion

To understand how bacterial members of the native *C. elegans* microbiome impact nematodes infected with *N. parisii*, we utilized the recently established CeMbio collection of bacteria. Here, we demonstrate that bacterial species can alter *N. parisii* infection in multiple ways. First, we show that nutrient limitation can deprive *N. parisii* of the required host nutrients to fuel its growth. Second, *Pseudomonas* bacteria make molecules that target dormant spores, and growth on these bacteria reduces infection within populations for many generations. Altogether, this work demonstrates that the host bacterial microbiome can exert large impacts on microsporidia infection. Although the panel of bacteria we assayed covers a large extent of the known diversity of the *C. elegans* microbiome, other species and combinations of bacteria may also have an impact on microsporidia infection^10,12^. We also caution that although we observe protective effects that benefit host fitness in a controlled laboratory environment, other factors that we are unable to account for could impact the fitness of *C. elegans* in the wild.

Microsporidia infection can be influenced by host diet^67–69^. We observed that worms grown on *S. multivorum* BIGb0170 or *C. scophthalmum* JUb44 display increased microsporidia invasion, which likely occurs by increased pumping in the nematodes due to a poor-quality food source^12,70^. Despite this initial increase in pathogen numbers, the ability to sporulate is delayed on these bacteria, and lipidomics revealed that these diets caused a disruption to host unsaturated lipid levels. Microsporidia infection in multiple animals has been shown to result in decreased fatty acid levels, including reduced linoleic acid in the gypsy moth^67,71–73^. Fatty acid supplementation of either palmitic or oleic acid in *Drosophila melanogaster* resulted in increased proliferation of the microsporidian *Tubulinosema ratisbonensis*^67^. Protection against pathogenic bacteria by beneficial bacteria through alterations of host sphingolipid levels has also been observed in *C. elegans*^74^. We found that the PUFA fatty acid linoleic acid can restore *N. parisii* infectivity in nematodes grown on *S. multivorum* BIGb0170 and depletion of linoleic acid through the loss of the *C. elegans* desaturase FAT-2 delays *N. parisii* sporulation, suggesting an important requirement of PUFA fatty acids for microsporidia infection. Thus, while infected nematodes grown on *S. multivorum* BIGb0170 or *C. scophthalmum* JUb44 display overall reduced pathogen loads, this is likely due to the poor growth conditions of the host. Given the extreme host dependence displayed by microsporidia, poor host resources may not enable robust *N. parisii* growth, thereby indirectly protecting the host from infection. Although our data support the role of linoleic acid as being important for *N. parisii* sporulation, the mechanism by which *S. multivorum* BIGb0170 alters lipid levels and its effects on linoleic acid levels remain to be determined.

Bacteria can inhibit microsporidia infection either through activating host immunity or by producing molecules which directly act on microsporidia. Infection with microsporidia induces a transcriptional program called the intracellular pathogen response, and none of 71 bacterial strains associated with *C. elegans* activated a reporter of this immune response^17,75–77^. From this result, we speculate that immune activation by the microbiome may play a minor role in protection against microsporidia infection of *C. elegans*. In contrast, we observed that 2 out of the 11 CeMbio species tested produce molecules that cause an ~twofold to fourfold reduction in invasion. Both of these inhibitory bacteria are *Pseudomonas* species and 65% of *Pseudomonas* isolates we tested make molecules that have activity against *N. parisii* spores. About 22% of all isolated *C. elegans*-associated bacteria are *Pseudomonas*, which suggests an estimate of about 14% of the bacteria *C. elegans* encounters in the wild provide protection against *N. parisii* spores^11,12,60^. We also observed that *Pseudomonas* isolates can inhibit *N. parisii* proliferation, suggesting that compounds produced by these bacteria can act on intracellular stages of microsporidia. Although many *Pseudomonas* species are pathogenic against *C. elegans*, of the eight isolates with activity against microsporidia, only two caused a reduction in *C. elegans* fitness, suggesting that many *Pseudomonas* species could have a beneficial effect on *C. elegans* in the wild. These *Pseudomonas* bacteria also produce vitamin B12, which provides tolerance to *N. parisii* infection^34,62^. As bacteria seldom exist in isolation, we mimicked natural conditions by growing infected nematodes on lawns containing a mixture of bacteria and showed that a mixture of *Pseudomonas* could provide robust protection against *N. parisii* for multiple generations.

*Pseudomonas* species produce many molecules with antimicrobial activities^59,60^. We identified that *P. lurida* MYb11 produces massetolides E and F, which are cyclic lipopeptides related to viscosin that have been demonstrated to have wide antimicrobial activity with effects on viruses, protozoa, oomycetes and bacteria^13,78–82^. *P. lurida* MYb11 has been previously reported to produce massetolide E, which was shown to display potent antibacterial activity against the gram-positive bacterial pathogen *B. thuringiensis*^13^. The mode of action of many cyclic lipopeptides occurs through disruption of target cell membrane integrity, resulting in cell death^83,84^. This process has been well documented in oomycetes, such as massetolide A inducing zoosporicidal activity against the late blight plant pathogen *Phytophthora infestans*^84^. We demonstrate that massetolide E and F impact *N. parisii* spores through a reduction in the number of spores, which is an inhibitory mechanism that has been observed with other molecules such as porphyrins^57^. Several species of gram-positive *Bacilli* bacteria have been shown to produce molecules that display antimicrobial activity towards microsporidia, suggesting that bacterial inhibition of microsporidia might be common^37,85,86^.

Microsporidia are widespread parasites of agriculturally important animals and are a threat to food security, but there are limited approaches to treating or preventing these infections. The most commonly used anti-microsporidia drugs suffer from either host toxicity or limited activity against some species^22^. *C. elegans* has been used to identify novel microsporidia inhibitors from small molecule libraries, and we now show that this model organism can also be used to identify bacteria that produce anti-microsporidia compounds^87,88^. There is an interest in using bacteria to combat infections, and probiotics have been used in honey bees to reduce microsporidia infections, though with mostly modest results^89^. The demonstration that *Pseudomonas* bacteria produce a diversity of molecules with activities against microsporidia provides a potential starting point to harness these bacteria to control microsporidia infections.

## Methods

### Ethical statement

All research was conducted in accordance with applicable ethical and biosafety regulations. As *C. elegans* is not subject to animal-welfare regulations, animal ethics approval was not required. Experiments involving *C. elegans*, microsporidia, and bacteria were conducted under biosafety permit 169-R20-2, approved by the University of Toronto Institutional Biosafety & Biosecurity Committee.

### Strain maintenance

*C. elegans* strains were maintained at 21 °C on nematode growth medium (NGM) plates seeded with 10× *E. coli* OP50-1. For all infection assays, 15 L4 animals were picked onto a 10-cm seeded NGM plate. 4 days later, heavily populated plates were bleach synchronized^90^. Embryos were hatched overnight in 5 ml of M9 at 21 °C. L1 animals were used no later than 20 h post-bleaching. For a list of strains utilized in this study, refer to Supplementary Table 2. All animals used in this study were hermaphrodites, and the stage of the animals is described for each experiment in the figure legends.

For 72 h of growth on CeMbio strains, 1000 synchronized L1 animals were grown on 6-cm NGM plates seeded with individual microbiome strains and incubated at 21 °C. 24 (Supplementary Fig. 1a–d) or 72 h later (Fig. 1), animals were washed off with M9 + 0.1% Tween-20 into individual microcentrifuge tubes. These animals were then washed twice more with M9 + 0.1% Tween-20 to remove residual bacteria from the supernatant.

### Bacterial growth and maintenance

*E. coli* OP50-1, CeMbio strains and *C. aquatica* DA1877 were struck out onto lysogeny broth (LB) agar and incubated at 25 °C for 24 or 48 h (BIGb0170, BIGb0172, MYb1, MYb115, and MYb185). Colonies were kept for a maximum of two weeks at 4 °C. JUb134 was excluded as it did not grow at a similar rate to other strains in overnight cultures. Individual colonies were then picked into 5 ml of LB or 2× Tryptic soy broth (TSB) (MYb357) and cultured overnight at 25 °C for 16–18 h at 220 RPM. Cultures were then adjusted to an OD_600_ of 1.0 using a Microspek^TM^ DSM3 cell density meter. Cultures were either diluted in LB or concentrated via centrifugation at 7197 × *g* for 5 min. 180 μl of culture was used to seed a 6-cm NGM plate and 450 μl (Lipidomics) or 1 ml (10 generation experiments) for a 10-cm NGM plate. For experiments involving *E. coli* OP50 supplementation, 6-cm NGM plates were seeded with 180 μl in a 1:1 ratio of *E. coli* to JUb44 or BIGb0170 (90 μl each). Seeded plates were incubated at 25 °C for 24 h (or 4 days when 1 ml of culture was used) and stored at 4 °C until use. Although we adjust cell density of bacteria, once we seed the NGM plates with bacteria the density of the bacteria is no longer being controlled for and different bacterial isolates will have different amounts of bacteria that the worms are exposed to. Freshly seeded plates were used for every experimental replicate except in the case of multigenerational experiments (see below). *E. coli* OP50-1 at 37 °C represents a culture grown at 37 °C and saturated 10×. This was also used at an OD_600_ of 1.0, and was adjusted with LB. This served as a control for *E. coli* OP50 cultured at 25 °C.

### Dietary supplementation

Fatty acids were supplemented post-autoclave to standard 6-cm NGM plates at 0.8 mM^91^. Briefly, saturated fatty acids (palmitic and stearic acid) were resuspended in 100% ethanol and heated at 65 °C to dissolve. NGM was supplemented, prior to autoclaving, with tergitol at a final concentration of 0.1% for ethanol, palmitic and stearic acid supplemented plates. Ethanol plates were prepared at a final concentration of 0.5%. NGM flasks were weighed prior to autoclaving and brought back to their starting weight with autoclaved water once sterilized. Plates were stored at 4 °C until use. Seeded plates were incubated with desired bacterial species at 25 °C for 24 h to promote bacterial uptake of fatty acids prior to the addition of *C. elegans*. Palmitic acid [Sigma-Aldrich P0500], stearic acid [Sigma-Aldrich 175366], oleic acid [Sigma-Aldrich O1008], linoleic acid [Sigma-Aldrich L1376] and tergitol [Millipore Sigma-NP40S] were utilized in this study.

### L1 pulse infection

For 1 h pulse, 71-h chase experiments either 25,000 bleach-synchronized N2 L1 animals were infected with a high dose of *N. parisii* (ERTm1) spores (Fig. 3) or 6000 bleach synchronized N2 L1 animals were infected with a medium dose of *N. parisii* spores (Supplementary Figs. 5 and 6b, c). In both circumstances, 10 μl 10× *E. coli* OP50-1, or a mock treatment of an equivalent volume of M9 instead of spores (uninfected) was added. Worms, *N. parisii* spores/M9, and *E. coli* were placed in a microcentrifuge tube and mixed via pipetting prior to plating on an unseeded 6-cm NGM plate. After one hour, worms were washed off plates using 700 μl of M9 + 0.1% Tween-20 and placed into a microcentrifuge tube. Animals were then washed twice in M9 + 0.1% Tween-20 to remove residual spores in the supernatant prior to evenly splitting the worm population onto seeded plates of choice.

For L1 pulse infections with spores incubated in supernatants (Figs. 4b–g and 6b–d and Supplementary Figs. 7 and 9b), fractions (Fig. 5a, d), or compounds (Fig. 5a, e), 1000 bleach synchronized N2 L1 animals and 10 μl 10× *E. coli* OP50-1 were added to the microcentrifuge tube containing *N. parisii* spores and supernatant, fraction or compound and plated onto an unseeded 6-cm NGM plate.

Spore doses used in this study are listed in Supplementary Table 3

### Adult pulse infection

Seventy-two-hour old animals were infected by adding a medium dose of *N. parisii* spores and 10 μl of saturated 10× OP50-1 into the microcentrifuge tube containing the adult animals. This mixture was gently pipetted up and down prior to placing it onto an unseeded 6-cm NGM for one hour at 20 °C. Animals were then washed off with M9 + 0.1% Tween-20. Two additional M9 + 0.1% Tween-20 washes were performed to remove residual spores that have not been ingested. A fraction of these animals was set aside for fixation (representing 1 hpi) and the rest were placed onto a 6-cm NGM plate seeded with the corresponding bacteria on which they were grown. 24, 48, or 72 h later, animals were washed off with M9 + 0.1% Tween-20 and fixed in acetone for downstream FISH and DY96 staining.

### Quantifying pharyngeal pumping

Seventy-two-hour old animals were picked onto 6-cm *E. coli* OP50 seeded NGM plates immediately prior to measurement. Pumping was measured for one minute per animal using an Axio Zoom V.16 (Zeiss).

### Lipidomics

2500 N2 L1 animals were placed onto each of two 10-cm plates seeded with the desired bacterial species (450 µl) for 72 h at 20 °C. Three biological replicates were included for each condition. All animals or bacteria were then washed with 1 ml of M9 into a microcentrifuge tube. For samples containing nematodes, animals were washed to remove residual bacteria from the sample. All samples were then flash frozen in liquid nitrogen and stored at −80 °C until use in extraction.

Lipids were extracted from pelleted and frozen animals using Bligh-Dyer extraction^92^ in a bead mill homogenizer. Samples were first mixed via vortexing and pipette agitation and resuspended in ice-cold methanol. One volume of methanol-sample mixture was transferred to one volume of ice-cold chloroform in beadmill homogenizer tubes. Then, accounting for water content in the initial sample, water was added to bring the final water content to 0.9 volumes. Following homogenization and phase-separation by centrifugation, lower organic layers were collected for lipidomics and dried in a vacuum evaporator.

The dried organic layer was resuspended in 50 µL of 1:1 AcN:IPA (v/v). Reconstituted extracts were analyzed with a Vanquish dual pump liquid chromatography system coupled to an Orbitrap ID-X (Thermo Fisher Scientific) using a H-ESI source in positive mode. All samples were injected at 2 μL and analytes separated with 30 min reversed-phase chromatography (Accucore C30 column; 2.6 μm, 2.1 × 150 mm; 27826-152130; Thermo) with an Accucore guard cartridge (2.6 μm, 2.1 × 10 mm, 27826-012105, Thermo). Mobile phase A consisted of 60% LC/MS grade acetonitrile (ACN) (A955, Fisher). Mobile phase B consisted of 90% LC/MS grade isopropanol (A461, Fisher) and 8% LC/MS grade ACN. Both mobile phases contained 10 mM ammonium formate (70221, Sigma) and 0.1% LC/MS grade formic acid (A11710X1-AMP, Fisher). Column temperature was kept at 50 °C, flow rate was held at 0.4 mL/min, and the chromatography gradient was as follows: 0–1 min held at 25% B, 1–3 min from 25% B to 40% B, 3–19 min from 40% B to 75% B, 19–20.5 min from 75% B to 90% B, 20.5–28 min from 90% B to 95% B, 28–28.1 min from 95% B to 100% B, and 28.1–30 min held at 100% B. A 30 min wash gradient was run for every column injection to clean up the column and re-equilibrate solvent conditions that went as follows: 0–10 min held at 100% B and 0.2 mL/min, 10–15 min from 100% B to 50% B and held at 0.2 mL/min, 15–20 min held at 50% B and 0.2 mL/min, 20–25 min from 50% B to 25% B and held at 0.2 mL/min, 25–26 min held at 25% B and ramped from 0.2 mL/min to 0.4 mL/min, and 26–30 min held at 25% B and 0.4 mL/min. Mass spectrometer parameters were: source voltage 3250 V, sheath gas 40, aux gas 10, sweep gas 1, ion transfer tube temperature 300 °C, and vaporizer temperature 275 °C. Full scan data was collected on experimental replicates using the orbitrap with scan range of 200-1700 m/z at a resolution of 240,000 FWHM. On pooled samples for lipid identification, primary fragmentation (MS2) was induced in the orbitrap with assisted HCD collision energies at 15, 30, 45, 75, 110%, CID collision energy was fixed at 35%, and resolution was at 15,000. Secondary fragmentation (MS3) was induced in the ion trap with rapid scan rate and CID collision energy fixed at 35% for 3 scans. LipidSearch (v 5.0, Thermo) was used for lipid annotation and used to generate a mass list for peak picking and integration of experimental replicates in Compound Discoverer (v 3.3)^93^. Heatmaps were generated via the pheatmap R package^94^.

### Sample fixation and staining

Samples were washed off plates with 1 ml of M9 + 0.1% Tween-20 into a microcentrifuge tube. They were then washed once more before adding 700 μl of acetone to worm pellets. DY96, a chitin binding dye, was used to assess parasite burden (microsporidia) and worm embryos. 500 μl of DY96 solution (1 × PBST, 0.1% SDS, 20 μg/ml DY96) was added to worm pellets that had been fixed in acetone. Samples were left to rock in the dark for 30 min at room temperature and then centrifuged to remove the dye. Worms were then resuspended in 20 μl of EverBrite Mounting Medium (Biotium) and 10 μl was mounted onto glass slides for imaging. To observe early intracellular infection events (sporoplasms), the MicroB FISH probe (5′-ctctcggcactccttcctg-3′) conjugated to Cal Fluor 610 (LGC Biosearch Technologies) was used to bind the 18S rRNA of *N. parisii*. Animals fixed in acetone were washed twice in 1 ml PBST, followed by a single 1 ml wash in Hybridization buffer (0.01% SDS, 900 mM NaCl, 20 mM TRIS pH 8.0). Samples were then incubated overnight at 46 °C in the dark with 5 ng/μl of the MicroB FISH probe in 100 μl of hybridization buffer. Samples were then washed in 1 ml of wash buffer (hybridization buffer + 5 mM EDTA), and incubated in 500 μl of wash buffer for 30 min at 46 °C. To visualize intracellular stages (sporoplasms and meronts) alongside spores, the final incubation was replaced with 500 μl of DY96 solution for 30 min at room temperature. The supernatant was then removed, and samples were resuspended in 20 μl of EverBrite Mounting Medium (Biotium).

### Spore firing assays

Animals stained with FISH and DY96 were used to determine the fraction of fired spores. FISH^+^ DY96^+^ spores represent unfired spores, whereas FISH^−^DY96^+^ spores represent fired spores. FISH^+^DY96^−^ events represent intracellular sporoplasms. Percentage of fired spores is defined as the proportion of fired spores over the total number of fired and unfired spores within a population.

### Microscopy and Image quantification

All imaging was performed using an Axio Imager.M2 (Zeiss), except for quantification of *Pacdh-1::*GFP in Supplementary Fig. 6a, which was imaged using an Axio Zoom V.16 (Zeiss) at a magnification of 45.5×. Images were captured via Zeiss ZEN 2.3 software and quantified under identical exposure times per experiment. Gravidity is defined as the presence of at least one embryo per worm and the fraction of gravid adults is defined as the number of animals containing at least one embryo over the total number of animals quantified. Animals were considered infected if clumps of newly formed spores (48–72 hpi) were visible in the body of animals as seen by DY96. FISH-stained animals were considered infected if at least one sporoplasm was visible in intestinal cells.

To quantify fluorescence within animals (pathogen burden), regions of interest were used to outline every individual worm from anterior to posterior. Individual worm fluorescence from DY96 or FISH staining were subjected to the “threshold” followed by “measure” tools in FIJI 2.1.0^95,96^.

### In vitro spore incubation

Overnight cultures of bacterial strains were grown as described above. Cultures were then centrifuged at 7197 × g for 5 min, and 100 μl of supernatant, massetolide or fractions were placed into a microcentrifuge tube with a medium dose of *N. parisii* spores at 20 °C for 21–24 hours. The entire volume was used for downstream experiments. For continuous infections using wild *Pseudomonas* isolates, a low dose of *N. parisii* spores was used (Supplementary Fig. 10).

Massetolide E and F were resuspended in 100% DMSO to a final concentration of 5 mg/ml and stored at −20 °C. 256 μg/ml and 128 μg/ml concentrations were generated by adding 5.12 and 2.56 μl of 5 mg/ml stocks, respectively in a final volume of 100 μl of nuclease-free water. Vehicle controls contained 5 μl of 100% DMSO in 95 μl of nuclease-free water. H_2_O control represents spores incubated in 100 μl of nuclease-free water.

To wash spores incubated in bacterial supernatant, samples were centrifuged at 12,000 × *g* for 1 min. Spores were washed twice in 500 μl of autoclaved water and resuspended in the same volume as the unwashed samples.

### Seventy-two hour continuous infections

1000 bleach synchronized L1 animals and 400 μl of 10× *E. coli* OP50-1 were added to the microcentrifuge tube containing the supernatant-spore incubations (Fig. 4b and Supplementary Fig. 7). Samples were then plated onto unseeded 6-cm NGM plates for 72 h at 21 °C. Animals were washed off with 1 ml of M9 + 0.1% Tween-20 and fixed in acetone. When using seeded plates (Supplementary Fig. 11), 1000 bleach synchronized L1 animals and a high dose of *N. parisii* spores was added to a final volume of 200 μl in M9 and plated onto a seeded 6-cm NGM plate.

### Propidium Iodide staining

PI (P4170-Sigma Aldrich) was added to samples at a final concentration of 1 μg/ml after overnight incubation in bacterial supernatants. Heat-killed spores were generated as a positive control for PI staining by incubating spores in 100 μl of LB at 65 °C for 10 min.

### Quantifying spore concentrations

4 μl of Calcofluor white was added to pre-incubated spores and mixed via pipetting. 4 μl of this sample was then mounted onto a Cell-Vu slide (Millenium Sciences DRM600). The number of spores in 10 squares of the grid was measured three independent times and averaged to represent a single biological replicate. Spore concentration was calculated as described by the manufacturer (number of spores in 10 grids/2 = million spores/ml). Spore samples were blinded prior to quantification.

### Purification of anti-microsporidia compounds

*P. lurida* MYb11 and *P. mendocina* MSPm1 were grown in TSB medium. 50 ml of inoculum in TSB was used for inoculating a 1-liter culture. Both the stains were grown at 25 °C at 220 rpm. After 48 h, the supernatant was separated from the cell by centrifugation at 7500 × *g* for 20 min. The cell-free supernatant was added to 6–8% (w/v) of activated Diaion HP-20 (Sigma) resin. This mixture was allowed to mix for 2–3 h. The resins were separated and washed with distilled water. The compounds that were bound to the resin were eluted with 100% methanol. The solvent was evaporated using a rotary evaporator (RotaVap, Heidolph). The extract was reconstituted in Milli-Q water. The extract was loaded onto a Sephadex LH-20 column and eluted using 50% methanol in isocratic mode. The fractions were collected and concentrated using GENEVAC evaporator. The fractions were reconstituted using Milli-Q water and the activities were measured as described above. The active fractions were pooled and loaded on reverse-phase CombiFlash ISCO column (RediSep Gold Rf C18, Teledyne). The compounds were eluted with ACN and water with 0.1% formic acid using a gradient of 5 to 95% ACN. The fractions were concentrated and the activities were assessed. The presence of massetolide E and massetolide F from the *P. lurida* MYb11 strain was detected by using mass spectrometry in the CombiFlash fractions. The active fractions were dried and then applied to an HPLC C28 Analytical column (Sunniest RP-AquA C28 4.6 × 100 mm, 5 µm) using water and eluted with a gradient of ACN. Both solvents contained 0.1% formic acid. The fractions were collected and the activity was assessed. The active peaks eluted after 95% of ACN. The pure compounds, massetolide E and massetolide F, were lyophilized to generate a white powder.

In the case of *P. mendocina* MSPm1, the CombiFlash fractions indicated the presence of two different compounds exhibiting anti-microsporidia activity. The two compounds were different in terms of their polarity. The polar compounds eluted in earlier fractions of CombiFlash and the non-polar compounds eluted later. The yield of these compounds was very low. To mitigate this, we scaled up the fermentation and inoculated six liters of TSB with *P. mendocina* MSPm1. After 48 h of incubation, we prepared and processed the extract as described earlier. The polar fractions with activity were pooled and injected into HPLC on a C28 analytical column (Sunniest RP-AquA C28 4.6 × 100 mm, 5 µm) using water and eluted with a gradient of ACN with 0.1% formic acid. We collected seven peaks separately, but did not get any significant activities. To overcome this, we decided to collect the individual peaks in multiple injections to get enough compound (at least 1 mg each) to test the activity and identify the active compound. The non-polar fractions also showed decreased antibacterial activity. After several attempts of HPLC (analytical column X Select® C18 4.6 × 100 mm, 5 µm) injections, we were unable to recover material for further experimentation.

### Mass spectrometry

The mass of massetolide E and massetolide F were analyzed using HR-ESI-MS recorded with Infinity II LC System (Agilent Technologies) coupled with a qTOF 6550 mass detector in positive ion mode. The compounds were dissolved in DMSO and ran on qTOF using an Agilent C8 column (Eclipse XDB-C8 2.1 × 100mm, 3.5 µm) using water and ACN with 0.1% formic acid as solvents.

### Generation of MYb11 mutants

A two-step allelic exchange protocol^97^ was used to generate MYb11 knock-out mutants *viscA* (CLM75_RS17745), *viscB* (CLM75_RS11010), and *viscC* (CLM75_RS11015). Approximately 700 bp PCR amplicons flanking each target gene were cloned into the pUISacB vector and transformed into competent *E. coli* cells. The constructed plasmid was then transferred into MYb11 recipient strains by tri-parental conjugation using a helper *E. coli* strain carrying the plasmid pRK2013^98^. Successful transconjugants were initially selected using tetracycline and nitrofurantoin, followed by counter-selection on sucrose-containing medium. Deletion mutations were confirmed by Sanger sequencing.

### *rpoD* gene amplification and sequencing

1 ml of overnight culture was used for genome extraction of most of the *Pseudomonas* isolates using a bacterial genomic DNA extraction miniprep kit (Bio Basic-BS423) following the manufacturer’s instructions except for modifying the incubation time of buffer-treated bacteria to 2 h at 56 °C. Centrifugation steps were modified to 3 min at 21, 130 × g, or 9000 × *g*. DNA was eluted with 35 µl nuclease-free H_2_O. Extracts were stored at −20 °C. In the case of MYb476, MYb492, MYb520, MYb522, MYb543, and MYb544, single colonies of each strain were resuspended in 50 µl Milli-Q water in 1.7 ml Eppendorf tubes. The tubes were immersed in liquid nitrogen and then incubated at 70 °C three times. Lysates were stored at −20 °C.

*rpoD* was then amplified using psEG30F (5′-ATYGAAATCGCCAARCG-3′) and psEG790R (5′-CGGTTGATKTCCTTGA-3′) and KAPA2G Fast Hotstart Readymix (Roche- 07960956001) to generate a 736 bp product^99^. 1 µl of 50 ng/µl whole genomic DNA or 1–2 µl of lysate was used as the PCR template. Amplicons were then PCR purified using a Monarch® PCR & DNA cleanup kit (NEB-T1030S) once a band of the correct size was visualized on a 1% agarose gel. Sanger sequencing using the psEG790R primer was performed and high-quality ~600 bp amplicons were used to generate the phylogenetic tree. *rpoD* sequences for MYb114 (NZ_PCQN01000019.1), MYb117 (NZ_PCQL01000021.1), MYb184 (NZ_PCQE01000032.1), and MSPm1 (NZ_CP059139.1) were acquired from NCBI.

### Species designation

*rpoD* sequences from each isolate were queried using basic local alignment search tool (BLAST https://blast.ncbi.nlm.nih.gov/Blast.cgi). Isolates were assigned a species group based on the following parameters: In the case where both NCBI and BLAST displayed different species predictions, those from NCBI were used. If NCBI did not assign a species name (strain only), then the species designation based on BLAST results with *a* > 98% match to a whole genome sequence was used. If no match to a whole genome sequence was found via BLAST but NCBI provided a strain name, the species was determined via a BLAST match of >98% to the *rpoD* sequence. For MYb60 there was a 100% match to a single *P. fluorescens rpoD* sequence, but this isolate clustered separately from the other *P. fluorescens* isolates during phylogenetic analysis (below) and was therefore labelled as *P. sp*. MYb60.

### Phylogenetic analysis

ModelTest-NG v0.1.7 was run on the FASTA alignment to estimate the best RAxML model. AIC and AICc statistical criteria suggested the general time reversible model (GTR + G4)^100^. Therefore, the phylogenetic tree was constructed with RAxML-NG v. 1.2.1 using the GTR + G4 model and the bootstrap values were calculated based on 1000 replications^101^. Finally, the tree was rooted with MYb60 and plotted as a dendrogram using R packages ape v5.7.1^102^, ggtree v3.10.0^103–107^, ggplot2 v3.4.4^108^, ggnewscale v0.4.9^109^, ggtreeExtra v1.12.0^103,110^, ggstar v1.0.4^111^, and RColorBrewer v1.1.3^112^.

### Whole genome sequencing

Previously isolated genomic DNA from MYb114, MYb118, MYb188, MYb327, MYb357, and MYb395 were submitted for whole genome sequencing at Plasmidsaurus (https://www.plasmidsaurus.com/). Bacterial genome sequencing was performed by Plasmidsaurus using Oxford Nanopore Technology with custom analysis and annotation.

### Analysis of bacterial secondary metabolites

Genome assemblies were uploaded onto antiSMASH (https://antismash.secondarymetabolites.org/#!/start) and ran using default parameters^64^.

### Multigeneration continuous infections

Prior to the start of the experiment, unseeded 10-cm NGM plates were seeded with 1 ml of OD 1.0 *E. coli* OP50 or MYb11 or MSPm1 or an even mixture of MYb11, MSPm1, MYb114, MYb118, MYb188, MYb327, MYb357, and MYb395—“Mix” (250 μl of each). Plates were incubated at 25 °C for 4 days to promote lawn growth and stored at 4 °C until use. The plates used to grow generation 1–7 were seeded with cultures originating from a single colony, whereas those for generation 8–10 were seeded using cultures from a different colony. 1000 synchronized N2 L1 animals, 400 μl of 10× *E. coli* OP50-1 and a very low dose of *N. parisii* spores were combined in a centrifuge tube, evenly mixed, and pipetted onto an unseeded 6-cm NGM plate. Plates were incubated at 20 °C for 72 h. 10 randomly selected gravid adult animals were then picked onto seeded 10-cm NGM plates with the desired bacteria. Every 4 days (representing a single generation), 10 randomly selected gravid adult animals were passaged onto new seeded plates of the same bacteria, while the rest of the plate was washed and fixed for imaging. At the 10th passage, animals were grown for 4 days and then fixed and stained for imaging. At every generation, the pathogen load in at least 20 animals was measured (% DY96), as well as the fraction of the population containing *N. parisii* spores (% infected).

### Statistics and reproducibility

All data analysis was performed in GraphPad Prism 10.0. The statistical test used in each experiment is described in the corresponding figure legend.  Statistical significance defined as *p* < 0.05 with the exception of Fig. 6a and Supplementary Fig. 9b, where significance was defined as *p* < 0.1. The mean and standard deviations were calculated based on three individual biological replicates. Normality of data was assessed using ANOVA residuals. All data was considered normal by at least one test, and the results of this analysis are in Supplementary Table 4.

For all quantified data where statistics was performed *n* = 3 biological replicates. The number of replicates and animals analyzed for each replicate were chosen based on ours and others previous publications using similar assays. For *C. elegans* experiments, each biological replicate is made up of 10–100 individual animals, depending on the experiment, which are sample sizes used in previous studies^46,53,87^. No data was excluded from the analysis of any experiments. For almost all experiments, replication of experiments was successful, with at least three biological replicates for each experiment. The one exception were testing fractions of *P. lurida* MYb11 and *P. mendocina* MSPm1, for which only one or two replicates were performed. For all experiments, animals were taken from a large population of identical individuals, and randomly assorted into different treatment conditions. For infection experiments with spores, aliquots were taken from a pool and randomly distributed into different sample groups. Blinding was not performed for most experiments in this study. For microscopy-based assays, blinding was not necessary as clearly defined scoring criteria was used to manually score various phenotypes. The one exception is Fig. 4h, where counting differences in spores could be biased by knowing the identity of the samples, so all of these samples were blinded prior to quantification.

### Reporting summary

Further information on research design is available in the Nature Portfolio Reporting Summary linked to this article.

## Supplementary information

Supplementary Information

Description of Additional Supplementary Files

Supplementary Data 1

Supplementary Data 2

Reporting Summary

Transparent Peer Review file

## Source data

Source Data

## Data Availability

The *rpoD* sequences generated in this study are available from NCBI under the following accession codes: PP861230, PP861231, PP861232, PP861233, PP861234, PP861235, PP861236, PP861237, PP861238, PP861239, PP861240, PP861241, PP861242, PP861243, PP861244, PP861245, PP861246, PP861247, PP861248, PP861249, PP861250, PP861251, PP861252, PP861253, PP861254, PP861255, PP861256, PP861257, PP861258, PP861259, PP861260, PP861261, PP861262, PP861263, PP861264, PP861265, PP861266, PP861267, PP861268, PP861269, PP861270, PP861271, PP861272, PP861273, PP861274, PP861275, PP861276, and PP861277. Genome assemblies generated for the sequenced *Pseudomonas* strains are available from NCBI under the following accession codes: GCA_040438915.1 [https://www.ncbi.nlm.nih.gov/assembly/GCA_040438915.1/], GCA_040438925.1 [https://www.ncbi.nlm.nih.gov/assembly/GCA_040438925.1/], GCA_040438935.1 [https://www.ncbi.nlm.nih.gov/assembly/GCA_040438935.1/], GCA_040438945.1 [https://www.ncbi.nlm.nih.gov/assembly/GCA_040438945.1/], GCA_052112365.1 [https://www.ncbi.nlm.nih.gov/assembly/GCA_052112365.1/], and GCA_040947875.1 [https://www.ncbi.nlm.nih.gov/assembly/GCA_040947875.1/]. The raw mass spectrometry metabolomics data generated in this study have been deposited in the MassIVE database under accession code MSV000102692. All other data needed to evaluate the conclusions of the study are provided within the paper and its Supplementary Information. Source data are provided with this paper.

## Code availability

No original code was developed for this study.
