# Supplementary material for: The Caenorhabditis elegans microbiome impacts microsporidia infection through nutrient limitation and spore inactivation
Source: Nat Commun. 2026 Aug 26;17:10193. doi: 10.1038/s41467-026-77106-x (PMC13612480; doi:10.1038/s41467-026-77106-x)
Supplement: Supplementary file 1 [file 41467_2026_77106_MOESM1_ESM.pdf]

Supplementary Information

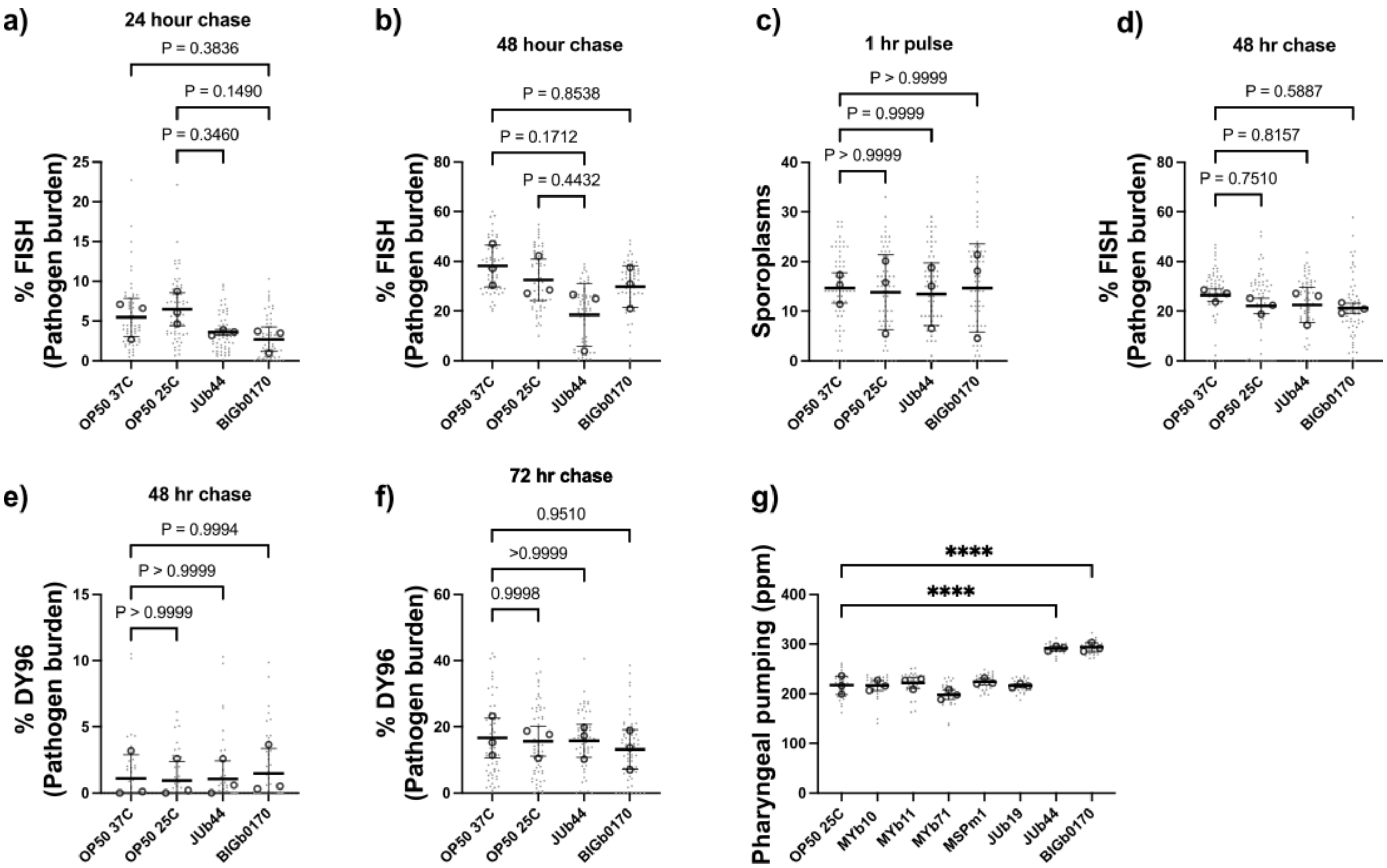

Supplementary Figure 1. Growth on *C. scophthalmum* JUb44 and *S. multivorum* BIGb0170 for 24 hours does not impact *N. parisii* infection but increases pharyngeal pumping.

(a-b) 72-hour old adults were pulse infected with *N. parisii* and *E. coli* OP50 for one hour and fixed at 24 hpi (a) or 48 hpi (b) and stained with FISH probes and DY96 and the amount of pathogen load was quantified. (c-f) Synchronized N2 L1 animals were grown on bacterial lawns for 24 hours and then pulse infected with *N. parisii* and *E. coli* OP50 for one hour and fixed at 1 hpi (c), 48 hpi (d, e), or 72 hpi (f). Samples were stained with FISH probes and DY96 and infection was quantified. (g) Quantification of pharyngeal pumps per minute after 72 hours of growth on the indicated bacteria. N = 3 biological replicates of 10 (g) or 15-20 (a-f) worms each. Mean ± SD represented by horizontal bars. P-values were determined via one-way ANOVA with Šidák's multiple comparisons test (a-f) or with Dunnett's multiple comparisons test (g). All tested conditions were compared to OP50 25 °C (g) or both OP50 37 °C and OP50 25 °C (a-f). Significance defined as **** $p < 0.0001$ and all p-values are included in the source data file.

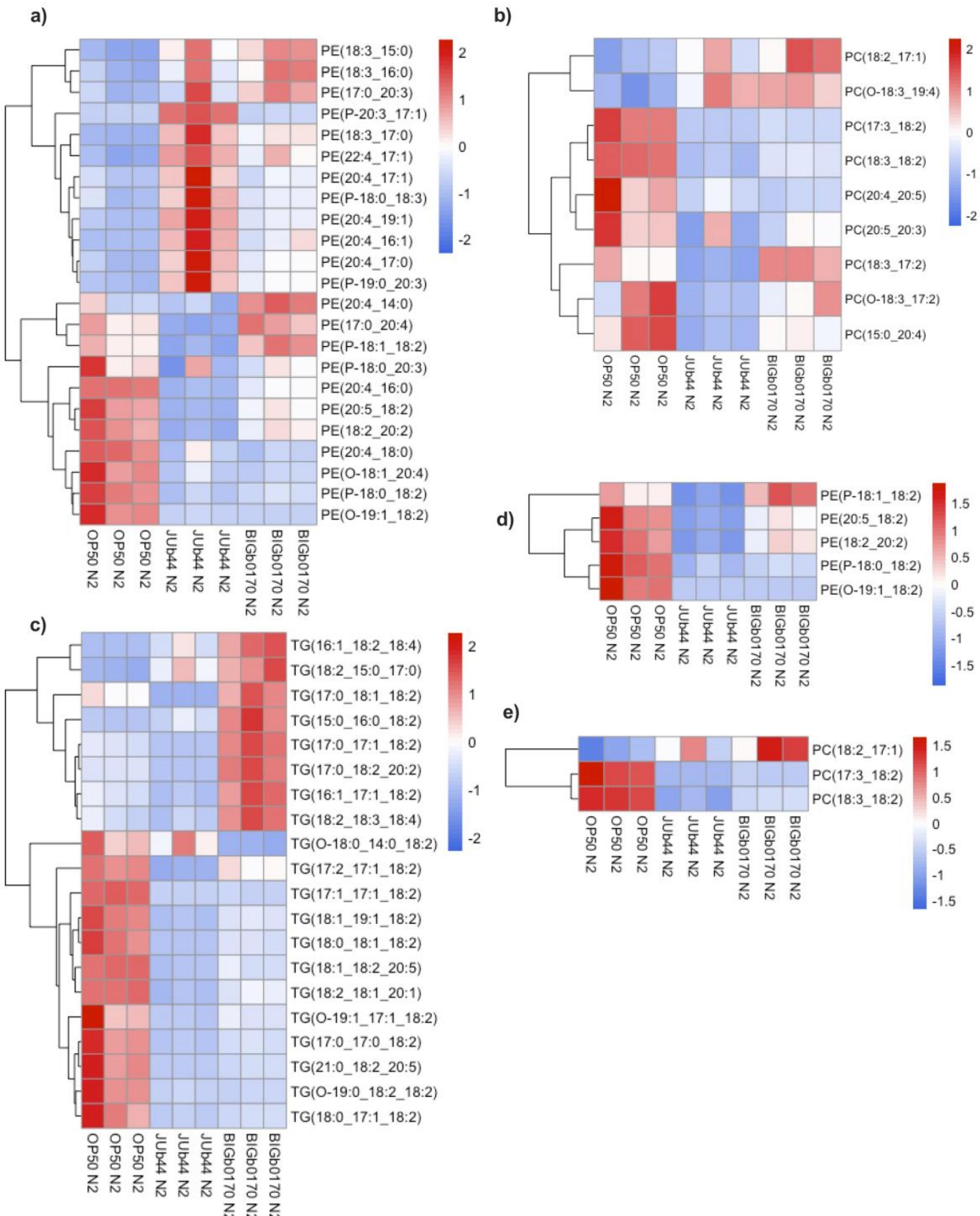

Supplementary Figure 2. Abundance of triglycerides (TG), phosphatidylcholines (PC), and phosphatidylethanolamines (PE) in N2 animals fed *E. coli* OP50*, C. scophthalmum* JUb44, or *S. multivorum* BIGb0170.

(a-b) Heat maps depicting the overall abundance of phosphatidylethanolamines (a) and phosphatidylcholines that contain at least one polyunsaturated fatty acid chain (b). (c-e) Heat maps depicting the abundance of 18:2 acyl chain containing triglycerides (c), phosphatidylethanolamines (d), and phosphatidylcholines (e). N = 3 biological replicates.

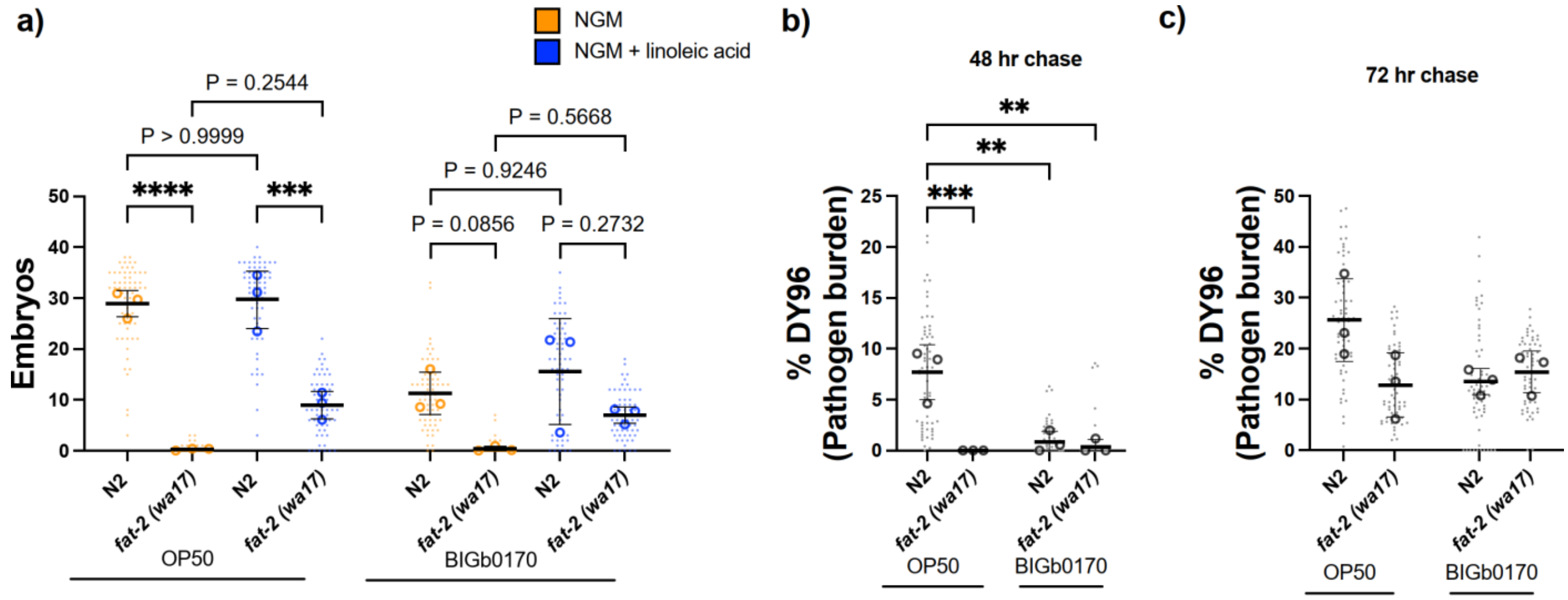

Supplementary Figure 3. Linoleic acid supplementation partially rescues embryo number reduction in *fat-2 (wa17)* mutants, and these mutants display reduced *N. parisii* infection burdens. (a) N2 or *fat-2 (wa17)* animals were grown in the presence (blue) or absence (orange) of linoleic acid for 72 hours. The number of embryos was quantified. The bacterial diet is denoted with a solid line below the X-axis. (b-c) 72-hour old adult N2 or *fat-2 (wa17)* animals were infected for one hour with *N. parisii* and fixed either 48 (b) or 72 (c) hours post-infection. The level of spores (b-c) was quantified. N = 3 biological replicates of 16-20 worms each. Mean ± SD represented by horizontal bars. P-values determined via one-way ANOVA with Tukey's multiple comparisons test. Comparisons were made between all possible pairs. Significance defined as ** p < 0.01, *** p < 0.001, **** p < 0.0001 and all p-values are included in the source data file.

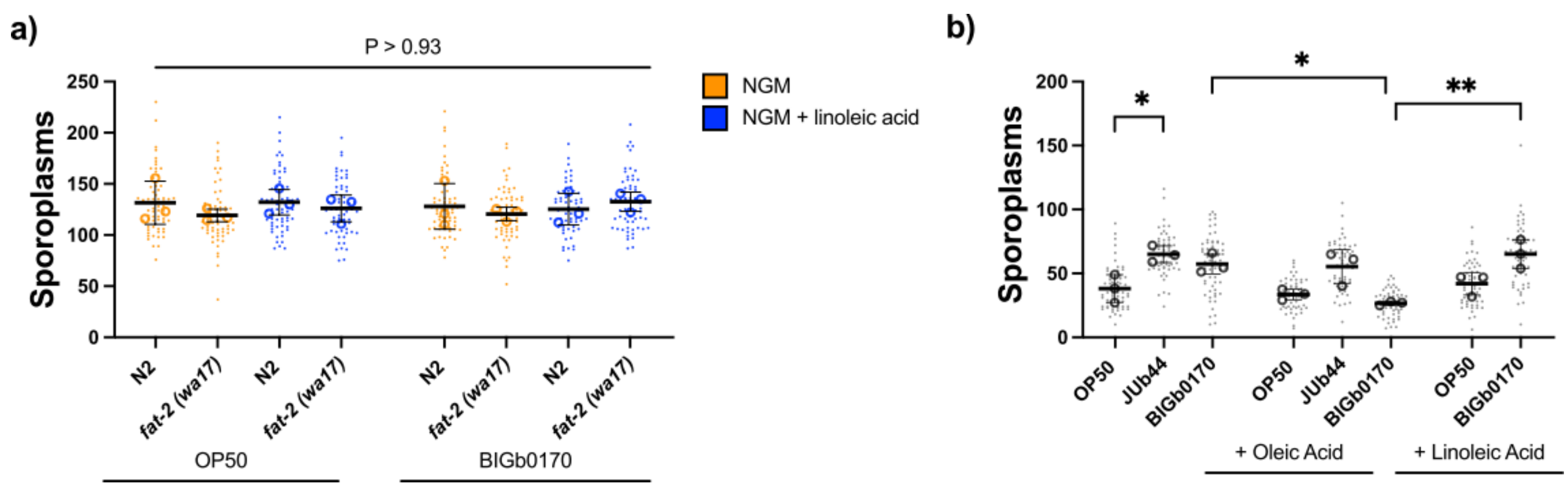

Supplementary Figure 4. Linoleic acid does not affect sporoplasm invasion and oleic acid supplementation reduces sporoplasm invasion on *S. multivorum* BIGb0170.

(a) N2 or *fat-2 (wa17)* animals were grown in the presence (blue) or absence (orange) of linoleic acid for 72 hours and infected for one hour. The number of sporoplasms was quantified. (b) N2 animals were grown on *E. coli* OP50, *C. scophthalmum* JUb44 or *S. multivorum* BIGb0170 in the presence or absence of oleic or linoleic acid for 72 hours and infected for one hour. The number of sporoplasms was quantified. The supplemented fatty acids are denoted with a solid line below the X-axis. N = 3 biological replicates of 20 worms each. Mean ± SD represented by horizontal bars. P-values determined via one-way ANOVA with Tukey's multiple comparisons test. Comparisons were made between all possible pairs. Significance defined as * $p < 0.05$, ** $p < 0.01$ and all p-values are included in the source data file.

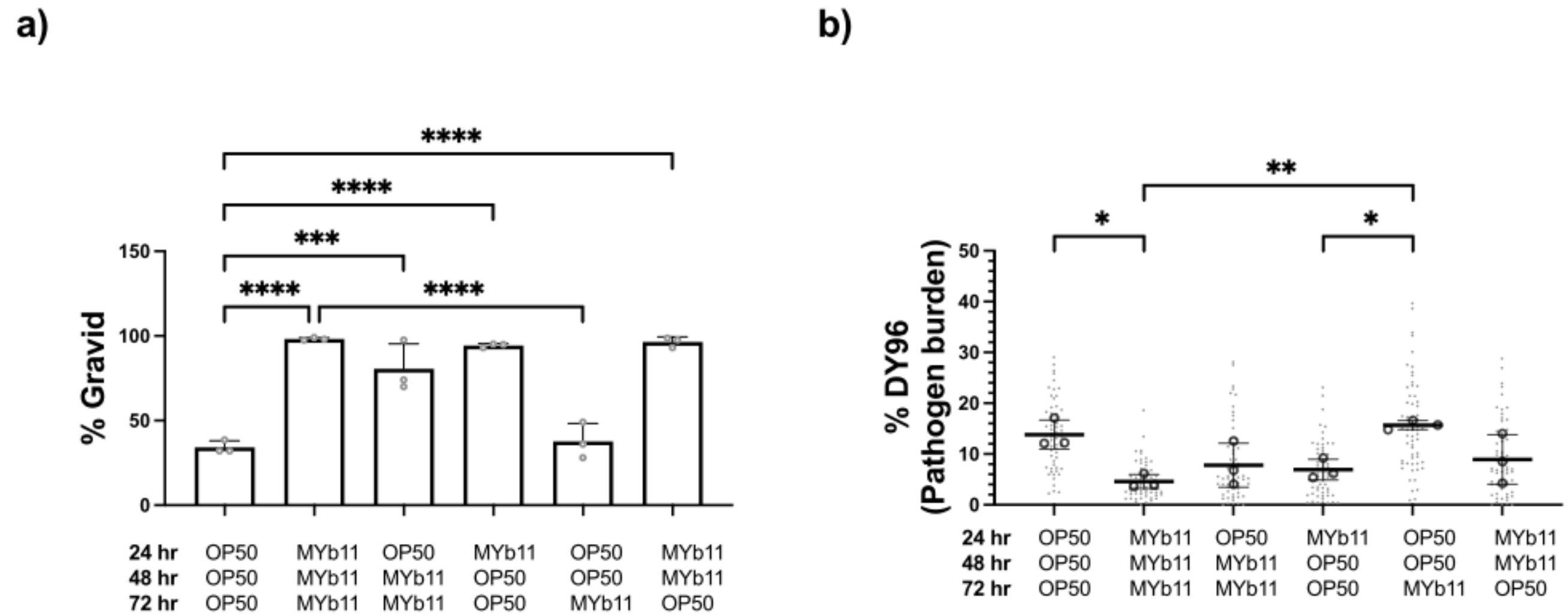

Supplementary Figure 5. Protection by MYb11 against *N. parisii* can occur during the first 48 hours of exposure.

(a-b) Animals were placed on diets of either *E. coli* OP50 or *P. lurida* MYb11 for varying amounts of time as indicated by the X-axis. Population fitness (a) and pathogen load (b) were quantified. N = 3 biological replicates of at least 20 worms each (a, b). Mean ± SD represented by horizontal bars. P-values determined via one-way ANOVA with Tukey's multiple comparisons test. Comparisons were made between all possible pairs. Significance defined as ** $p < 0.01$, *** $p < 0.001$, **** $p < 0.0001$ and all p-values are included in the source data file.

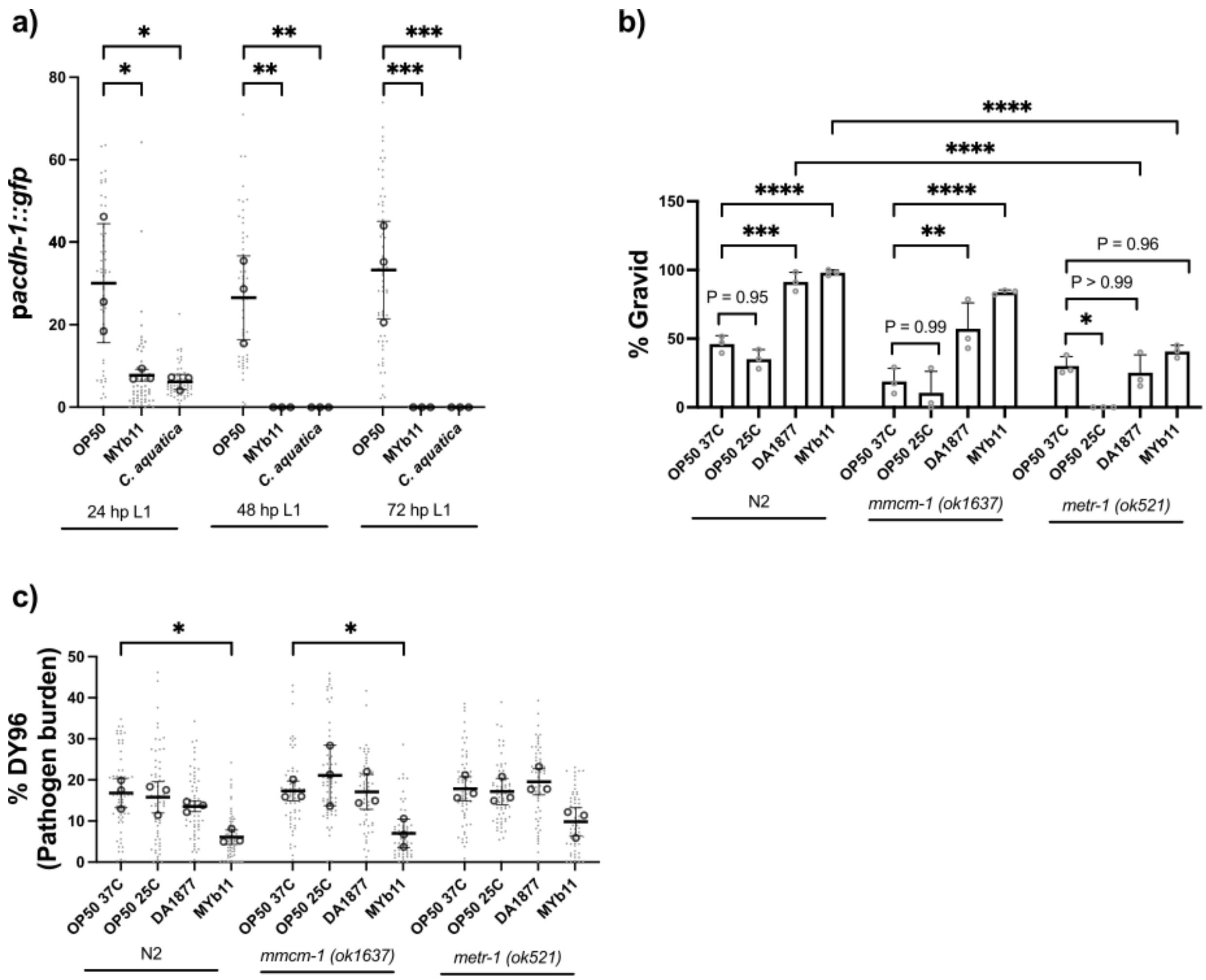

Supplementary Figure 6. *P. lurida* MYb11 provides resistance against *N. parisii* which is independent of vitamin B12 developmental acceleration.

(a) *acdh-1* expression levels were measured on various diets to assess the presence (GFP off) or absence (GFP on) of vitamin B12 in synchronized N2 animals every 24 hours. (b, c) Synchronized N2, *mmcm-1 (ok1637)* or *metr-1 (ok521)* L1s were pulse infected with *N. parisii* for 1 hour on *E. coli* OP50 prior to washing and splitting the populations onto individual seeded plates. 72 hours later, population fitness (b) and pathogen load (c) were quantified. N = 3 biological replicates of at least 20 (a, c) or 100 worms each (b). Mean ± SD represented by horizontal bars. P-values determined via one-way ANOVA with Tukey's multiple comparisons test (a, b) or with Šidák's multiple comparisons test (c). Comparisons were made between all possible pairs (a, b) or between each condition and the OP50 37 °C of the corresponding *C. elegans* strain (c). Significance defined as: * $p < 0.05$, ** $p < 0.01$, *** $p < 0.001$, **** $p < 0.0001$ and all p-values are included in the source data file.

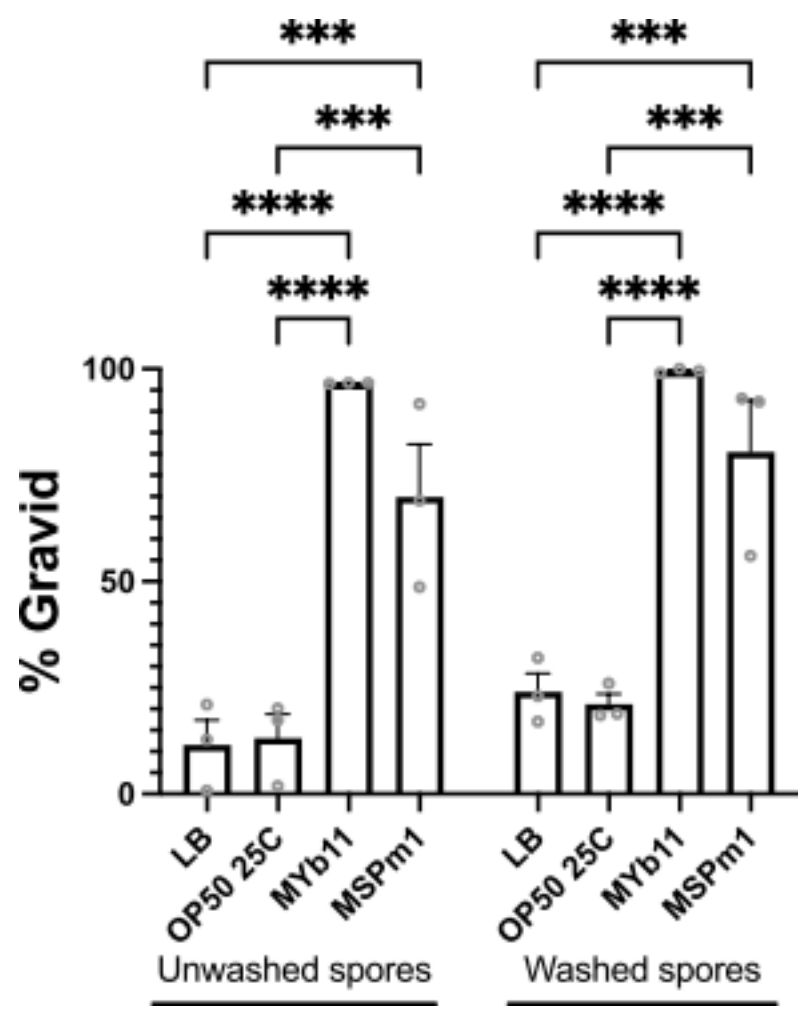

Supplementary Figure 7. *P. lurida* MYb11 and *P. mendocina* MSPm1 secrete molecules which directly act on *N. parisii* spores.

Spores incubated in supernatants denoted on the X-axis were washed to remove residual supernatants prior to L1 pulse infection**.** Population fitness was quantified. N = 3 biological replicates of 100 worms each. Mean ± SD represented by horizontal bars. P-values determined via one-way ANOVA with Tukey's multiple comparisons test, with a single pooled variance. Comparisons were made between all possible pairs. Significance defined as * p < 0.05, ** p < 0.01, *** p < 0.001, **** p < 0.0001 and all p-values are included in the source data file.

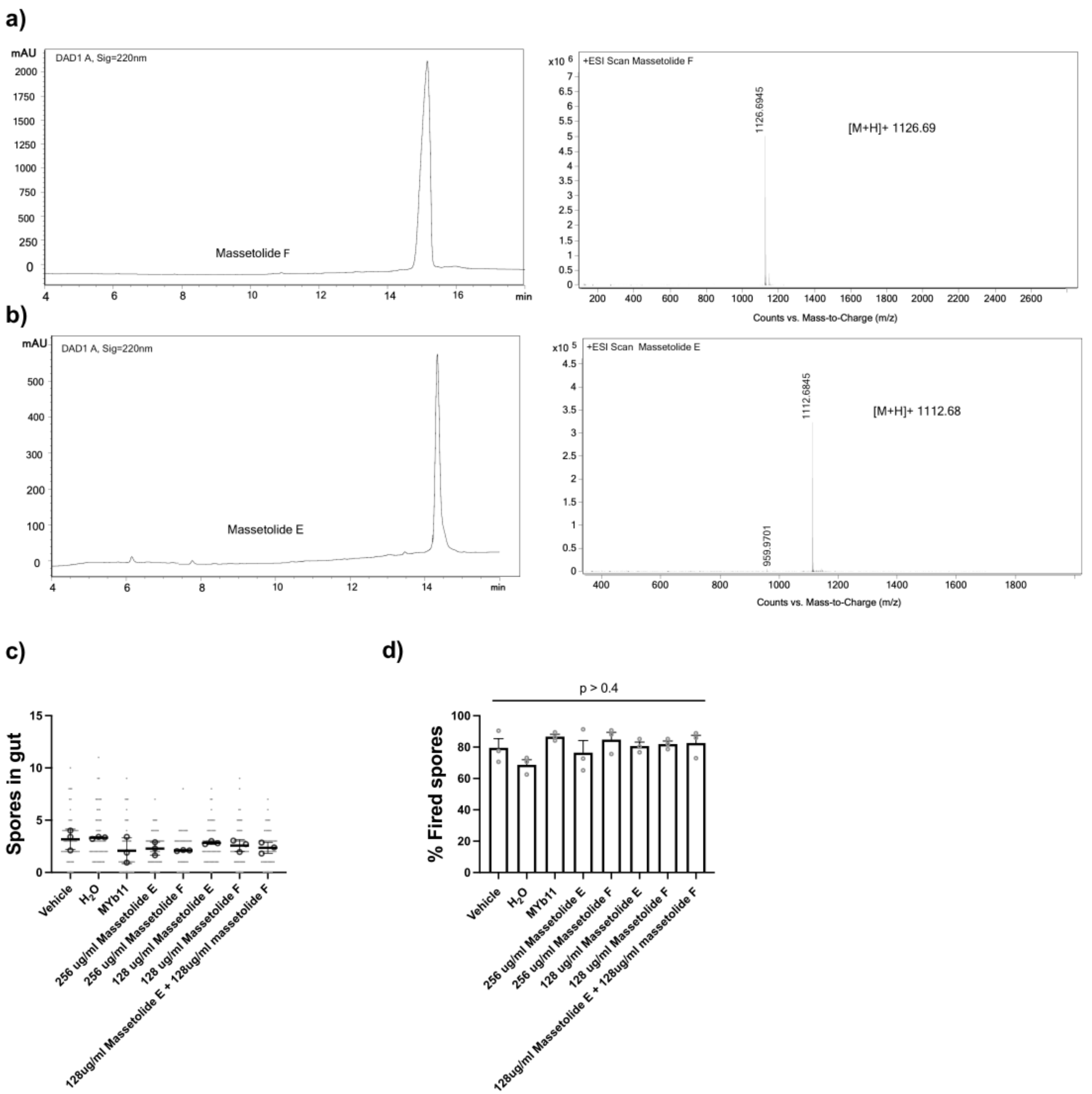

Supplementary Figure 8. Massetolides E and F and their impact on the number of spores and the fraction of fired spores after in vitro incubation.

(a-b) The HPLC chromatogram of massetolide F (a) and E (b) isolated from *P. lurida* MYb11 supernatant along with their high-resolution mass spectrometry (HR-MS) in positive ion mode. Massetolide F was identified by HR-MS as indicated by the peak at m/z 1126.69 [M+H]+ and massetolide E was identified by HR-MS as indicated by the peak at m/z 1112.68 [M+H]+**.** (c, d) Spores were incubated in either a vehicle control (0.5% DMSO), water, *P. lurida* MYb11 supernatant or massetolide E and/or F in various concentrations prior to L1 infection and the number of spores (c) and fraction of fired spores (d) were determined. N = 3 biological replicates of 20 worms each and at least 50 spores each (c, d). Mean ± SD

represented by horizontal bars. P-values determined via one-way ANOVA with Dunnett's multiple comparisons test, with a single pooled variance. Each test condition was compared to the vehicle and all p-values are included in the source data file.

a)

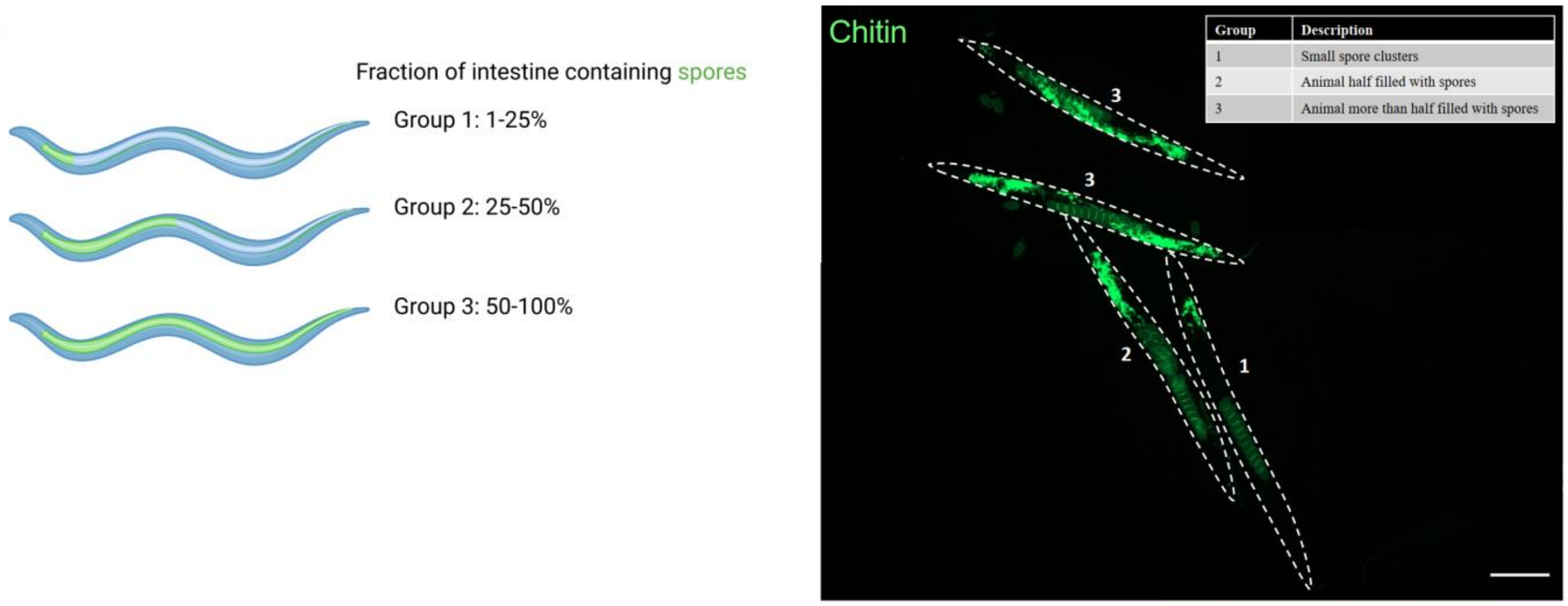

b)

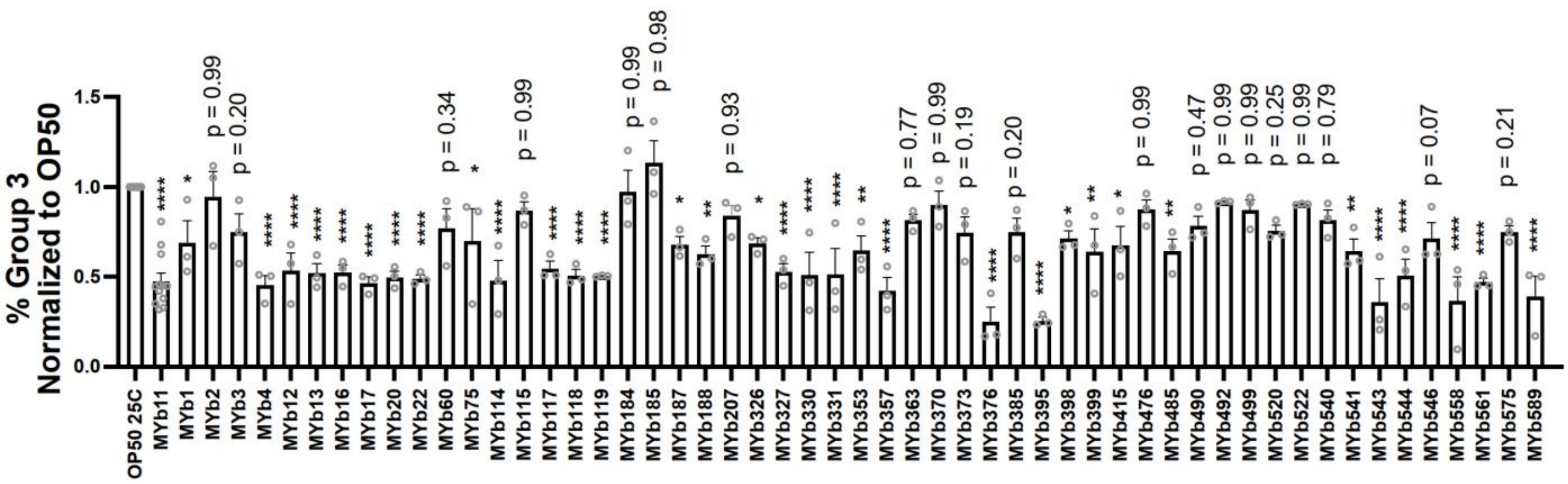

Supplementary Figure 9. Cultured media from *Pseudomonas* spp. reduce *N. parisii* spore infectivity.

(a) A schematic depicting the infection scale used to quantify pathogen burden (left) and a representative image of 72-hour old worms (outlined in dashed white lines) infected with *N. parisii* incubated in *P. lurida* MYb11 supernatant (right). Spores and embryos are stained with the chitin-binding dye DY96. The number next to each nematode indicates the group under which it would be categorized using this infection scale. Scale bar represents 200 μm. Schematic generated via BioRender.com. (b) The percentage of animals belonging to group 3 is normalized relative to animals infected with spores incubated in *E. coli* OP50 supernatant. The X-axis denotes the supernatants in which *N. parisii* spores were incubated. N = 3 biological replicates of at least 50 worms each. Mean ± SD represented by horizontal bars. P-values determined via one-way ANOVA with Dunnett's multiple comparisons test, with a single pooled variance. Each test condition was compared to the *E. coli* OP50 25 °C control condition. Significance defined as * $p < 0.1$, ** $p < 0.01$, **** $p < 0.0001$ and is relative to OP50 25 °C. All p-values are included in the source data file. Schematic was created in BioRender. Reinke, A. (2026) https://BioRender.com/51qi53j.

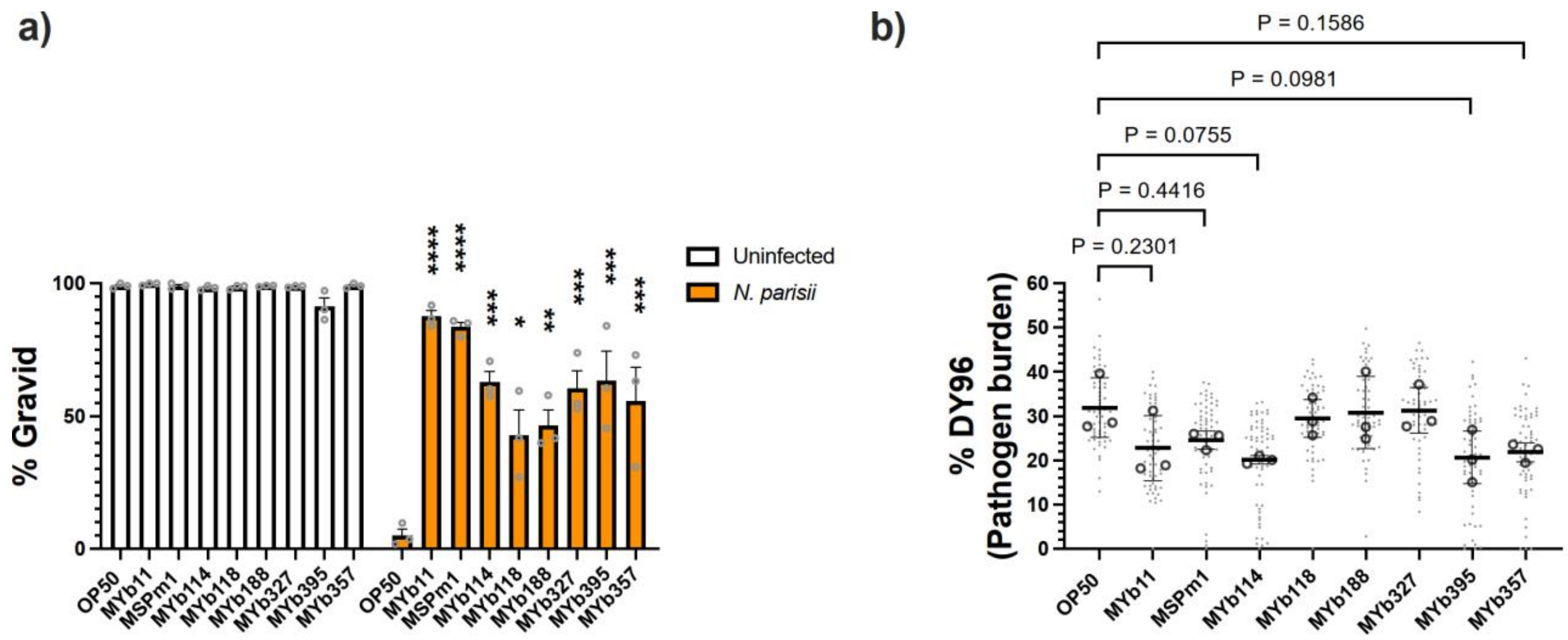

Supplementary Figure 10. Infected animals grown on lawns of *Pseudomonas* spp. display improved fitness. (a, b) Synchronized N2 L1 animals were continuously infected for 72 hours. Population fitness (a) and the pathogen burden (b) are displayed. N = 3 biological replicates of at least 100 (a) or 20 (b) worms each. Mean ± SD represented by horizontal bars. P-values determined via one-way ANOVA with Dunnett's multiple comparisons test, with a single pooled variance. Each test condition was compared to the respective control *E. coli* OP50 25 °C. Significance defined as * $p < 0.05$, ** $p < 0.01$, *** $p < 0.001$, **** $p < 0.0001$ and is relative to OP50 25 °C of the corresponding condition. All p-values are included in the source data file.

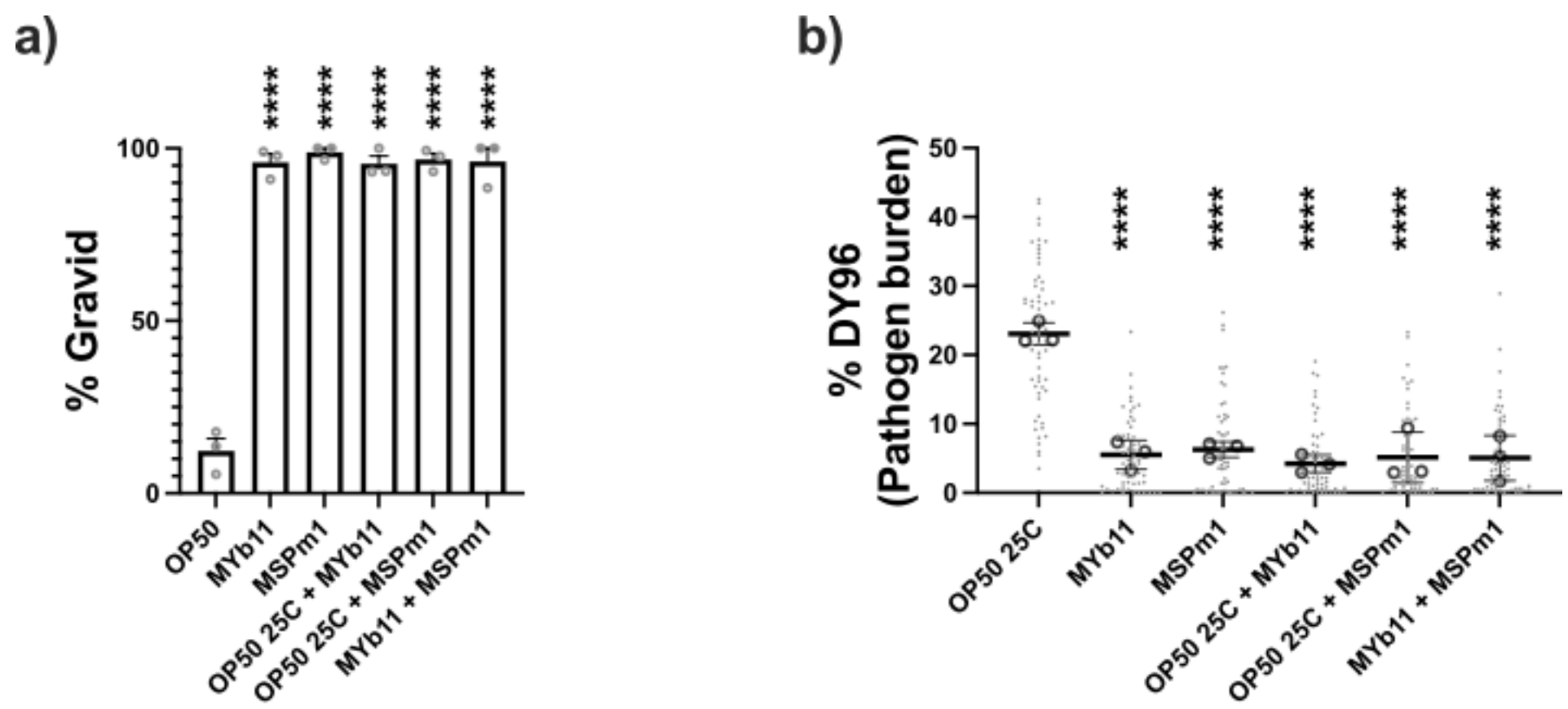

Supplementary Figure 11. Infected animals grown on a combination of *P. lurida* MYb11 and *P. mendocina* MSPm1 results in decreased pathogen burden and improved host fitness.

(a, b) Synchronized N2 L1 animals were continuously infected on lawns of bacteria indicated on the X-axis for 72 hours. Two bacterial species indicate a combination of the two in a 1:1 ratio (90 µl each) used to seed the NGM plates. The fraction of gravid adults (a) and the pathogen burden (b) are displayed. N = 3 biological replicates of at least 100 (a) or 20 (b) worms each. Mean ± SD represented by horizontal bars. P-values determined via one-way ANOVA with Dunnett's multiple comparisons test, with a single pooled variance. Each test condition was compared to *E. coli* OP50 25 °C. Significance defined as **** p < 0.0001 and is relative to OP50 25 °C of the corresponding condition. All p-values are included in the source data file.

Supplementary Table 1. Diversity of natural compounds produced by *Pseudomonas* spp.

The results from antiSMASH analysis are indicated under "Most similar known cluster". A percentage in between brackets indicates the similarity score to the clusters. The indication of two different percentage scores for the same predicted cluster is representative of two separate clusters at different genomic regions. Similarities of 50% or higher are bolded. Strains with tested inhibitory activity against *N. parisii* are bolded.

Strain	Most similar known cluster (similarity %)
**MSPm1**	APE Vf (45%); Pf-5 Pyoverdine (14%); Corynecin III/Corynecin I/Corynecin II (13%); Lankacidin C (13%)
**MYb11**	**Viscosin (100%)**; **Tolaasin I/Tolaasin F (60%);** APE Vf (45%); Ambactin (25%); Fengycin (13%); Lankacidin C (13%); Pf-5 Pyoverdine (9%); Lipopolysaccharide (5%);
**MYb1**	**Viscosin (100%); Viscosinamide A/ Pseudodesmin A (50%)**; APE vf (45%); Ambactin (25%); Fengycin (13%); Lankacidin C (13%); Pyoverdine pf-5 (9%); Lipopolysaccharide (5%)
MYb2	**Hydrogen cyanide (100%)**; **Lokisin (71%)**; Ape Vf (40%), Fragin (37%); Pf-5 pyoverdine (20%); Pf-5 pyoverdine (17%); Fengycin (13%); Lankacidin C (13%)
MYb3	**Hydrogen cyanide (100%)**; **Lokisin (71%)**; Ape Vf (40%), Fragin (37%); Pf-5 pyoverdine (20%); Pf-5 pyoverdine (17%); Fengycin (13%); Lankacidin C (13%)
**MYb12**	**Viscosin (100%); Viscosinamide A/ Pseudodesmin A (50%)**; APE vf (45%); Fragin (25%/13%); Variobactin A/Variobactin B (21%); Pyoverdine SMX-1 (16%); Lankacidin C (13%); Pyoverdine pf-5 (9%); Lipopolysaccharide (5%)
**MYb13**	**Viscosin (100%)**; **Tolaasin I/F (70%)**; APE Vf (45%); Puwainaphycin F/Minutissamide A/Minutissamide B/Minutissamide C/Minutissamide D (30%); Azotobactin D (25%); Fragin (25%); Pyoverdine SMX-1 (16%); Lankacidin C (13%); Fengycin (13%); Pf-5 pyoverdine (9%); Lipopolysaccharide (5%)
**MYb16**	**Viscosin (100%)**; **Tolaasin I/F (70%)**; APE Vf (45%); Fragin (25%); Variobactin A/B (21%); Pyoverdine SMX-1 (16%); Lankacidin C (13%); Fengycin (13%); Pf-5 pyoverdine (9%); Lipopolysaccharide (5%)
**MYb17**	**Viscosin (100%)**; **Viscosinamide A/ Pseudodesmin A (50%)**; APE Vf (45%); Fragin (25%); Variobactin A/B (21%); Pyoverdine SMX-1 (16%); Lankacidin C (13%); Fengycin (13%); Pf-5 pyoverdine (9%); Lipopolysaccharide (5%)
**MYb22**	**Viscosin (100%)**; **Tolaasin I/F (60%)**; APE Vf (45%); Fragin (25%); Variobactin A/F (21%); Pyoverdine SMX-1 (16%); Lankacidin C (13%); Fengycin (13%);
MYb60	APE Vf (40%); Fragin (25%); Lankacidin C (13%); Pf-5 pyoverdine (10%/9%); 12-epi-hapalindole C isonitrile/12-epi-hapalindole E/12-epi-fischerindole U isonitrile/fischerindole L/12-epi-fischerindole I isonitrile/welwitindolinone A isonitrile/welwitindolinone B isothiocyanate/welwitindolinone C isothiocyanate/N-methylwelwitindolinone C isothiocyanate/N-methylwelwitinsolinone C isonitrile/3-epi-welwitindolinone B isothiocyanate/3-(Z-2'-isocyanoethenyl)-indole (6%); Lipopolysaccharide (5%); Pf-5 pyoverdine (3%)
**MYb75**	APE Vf (40%); Fragin (25%); Lankacidin C (13%); Pf-5 pyoverdine (10%/9%); Lipopolysaccharide (5%)
**MYb114**	APE Vf (40%); Pyoverdine SMX-1 (38%); Lokisin (35%); Ambactin (25%); Lankacidin C (13%); Fengycin (13%); Pf-5 Pyoverdine (10%). Lipopolysaccharide (5%); Deoxyhangtaimycin (2%)
MYb115	APE Vf (40%); Cupriachelin (35%); Fengycin (13%); Lankacidin C (13%); Pf-5 pyoverdine (11%); Thaxteramide (7%); MA026 (2%)
**MYb118**	APE Vf (40%); Ambactin (25%); Pf-5 Pyoverdine (20%/27%); Lankacidin C (13%); fengycin (13%)
MYb184	Viscosin (40%); APE Vf (40%); Pyoverdine SMX-1 (38%); Pyochelin (28%); Ambactin (25%); MA026 (14%); Fengycin (13%); Lankacidin C (13%); Pyoverdine SMX-1 (12%); Lipopolysaccharide (5%); Armeniaspirol/Armeniaspirol B/Armeniaspirol C (5%)
**MYb187**	Aryl Polyenes (44%); Lankacidin C (13%); Pf-5 Pyoverdine (8%); Pf-5 Pyoverdine (4%)
**MYb188**	Aryl Polyenes (44%); Lankacidin C (13%); Pf-5 Pyoverdine (8%/4%)
**MYb327**	APE Vf (40%); Pf-5 Pyoverdine (21%/22%); Fengycin (13%); Lagriene (6%); Glycinocin A (4%)
**MYb357**	APE Vf (40%/40%); Fengycin (13%); Lankacidin C (13%); Pf-5 Pyoverdine (11%)
**MYb395**	**Viscosin (100%) Viscosinamide A/ Pseudodesmin A (50%)**; APE Vf (40%); Syringolin A (28%); Pyochelin (26%);Fragin (25%) **;** Fengycin (13%); Lankacidin C (13%); Pf-5 Pyoverdine (9%); lipopolysaccharide (5%)

Supplementary Table 2. List of *C. elegans* strains used in this study

Strain	Source	Genotype	Description
**N2**	Caenorhabditis Genetics Center (CGC)	N2	Wild-type, Bristol strain.
**RB755**	Caenorhabditis Genetics Center (CGC)	*metr-1 (ok521) II*	Deletion.
**RB1434**	Caenorhabditis Genetics Center (CGC)	*mmcm-1 (ok1637) III*	Deletion.
**VL749**	Caenorhabditis Genetics Center (CGC)	P*acdh-1::gfp*	GFP on in absence of vitamin B12.
**BX26**	Caenorhabditis Genetics Center (CGC)	*fat-2 (wa17) IV*	No delta 12 fatty acid desaturase activity.

Supplementary Table 3. Spore doses used in this study

The varying doses of spores (as defined in methods) are listed. Plate concentration refers to the number of spores occupied per cm^2 on a 6-cm NGM plate. The total number of spores present on a single assay plate are listed for the various doses in millions of spores.

**Species**	Dose	Plate concentration (spores cm^{-2})	Total spores on assay plate (Millions)
N. parisii* *(ERTm1)	Very low	2,495	0.024
	Low	74,835	0.72
	Medium	374,175	3.6
	High	4,781,125	46

Supplementary Table 4. Analysis of data normality using ANOVA residuals

Figure, panel	Test name	Statistics	P value	Passed normality test (<0.05)?	P-value
1C	D'Agostino-Pearson omnibus (K2)	6.934	0.0312	No	*
	Anderson-Darling (A2*)	0.7028	0.0589	Yes	ns
	Shapiro-Wilk (W)	0.9196	0.0387	No	*
	Kolmogorov-Smirnov (distance)	0.1275	0.1	Yes	ns
1E	D'Agostino-Pearson omnibus (K2)	1.41	0.4942	Yes	ns
	Anderson-Darling (A2*)	0.7917	0.0307	No	*
	Shapiro-Wilk (W)	0.8946	0.0786	Yes	ns
	Kolmogorov-Smirnov (distance)	0.2359	0.0243	No	*
4F	D'Agostino-Pearson omnibus (K2)	1.638	0.4408	Yes	ns
	Anderson-Darling (A2*)	0.4766	0.1929	Yes	ns
	Shapiro-Wilk (W)	0.9241	0.3217	Yes	ns
	Kolmogorov-Smirnov (distance)	0.2473	0.0411	No	*
S1E	D'Agostino-Pearson omnibus (K2)	4.2	0.1225	Yes	ns
	Anderson-Darling (A2*)	1.443	0.0005	No	***
	Shapiro-Wilk (W)	0.7394	0.0021	No	**
	Kolmogorov-Smirnov (distance)	0.299	0.004	No	**
S3A	D'Agostino-Pearson omnibus (K2)	10.25	0.0059	No	**
	Anderson-Darling (A2*)	0.7087	0.0559	Yes	ns
	Shapiro-Wilk (W)	0.9082	0.0322	No	*
	Kolmogorov-Smirnov (distance)	0.1725	0.0629	Yes	ns
S3B	D'Agostino-Pearson omnibus (K2)	6.12	0.0469	No	*
	Anderson-Darling (A2*)	0.5227	0.1449	Yes	ns
	Shapiro-Wilk (W)	0.8995	0.1564	Yes	ns
	Kolmogorov-Smirnov (distance)	0.2111	0.1	Yes	ns
S5A	D'Agostino-Pearson omnibus (K2)	6.468	0.0394	No	*
	Anderson-Darling (A2*)	0.7919	0.0323	No	*
	Shapiro-Wilk (W)	0.8999	0.0571	Yes	ns
	Kolmogorov-Smirnov (distance)	0.2105	0.0339	No	*
S6A	D'Agostino-Pearson omnibus (K2)	4.53	0.1038	Yes	ns
	Anderson-Darling (A2*)	2.586	<0.0001	No	****
	Shapiro-Wilk (W)	0.8159	0.0003	No	***
	Kolmogorov-Smirnov (distance)	0.2556	<0.0001	No	****
S6B	D'Agostino-Pearson omnibus (K2)	6.206	0.0449	No	*
	Anderson-Darling (A2*)	0.7195	0.0552	Yes	ns
	Shapiro-Wilk (W)	0.9437	0.0662	Yes	ns
	Kolmogorov-Smirnov (distance)	0.1612	0.0188	No	*
S7	D'Agostino-Pearson omnibus (K2)	2.959	0.2277	Yes	ns
	Anderson-Darling (A2*)	0.6852	0.0641	Yes	ns
	Shapiro-Wilk (W)	0.943	0.1903	Yes	ns
	Kolmogorov-Smirnov (distance)	0.1895	0.0258	No	*
S9B	D'Agostino-Pearson omnibus (K2)	1.288	0.5251	Yes	ns
	Anderson-Darling (A2*)	0.8452	0.0291	No	*
	Shapiro-Wilk (W)	0.9909	0.3015	Yes	ns
	Kolmogorov-Smirnov (distance)	0.07907	0.0073	No	**
S10A_infected	D'Agostino-Pearson omnibus (K2)	0.4438	0.801	Yes	ns
	Anderson-Darling (A2*)	0.4489	0.2571	Yes	ns
	Shapiro-Wilk (W)	0.9661	0.5034	Yes	ns
	Kolmogorov-Smirnov (distance)	0.1232	0.1	Yes	ns
S10B	D'Agostino-Pearson omnibus (K2)	3.024	0.2204	Yes	ns
	Anderson-Darling (A2*)	0.8231	0.0291	No	*
	Shapiro-Wilk (W)	0.9138	0.0281	No	*
	Kolmogorov-Smirnov (distance)	0.1465	0.1	Yes	ns
All other panels pass all 4 of the normality tests.					
