# Supplementary material for: The Caenorhabditis elegans microbiome impacts microsporidia infection through nutrient limitation and spore inactivation
Source: Nat Commun. 2026 Aug 26;17:10193. doi: 10.1038/s41467-026-77106-x (PMC13612480; doi:10.1038/s41467-026-77106-x)
Supplement: Supplementary file 2 [file 41467_2026_77106_MOESM2_ESM.pdf]

File Name: Supplementary Data 1

Description: Polar metabolomic data.

File Name: Supplementary Data 2

Description: Lipidomic metabolomic data.
