# Supplementary material for: The Caenorhabditis elegans microbiome impacts microsporidia infection through nutrient limitation and spore inactivation
Source: Nat Commun. 2026 Aug 26;17:10193. doi: 10.1038/s41467-026-77106-x (PMC13612480; doi:10.1038/s41467-026-77106-x)
Supplement: Supplementary file 3 [file 41467_2026_77106_MOESM5_ESM.pdf]

nature portfolio

Corresponding author(s): Aaron Reinke
Last updated by author(s): Jul 21, 2026

Reporting Summary

Nature Portfolio wishes to improve the reproducibility of the work that we publish. This form provides structure for consistency and transparency in reporting. For further information on Nature Portfolio policies, see our Editorial Policies and the Editorial Policy Checklist.

Statistics

For all statistical analyses, confirm that the following items are present in the figure legend, table legend, main text, or Methods section.

n/a	Confirmed	
[ ]	[x]	The exact sample size (*n*) for each experimental group/condition, given as a discrete number and unit of measurement
[ ]	[x]	A statement on whether measurements were taken from distinct samples or whether the same sample was measured repeatedly
[ ]	[x]	The statistical test(s) used AND whether they are one- or two-sided<br>*Only common tests should be described solely by name; describe more complex techniques in the Methods section.*
[x]	[ ]	A description of all covariates tested
[ ]	[x]	A description of any assumptions or corrections, such as tests of normality and adjustment for multiple comparisons
[ ]	[x]	A full description of the statistical parameters including central tendency (e.g. means) or other basic estimates (e.g. regression coefficient) AND variation (e.g. standard deviation) or associated estimates of uncertainty (e.g. confidence intervals)
[ ]	[x]	For null hypothesis testing, the test statistic (e.g. *F*, *t*, *r*) with confidence intervals, effect sizes, degrees of freedom and *P* value noted<br>*Give P values as exact values whenever suitable.*
[x]	[ ]	For Bayesian analysis, information on the choice of priors and Markov chain Monte Carlo settings
[x]	[ ]	For hierarchical and complex designs, identification of the appropriate level for tests and full reporting of outcomes
[x]	[ ]	Estimates of effect sizes (e.g. Cohen's *d*, Pearson's *r*), indicating how they were calculated

Our web collection on statistics for biologists contains articles on many of the points above.

Software and code

Policy information about availability of computer code

Data collection	Zeiss ZEN 2.3,
Data analysis	All statistical analyses were conducting using GraphPad prism v10.0. Image analysis was conducted with FIJI version v2.1.0. BioRender was used to create schematic figures. LipidSearch v5.0 was used for lipid annotation and used to generate a mass list for peak picking and integration of experimental replicates in Compound Discoverer v3.3. Phylogenetic trees were constructed using ModelTest-NG v0.1.7 andRAxML-NG v. 1.2.1 and the tree was visualized using R packages ape v5.7.1, ggtree v3.10.0, ggplot2 v3.4.4, ggnewscale v0.4.9, ggtreeExtra v1.12.0, ggstar v1.0.4, and RColorBrewer v1.1.3.

For manuscripts utilizing custom algorithms or software that are central to the research but not yet described in published literature, software must be made available to editors and reviewers. We strongly encourage code deposition in a community repository (e.g. GitHub). See the Nature Portfolio guidelines for submitting code & software for further information.

Data

Policy information about availability of data

All manuscripts must include a data availability statement. This statement should provide the following information, where applicable:

- Accession codes, unique identifiers, or web links for publicly available datasets
- A description of any restrictions on data availability
- For clinical datasets or third party data, please ensure that the statement adheres to our policy

Sequences of rpoD generated in this study were deposited in NCBI with the accession numbers PP861230- PP861277 and genome assemblies for sequenced

Pseudomonas strains were deposited in NCBI with the accession numbers GCA_040438915.1, GCA_040438925.1, GCA_040438935.1, GCA_040438945.1, and GCA_040947875.1. The raw mass spectrometry metabolomics data generated in this study have been deposited in the MassIVE database under accession code MSV000102692. All other data needed to evaluate the conclusions in the paper are present in the paper, Supplementary Materials, and Source Data file.

Research involving human participants, their data, or biological material

Policy information about studies with human participants or human data. See also policy information about sex, gender (identity/presentation), and sexual orientation and race, ethnicity and racism.

Reporting on sex and gender	N/A
Reporting on race, ethnicity, or other socially relevant groupings	N/A
Population characteristics	N/A
Recruitment	N/A
Ethics oversight	N/A

Note that full information on the approval of the study protocol must also be provided in the manuscript.

Field-specific reporting

Please select the one below that is the best fit for your research. If you are not sure, read the appropriate sections before making your selection.

- [x] Life sciences
- [ ] Behavioural & social sciences
- [ ] Ecological, evolutionary & environmental sciences

For a reference copy of the document with all sections, see nature.com/documents/nr-reporting-summary-flat.pdf

Life sciences study design

All studies must disclose on these points even when the disclosure is negative.

Sample size	For all quantified data where statistics was performed n = 3 biological replicates. The number of replicates and animals analyzed for each replicate were chosen based on ours and others previous publications using similar assays. For C. elegans experiments, each biological replicate is made up of 10-100 individual animals depending on the experiment, which are sample sizes used in previous studies (Balla et al, Nature Microbiology 2016, and Willis et al., Science advances 2021).
Data exclusions	No data was excluded from the analysis of any experiments.
Replication	For almost all experiments, replication of experiments was successful, with at least three biological replicates for each experiment. The one exception were testing fractions of P. lurida MYb11 and P. mendocina MSPm1, for which only one or two replicates was performed.
Randomization	For all experiments, animals were taken from a large population of identical individuals, and randomly assorted into different treatment conditions. For infection experiments with spores, aliquots were taken from a pool and randomly distributed into different sample groups.
Blinding	Blinding was not performed for most experiments in this study. For microscopy-based assays, blinding was not necessary as clearly defined scoring criteria was used to manually score various phenotypes. The one exception is figure 4h, where counting differences in spores could be biased by knowing the identity of the samples, so all of these samples were blinded prior to quantification.

Reporting for specific materials, systems and methods

We require information from authors about some types of materials, experimental systems and methods used in many studies. Here, indicate whether each material, system or method listed is relevant to your study. If you are not sure if a list item applies to your research, read the appropriate section before selecting a response.

Materials & experimental systems

n/a	Involved in the study	
x		Antibodies
x		Eukaryotic cell lines
x		Palaeontology and archaeology
	x	Animals and other organisms
x		Clinical data
x		Dual use research of concern
x		Plants

Methods

n/a	Involved in the study	
x		ChIP-seq
x		Flow cytometry
x		MRI-based neuroimaging

Animals and other research organisms

Policy information about studies involving animals; ARRIVE guidelines recommended for reporting animal research, and Sex and Gender in Research

Laboratory animals	Hermaphrodite Caenorhabditis elegans animals were used for all experiments. All experiments were done starting with a population of F1 L1 animals, taken from mixed populations of P0s. The strain N2 was used for all experiments, except where indicated and all strains are listed in Supplementary Table 4. The stage of the animals is provided in the figure legend for each experiment.
Wild animals	This study did not involve wild animals.
Reporting on sex	All animals used in the study were hermaphrodites.
Field-collected samples	This study did not involve samples collected from the field.
Ethics oversight	No ethical approval was required as these experiments were performed on laboratory nematode invertebrates.

Note that full information on the approval of the study protocol must also be provided in the manuscript.

Plants

Seed stocks	n/a
Novel plant genotypes	n/a
Authentication	n/a
