# Supplementary material for: The Caenorhabditis elegans microbiome impacts microsporidia infection through nutrient limitation and spore inactivation
Source: Nat Commun. 2026 Aug 26;17:10193. doi: 10.1038/s41467-026-77106-x (PMC13612480; doi:10.1038/s41467-026-77106-x)
Supplement: Supplementary file 4 [file 41467_2026_77106_MOESM6_ESM.pdf]

nature portfolio

Peer Review File

The Caenorhabditis elegans microbiome impacts microsporidia infection through nutrient limitation and spore inactivation

Corresponding Author: Dr Aaron Reinke

This file contains all reviewer reports in order by version, followed by all author rebuttals in order by version.

Version 0:

Reviewer comments:

Reviewer #1

(Remarks to the Author)
Review for "The Caenorhabditis elegans bacterial microbiome influences microsporidia infection through nutrient limitation and inhibiting parasite invasion," El Jarkass et al.
Remarks to the authors
In the manuscript titled "The Caenorhabditis elegans bacterial microbiome influences microsporidia infection through nutrient limitation and inhibiting parasite invasion," El Jarkass et al. analyzed the antimicrosporidial properties of various bacteria in the natural microbiome of C. elegans. This thorough study identifies several bacterial species that are detrimental to microsporidia, either directly or indirectly, and elucidates mechanisms by which bacteria can enhance host survival during infection. The study provides detailed characterization of different infection phases, employs diverse approaches, and uncovers new mechanisms that limit microsporidia infection or damage spores.
Specific comments and questions:
1. Several figures (e.g., 1b-e and 3b-d) are not fully visible, and the data and/or statistical results are missing. This issue also exists in the bioRxiv preprint.
2. The rationale and setup for the experiment tracking N. parisii inhibition by Pseudomonas over multiple generations are unclear. What are the potential outcomes? How do authors ensure they transfer infected animals? Do they use an IPR reporter? Does the age of plates affect the results due to bacterial die-off?
3. The narrative needs improvement. The rationale for focusing on specific bacterial strains is unclear. For example, the authors initially observed spore reduction with seven bacterial strains but followed up on two.
4. The increased sporoplasm number in animals fed with BIGb0170 and JUb44 is attributed to increased pharyngeal pumping. Do other bacterial species that do not cause increased colonization have normal or decreased pumping rates?
5. Does the growth rate of C. elegans on different bacterial food sources affect observed microsporidia infection levels?
6. Does mixing spores with OP50 instead of specific microbiome species during infection alter the results?
7. While lipidomics is part of metabolomics, "metabolomics" often describes hydrophilic molecule analysis. Consider using "lipidomics" to avoid confusion.
8. In Fig. 2d, blue dots for fat-2 on NGM (and for BIGb0170) are not visible.
9. Enhanced fitness and resistance to infection do not always correlate (e.g., vitamin B12 treatment). Yet, spores are described as less infective based on increased percentage of gravid animals. This is contradictory; more cautious wording is needed.
10. One-way ANOVA was used for all assays. Do all datasets have a normal distribution?
11. The legend for Fig. 6c states three replicates, but the graph suggests one (no bars; if SD values are too small, explain).
12. The authors adjust the optical density of bacteria prior to seeding the plates. However, they additionally incubate the plates and then store them at lower temperatures. These treatments likely affect different bacterial species differently (e.g., some might grow faster after seeding, while others might die off more quickly at the storage temperatures). Therefore, it is difficult to imagine that bacterial density remains consistent across all tested species at the time of the experiments. Could this variability affect the results shown in this study?
13. The authors refer to the microbiome even when some bacterial species seem to be nutritionally insufficient and therefore indirectly suppress pathogen development. It would be useful to perform additional experiments to determine if the role of some bacteria is purely nutritional and not required for colonizing the intestine of C. elegans.
14. In lines 410-411, the claim that "7% of bacteria associated with C. elegans activate this immune response" seems

incorrect. The referenced study (Gonzalez and Felix) found that 5/71 bacterial species induced IPR reporter expression stronger than OP50 in the presence of the Orsay virus, not necessarily in the absence of the pathogen.
15. Why were 11 instead of 12 CeMbio strains tested in the initial screen?
16. Inconsistent naming: BIGb0170 is referred to as S. multivorum and S. scopthalmum.
17. Consider referencing additional studies on microsporidia infections in C. elegans (e.g., PMIDs 24945527, 35544001, 38702921) and add a reference to line 436.
18. Consider rephrasing "more gravid" as it is a binary condition.

Reviewer #2

(Remarks to the Author)
The manuscript by El Jarkass et al. reports that feeding C. elegans different bacteria affects their susceptibility to infection with microsporidia. Building evidence indicates that host-associated bacteria play a central role for the susceptibility of animals, including humans, to infection by diverse pathogens, and C. elegans is an excellent model system to investigate the underlying mechanisms, as the authors point out in the introduction. However, the manuscript falls short of taking advantage of the opportunities this model system offers for mechanistic study.

Comments on the Results section:
Many of the results seem ambiguous and confusing, which is problematic for their interpretation given that changing bacterial diets can affect host physiology in very many different ways, directly and indirectly. For example, Bigb0170 seems to have more 18:2 lipids than JUb44 - why then does linoleic acid supplementation rescue N. parisii growth? Perhaps the profile of PUFAs in worms grown on BigB0170 is unusual is some way; however, to determine whether this is the case one would expect a comparison of PUFA profiles across worms fed a larger number of different bacterial diets, e.g., the entire CeMbio set. Another example is the spore incubation with bacterial supernatant - it's entirely unclear what is happening in this experiment. Possibilities include absorption of metabolites by the spores that then affect the worm or the infection process, in addition to direct effects of metabolites in the supernatants on the spores. To this reviewer, it is also unclear what "destruction" of spores could entail – their physical disintegration? Like many other experimental observations, neither the role of PUFAs for microsporidia infection nor the mechanisms by which bacterial supernatant affect spores are investigated further. Interpretation of the many different observations reported in the manuscript is further made difficult by the lack of explanatory detail. For example, the description of the activity-guided fractionation is insufficient, since it is unclear as to whether the fractions containing the massetolids are active, and if so, whether they represented the most strongly active fractions, what concentrations the compounds were found at in the active fractions and whether, etc.

Lastly, using superficial "fitness" measurements, as done here, to compare the effects of different bacteria on disease susceptibility is problematic. C. elegans fitness as measured in the lab may not be indicative of fitness in the wild, where the animal is exposed to a complete different environment including diverse biotic and abiotic challenges. The authors should be cautious here (and elsewhere) not to overinterpret their observations.

Taken together I find that this manuscript reports a vast and diverse assemblage of preliminary results that support the idea that bacteria may play an important role in susceptibility of C. elegans to microsporidia infection. However, the precise mechanisms of any of the observed effects are not elucidated. Instead of jumping from one bacterial system to the next, it would seem more fruitful to carefully dissect one of the systems - e.g. the effects of Bigb0170.

Minor points:
Figure 1: Parts of Figure 1 panels a through e appear to be cut off.
Figure 4: the condition "LB" is not explained in the legend (presumably this refers to incubation with LB media, used as control).
Line 376/377: Yet another example for rather preliminary results that are difficult to interpret but could represent a starting point for a potentially interesting study.
line 413: "…immune activation by the microbiome may play a minor role…" This is just speculation. No experiments are conducted to test this hypothesis.
line 698: Unclear what the is meant here. Even trace amounts of any metabolite are sufficient to obtain mass spectrometric data. Amounts of several microgram are needed only if NMR spectroscopic characterization was to be attempted.
line 808: "(b) Ion counts of 808 PUFA triglycerides containing fewer than 17 carbons in N2 animals grown on the different bacterial diets." It is difficult to imagine (perhaps impossible?) the structure of any PUFA triglycerides that have less than 17 carbons.

Reviewer #3

(Remarks to the Author)
In this study, El Jarkass et al. demonstrate that natural microbiota isolates of C. elegans are able to influence infection with a natural eukaryotic obligate intracellular parasite, the microsporidia N. parisii. The authors screen a collection of natural microbiota isolates and identify two Bacteroidetes bacteria, S. multivorum BIGb0170 and C. scopthalmum JUb44, which decrease N. parisii sporulation in the host. Using untargeted metabolomics, they determine that S. multivorum BIGb0170 and C. scopthalmum JUb44 affect host unsaturated lipid levels. El Jarkass et al. are able to pinpoint linoleic acid as the limiting factor for N. parisii growth on S. multivorum BIGb0170. The authors also identify two isolates, Pseudomonas lurida and Pseudomonas mendocina, which secrete molecules that inactivate N. parisii spores and confer protection against

infection. For P. lurida Myb11 they determine that it inhibits N. parisii through the production of massetolides. Furthermore, the authors establish that several Pseudomonas strains significantly reduce N. parisii infection and that a mixture of Pseudomonas species can greatly limit the amount of infection in C. elegans populations over many generations.

The work is highly significant and very timely. It hits on a very important subject and describes important ecological interactions of a host-microbiota-pathogen (natural infection route, important intracellular pathogen), specifically showing how susceptibility might be affected in a more natural state, given that all previous work has been done with microsporidia infection of worms grown on the standard lab bacterial food (the E. coli strain OP50). While the role of the natural C. elegans microbiota in pathogen defense has been demonstrated for viral and bacterial pathogens (Kissoyan et al. 2019 https://doi.org/10.1016/j.cub.2019.01.050; Montalvo-Katz et al. 2013 https://doi.org/10.1128/iai.00653-12; Vassallo et al. 2023 https://doi.org/10.7554/eLife.92534.3; González and Félix 2024 https://doi.org/10.1371/journal.ppat.1011947), it is unknown if the C. elegans microbiota influences infection with eukaryotic obligate intracellular parasites. The authors thoroughly investigate the influence of naturally associated bacteria on different N. parisii infection stages. The study highlights nutritional impacts of the microbiota on the host when challenged with microsporidia, as well as direct interactions between bacterial microbiota and microsporidia. The authors are able to identify two metabolites, massetolide E and massetolide F, that reduce the number of microsporidia spores. While the molecules identified are known antifungal and antibacterial compounds and while it is known that P. lurida MYb11 produce massetolide E (Kissoyan et al. 2019 https://doi.org/10.1016/j.cub.2019.01.050), less is known about their function in providing protection against microsporidia infection. In addition, this study provides a broader wealth of information for Pseudomonas isolates that are naturally associated with C. elegans and provide protection against microsporidia infection. Altogether, the study highlights the critical role of host-microsporidia-bacteria interactions in determining microsporidia infection in C. elegans and demonstrates that this model organism can be used to identify bacteria that produce anti-microsporidia compounds.

I have one major comment concerning the interpretation of the results. For this paper, El Jarkass et al. first focus on two C. elegans microbiota isolates, S. multivorum BIGb0170 and C. scopthalmum JUb44, which affect infection by N. parisii as first assessed by a reduction in the fraction of worms producing spores. However, worms on these two isolates are initially more infected. Also, S. multivorum BIGb0170 and C. scopthalmum JUb44 were previously reported to result in developmental delay of C. elegans (Dirksen et al., 2020 https://doi.org/10.1534/g3.120.401309; Samuel et al., 2016 https://doi.org/10.1073/pnas.1607183113) and worms exposed to N. parisii and grown on S. multivorum BIGb0170 and C. scopthalmum JUb44 displayed reduced fitness (this study). These data indicate that the two bacterial isolates represent ‘poor quality’ food for the worms and do not provide all the required nutrients for the worms to develop and produce progeny, as also discussed in this study. Indeed, metabolomic analysis revealed that these diets caused a disruption to host unsaturated lipid levels (this study). The authors establish that linoleic acid is the limiting host factor for N. parisii growth on S. multivorum BIGb0170 (the limiting host factor(s) on C. scopthalmum JUb44 remain unknown) and conclude that the bacterial C. elegans microbiome influences microsporidia infection through nutrient limitation. Because the authors emphasize that worms exposed to these two bacteria exhibit reduced pathogen load the reader gets the impression that the microbiota isolates are protective or beneficial for the host. However, since the bacteria represent ‘poor quality’ food and worms on the two microbiota isolates are more infected and show reduced fitness the observed impact on pathogen load can only be regarded as an indirect, positive side effect of poorly nourished worms (a poorly nourished host cannot be exploited by the parasite as well as a well-nourished host). It may be more prudent to use wording that more carefully discusses the different, positive and negative, impacts of S. multivorum BIGb0170 and C. scopthalmum JUb44 on the nematode host.

Minor comments:

The methodology is sound. However, in the methods section I miss the following information:
1. How exactly was host fitness determined (% gravid)?
2. Statistical analysis of data: Figures 1-6, In the figure legend it is stated that “Data is from three independent replicates of […] worms each.” Does this mean that the data of three experimental runs was pooled? Why were the data pooled? Did the authors want to increase statistical power or to decrease noise from particular experiments? When the data from several experimental runs is not kept separate, variation across experimental (biological) replicates cannot be evaluated.

There is a problem with the designation of the bacterial strains:
Line178 and before: S. multivorum BIGb0170
Line 184 and onwards: C. scopthalmum BIGb0170
This is obviously confusing and I hope that I understood correctly that both isolates increased invasion by N. parisii , linoleic acid only affects N. parisii growth on S. multivorum BIGb0170 (not on C. scopthalmum JUb44), oleic acid has an effect on pumping and sporoplasm invasion only on S. multivorum BIGb0170 (not on C. scopthalmum JUb44).

Line 242 onwards: “To determine if vitamin B12 is produced in P. lurida MYb11, we monitored the expression levels of acyl-CoA dehydrogenase acdh-1, a dietary sensory of B12 deficiency (Watson et al. 2014). When Pacdh-1::gfp L1 animals were grown on lawns of Comamonas aquatica DA1877, a bacterial species known to produce vitamin B12, or P. lurida MYb11, GFP expression was repressed (Figure S6a).” Repression of Pacdh-1::gfp
expression by P. lurida MYb11 was recently demonstrated by Pees et al. 2024 https://doi.org/10.1128/mbio.03463-23.

Version 1:

Reviewer comments:

Reviewer #1

(Remarks to the Author)
I thank the authors for their thoughtful responses. However, I still feel that points 10 and 12 have not been adequately addressed. Regarding comment 10, using a one-way ANOVA for all experiments with more than two samples is not acceptable, especially for a journal like Nature Communications. While examples of poor statistical analysis can be found in the literature, the current standard requires authors to perform normality tests (for example, these are available as a simple function in GraphPad Prism, which includes four types of tests). Based on the data shown in the graphs, the authors' claim that the sample size is insufficient for normality tests seems highly unlikely. Even if this were the case, the tests would report that the sample size is too small. If the data are not normally distributed, non-parametric tests (e.g., Kruskal-Wallis test) should be used, which are also readily available in programs like GraphPad.

For point 12, the authors justify additional incubation of bacterial lawns at room temperature to ensure enough food for the worms. However, this undermines the purpose of standardizing culture density by reaching a similar OD600, as bacterial cultures grow at different rates at room temperature. The authors should explicitly acknowledge that bacterial culture density was not adequately controlled. The reasoning of needing sufficient food is also not acceptable, as the authors could have grown larger volumes of bacteria, concentrated them by centrifugation, adjusted to the desired OD600, and spotted plates that were ready to be used.

Reviewer #2

(Remarks to the Author)
In my review of the initial submission I pointed out that changing bacterial diets can affect host physiology in very many different ways, directly and indirectly. Therefore, mechanistic insights are critical for evaluating any effects of bacteria on host physiology, and C. elegans provides unique opportunities to do this.

However, the revised manuscript still falls short of providing any substantial mechanistic insights. The observations this study is based on - (i) that two bacteria on which C. elegans grows poorly are associated with increased initial microsporidia invasion but reduced pathogen loads, and (ii) that Pseudomonas strains reduce infection, likely by producing massetolide or another natural product - are interesting, but not studied to any depth. Let me give a few examples for follow-up experiments that would be expected.

To follow up on the observation that worms on C. scopthalmum and S. multivorum have increased initial microsporidia invasion but reduced pathogen loads, one would compare the worm's metabolome and proteome or transcriptome on these two bacteria with thos of OP50-fed worms. This was not done, instead the authors focused on lipidomics, for unclear reasons. Then, following up on the finding that worms on C. scopthalmum and S. multivorum have altered levels of unsaturated fatty acids, one would measure expression of a series of fatty acid desaturases, not only fat-2 (see here for the full pathway https://pmc.ncbi.nlm.nih.gov/articles/PMC2665873/). Next one would test – as the authors did – whether fat-2 mutants (or mutants of fat-7 or another component of lipid metabolism, selected based on the comparative transcriptomics or proteomics data) display reduced pathogen burden, and whether this can be rescued by adding the putative product of FAT-2, linoleic acid. The results of this experiment (shown in Figure 2d) demonstrate that adding linoleic acid, even at a high level, does not rescue the phenotype. Linoleic acid has almost no effect on the very low pathogen burden of fat-2 mutant worms fed OP50. BiGb0170-fed worms do show some increase of pathogen burden, but it's unclear how that can be interpreted, given the OP50 result, and given that supplementing linoleic acid to BiGb0170 may simply rescue some of the developmental delay the worms experience on this bacteria. In fact, BigB0170-fed fat-2 mutant worms supplemented with linoleic acid have a higher pathogen load than OP-50-fed fat-2 worms supplemented with linoleic acid, strongly indicating that linoleic acid production is not the key determinant here.

Similarly, the theory that production of massetolide or another natural product is responsible for the protective effects of Pseudomonas, at a minimum, needs to be followed up by testing a bacterial mutant that are deficient in the production of massetolides (see e.g., here https://pmc.ncbi.nlm.nih.gov/articles/PMC2293227/).

These are just simple examples for where very basic follow up experiments are missing or the interpretation of the data is not consistent or backed up by additional lines of evidence.

Reviewer #3

(Remarks to the Author)
The authors have addressed all of my concerns and also most of the concerns of the other reviewers, which further improved the manuscript. I have no additional comments.

Version 2:

Reviewer comments:

Reviewer #1

(Remarks to the Author)
The authors have appropriately addressed my concerns.

Reviewer #2

(Remarks to the Author)
The authors have added new data that show that massetolid biosynthesis is required for the inhibitory activity of P. lurida MYb11. This addresses one major concern of my previous review. However, most of the hypotheses put forward to explain the effects of bacteria on N. parisii infection of C. elegans are either not well supported or not further pursued.

1. The role of linoleic acid. What remains unanswered in whether worms fed BIGb0170 indeed have lower 18:2 levels, and also in what other ways the FA profile may differ. The authors need to profile relative abundances of different FAs in animals grown on the different bacteria, most importantly, a comparison of 18:2 levels is needed. This is best done via total hydrolysis of all lipids (see work by Watts et al.). Unless data for changes in 18:2 abundance in the animals are presented, the author's model for the role of changes in 18:2 production remains entirely speculative.

2. Line 271 - Here the hypothesis that bacterial supernatant causes in vitro spore destruction is put forward. However, this is not further explained and I have to admit that to me it's unclear how spores could simply disappear as a result of exposure to bacterial culture supernatant.

3. The purpose of the paragraph starting at line 295 is entirely unclear. It would be interesting to identify other anti-microsporidia compounds produced by Pseudomonas, but this is not done - the main text mentions "activity-guided purification" but this seems to end after a single Combiflash fractionation, which yielded ranged of fractions with activity.

4. Similarly, whether in fact viscosin production underlies is not tested. This lack of follow-up essentially renders the preceding comparative antismash analysis of the genomes of different inhibitory and non-inhibitory species useless.

5. The purpose of the multi-generation experiment is unclear - the fact that protection does not wane after one generation is supportive of the idea that specific compounds produced by the bacteria inhibit or prevent infection, but seems otherwise unremarkable.

I'm left with the impression of an assemblage of interesting observations that all show that bacteria have significant, multifaceted influence on the susceptibility of C. elegans to N. parissii infection. Most of these observations are followed up upon only superficially, and thus mechanistic hypotheses either cannot be developed or remain exceedingly speculative. The exception is the case of the massetolids, but these compounds have previously been shown to be protective of pathogen infection in C. elegans.

Open Access This Peer Review File is licensed under a Creative Commons Attribution 4.0 International License, which permits use, sharing, adaptation, distribution and reproduction in any medium or format, as long as you give appropriate

credit to the original author(s) and the source, provide a link to the Creative Commons license, and indicate if changes were made.
In cases where reviewers are anonymous, credit should be given to 'Anonymous Referee' and the source.
The images or other third party material in this Peer Review File are included in the article's Creative Commons license, unless indicated otherwise in a credit line to the material. If material is not included in the article's Creative Commons license and your intended use is not permitted by statutory regulation or exceeds the permitted use, you will need to obtain permission directly from the copyright holder.
To view a copy of this license, visit https://creativecommons.org/licenses/by/4.0/

We thank the reviewers for their comments. We have provided a revised manuscript (where all changes are highlighted) to address the reviewers concerns. Points raised by the reviewers have been incorporated into the manuscript (including several additional figure panels) and we have provided a point-by-point response to each of the reviewer's comments.

REVIEWER COMMENTS

Reviewer #1 (Remarks to the Author):

Review for "The Caenorhabditis elegans bacterial microbiome influences microsporidia infection through nutrient limitation and inhibiting parasite invasion," El Jarkass et al.
Remarks to the authors

In the manuscript titled "The Caenorhabditis elegans bacterial microbiome influences microsporidia infection through nutrient limitation and inhibiting parasite invasion," El Jarkass et al. analyzed the antimicrosporidial properties of various bacteria in the natural microbiome of C. elegans. This thorough study identifies several bacterial species that are detrimental to microsporidia, either directly or indirectly, and elucidates mechanisms by which bacteria can enhance host survival during infection. The study provides detailed characterization of different infection phases, employs diverse approaches, and uncovers new mechanisms that limit microsporidia infection or damage spores.

We thank the reviewer for their comments.

Specific comments and questions:
1. Several figures (e.g., 1b-e and 3b-d) are not fully visible, and the data and/or statistical results are missing. This issue also exists in the bioRxiv preprint.

We apologize for this error that was introduced converting the word document to a PDF. This issue has now been corrected.

2. The rationale and setup for the experiment tracking N. parisii inhibition by Pseudomonas over multiple generations are unclear. What are the potential outcomes? How do authors ensure they transfer infected animals? Do they use an IPR reporter? Does the age of plates affect the results due to bacterial die-off?

In natural environments, populations of *N. parisii*-infected *C. elegans* may come in contact with protective bacterial species and this could reduce infection levels of these *C. elegans* populations over multiple generations. The experiment in figure 6e and f allows us to test the influence of the *Pseudomonas* isolates we identified under conditions that more closely match those found in nature. In other experiments in the manuscript, we show protective effects of the *Pseudomonas* isolates in controlled experiments where the spore dose is defined. One potential outcome of this multi-generation experiment is that spores produced by infected worms would overwhelm the protective ability of the bacteria and that over multiple generations, there is no protective effect. We see the opposite of this outcome, where the bacteria still display protective effects under these conditions, with the strongest protection coming from a mixture of Pseudomonas isolates. We have added additional reasoning for this experiment and the outcome we observe to the results on lines 361-363 and lines 376-378.

In this experiment, we randomly selected gravid animals to transfer. We are not attempting to transfer only infected animals, rather we are sampling a representative subset of the plate at each generation. Because of this we do not ascertain whether the animals are infected by using an IPR reporter. We have modified the results, figure legend 6, and the methods for this experiment to clarify this point.

Seeded plates were used for 4 days prior to transfer to a new seeded plate. Any unseeded plates were stored at 4C until use. After generation 7, a new batch of plates was seeded to prevent using plates that had been stored for too long. As the trends did not drastically change at generation 7 upon the use of this newly seeded batch plates, we do not anticipate bacterial die-off having a large effect on this experiment.

3. The narrative needs improvement. The rationale for focusing on specific bacterial strains is unclear. For example, the authors initially observed spore reduction with seven bacterial strains but followed up on two.

As the authors mention, we initially observed a statistically significant reduction in spores in seven bacterial strains, as shown in figure 1b. We then followed up on these seven strains in figure 1c by performing invasion assays. After observing high levels of sporoplasms in worms grown on JUb44 and BIGb0170, we chose to follow up on these two strains. We have modified the text in lines 105-106 and 113-115 to reflect that invasion represents the most critical step in infection and to help clarify why only JUb44 and BIGb0170 were the subject of follow up experiments.

4. The increased sporoplasm number in animals fed with BIGb0170 and JUb44 is attributed to increased pharyngeal pumping. Do other bacterial species that do not cause increased colonization have normal or decreased pumping rates?

We have now performed an additional experiment, where we test the 7 isolates from figure 1c that we tested for invasion. We only observe increased pharyngeal pumping rates for worms grown on BIGb0170 and JUb44. These data are shown figure S1e.

5. Does the growth rate of C. elegans on different bacterial food sources affect observed microsporidia infection levels?

In our set up, and as reported by Dirksen et al. 2020, the two bacteria that negatively impact *C. elegans* developmental rate are JUb44 and BIGb0170 which were the two bacteria that caused increased *N. parisii* invasion and decreased *N. parisii* spore production. As we demonstrate in our work, animals grown on these two bacterial strains display reduced pathogen burdens due to poor nutritional quality and poor nematode fitness overall.

Alternatively, growth on MYb11 results in developmental acceleration, a result of vitamin B12. Animals grown on MYb11 resulted in lower pathogen burdens overall even in the *metr-1 (ok521)* background indicating vitamin B12 independent mechanisms of protection (Figure S6c).

6. Does mixing spores with OP50 instead of specific microbiome species during infection alter the results?

For adult pulse infections animals were fed spores mixed with 10 μl of *E. coli* OP50 for one hour. Since *E. coli* OP50 cannot colonize the gut at this stage, we do not expect it to alter the results. Additionally, to avoid any potential impacts of small molecules on the spores prior to ingestion by nematodes, *E. coli* OP50 was used. In this way we were able to assess if altered invasion was a result of bacteria already present in the intestinal lumen, or a result of altered host physiology.

7. While lipidomics is part of metabolomics, "metabolomics" often describes hydrophilic molecule analysis. Consider using "lipidomics" to avoid confusion.

We have now replaced “metabolomics” with “lipidomics” throughout the manuscript.

8. In Fig. 2d, blue dots for fat-2 on NGM (and for BIGb0170) are not visible.

The data points in this condition are all at zero, and as the NGM condition is designated with black dots, they are not discernable from the X axis. To make these points visible, we have now represented the NGM condition in orange in Figure 2d and Supplementary Figures 3a and 4a.

9. Enhanced fitness and resistance to infection do not always correlate (e.g., vitamin B12 treatment). Yet, spores are described as less infective based on increased percentage of gravid animals. This is contradictory; more cautious wording is needed.

The following sentence: *“N. parisii spores incubated in the supernatants of P. lurida MYb11, P. mendocina MSPm1, and C. piscis BIGb0172 were less infective, as indicated by increased proportions of gravid adult animals* ***(Figure 4b)****.”* has now been reworded to the following:

“Nematodes infected with N. parisii spores incubated in the supernatants of P. lurida MYb11, P. mendocina MSPm1, and C. piscis BIGb0172 had increased proportions of gravid adult animals ***(Figure 4b)****.”*

10. One-way ANOVA was used for all assays. Do all datasets have a normal distribution?

Our data, as is similar with many biological studies, relies on performing statistical comparisons between 3 biological replicates. As such, it is difficult to determine if the data is normally distributed given the small sample size. Using one-way ANOVA is a common approach to determine statistical significance with these sample sizes and has been used in *C. elegans*-microsporidia papers from other labs (for example, Balla et al, Nature Microbiology 2016 and our lab (for example Willis and Zhao et al., Science Advances 2021). We have now changed all of our bar graphs so that the data points are shown.

11. The legend for Fig. 6c states three replicates, but the graph suggests one (no bars; if SD values are too small, explain).
This experiment is composed of three biological replicates, but the error bars were inadvertently omitted from the figure. Error bars have now been added in the revised figure.

12. The authors adjust the optical density of bacteria prior to seeding the plates. However, they additionally incubate the plates and then store them at lower temperatures. These treatments

likely affect different bacterial species differently (e.g., some might grow faster after seeding, while others might die off more quickly at the storage temperatures). Therefore, it is difficult to imagine that bacterial density remains consistent across all tested species at the time of the experiments. Could this variability affect the results shown in this study?

Plates were seeded and incubated for 24 hours to promote bacterial growth to sustain a population for 72 hours. This was done to avoid starving out the populations thus impacting *N. parisii* replication. Every replicate had freshly seeded plates prior to use with a maximal storage time of 3 days at 4C when infections were initiated in 72-hour old adults. Storage at 4C was for 3 days prior to use to ensure that nematodes were transferred onto bacteria originating from the same colony, maintaining consistency.

While we cannot know for certain how the bacteria are impacted at different temperatures, the consistent replicability of our results, and the lack of starved nematodes on assay plates suggest that this does not impact the study results.

13. The authors refer to the microbiome even when some bacterial species seem to be nutritionally insufficient and therefore indirectly suppress pathogen development. It would be useful to perform additional experiments to determine if the role of some bacteria is purely nutritional and not required for colonizing the intestine of C. elegans.

In our manuscript, we built upon the work of several groups who have isolated and characterized bacteria associated with *C. elegans*. The bacteria we used are part of the CeMbio collection which were selected based on being commonly associated with *C. elegans* and representing the diversity of bacteria associated with *C. elegans*. Our goal was to use this collection to understand how bacteria can impact microsporidia infection.

In the process of characterizing bacteria-microsporidia interactions we found several effects of bacteria on *C. elegans* physiology that was independent of microsporidia infection, such as pharyngeal pumping rate and lipid composition. We feel that further characterization of these bacterial isolates on the growth and physiology of *C. elegans* is beyond the scope of our study.

14. In lines 410-411, the claim that “7% of bacteria associated with C. elegans activate this immune response” seems incorrect. The referenced study (Gonzalez and Felix) found that 5/71 bacterial species induced IPR reporter expression stronger than OP50 in the presence of the Orsay virus, not necessarily in the absence of the pathogen.

We incorrectly understood the experiment reported in this paper and we thank the reviewer for catching our error. In a separate experiment in this paper, the authors reported that none of the 71 bacterial strains they tested induce an IPR reporter strain. We have now changed this line in the discussion to reflect the correct experimental result from this study.

15. Why were 11 instead of 12 CeMbio strains tested in the initial screen?

The missing strain JUb134 grows at a substantially different rate in liquid culture compared to the rest of the strains. Therefore, it was omitted from the screening. This is also indicated in our methods section under “Bacterial growth and Maintenance: <u>JUb134 was excluded as it did not grow at a similar rate to other strains in overnight cultures</u>.”

16. Inconsistent naming: BIGb0170 is referred to as S. multivorum and S. scopthalmum.

Corrections have been made to only refer to BIGb0170 as *S. multivorum*.

17. Consider referencing additional studies on microsporidia infections in C. elegans (e.g., PMIDs 24945527, 35544001, 38702921) and add a reference to line 436.

We have now cited PMIDs 24945527, 35544001, 38702921 and have now added the reference Kissoyan et al. 2019 to line 436.

18. Consider rephrasing “more gravid” as it is a binary condition.

We have now modified these instances to make clear that we are referring to increased gravidity of the population and not the individual.

Reviewer #2 (Remarks to the Author):

The manuscript by El Jarkass et al. reports that feeding C. elegans different bacteria affects their susceptibility to infection with microsporidia. Building evidence indicates that host-associated bacteria play a central role for the susceptibility of animals, including humans, to infection by diverse pathogens, and C. elegans is an excellent model system to investigate the underlying mechanisms, as the authors point out in the introduction. However, the manuscript falls short of taking advantage of the opportunities this model system offers for mechanistic study.

We thank the reviewer for their comments and as we discuss below, we disagree with the reviewer’s view that our work does not provide mechanistic insight.

Comments on the Results section:

Many of the results seem ambiguous and confusing, which is problematic for their interpretation given that changing bacterial diets can affect host physiology in very many different ways, directly and indirectly. For example, Bigb0170 seems to have more 18:2 lipids than JUb44 - why then does linoleic acid supplementation rescue N. parisii growth? Perhaps the profile of PUFAs in worms grown on BigB0170 is unusual is some way; however, to determine whether this is the case one would expect a comparison of PUFA profiles across worms fed a larger number of different bacterial diets, e.g., the entire CeMbio set.

The goal of the lipidomic experiments was to understand what metabolites are different on worms grown on JUb44 or Bigb0170 that reduce levels of microsporidia infection. We were able to show that linoleic acid supplementation was able to rescue *N. parisii* growth on Bigb0170. Although we do not know why *N. parisii* infection of worms grown on JUb44 is not rescued by linoleic supplementation, we were able to show that the triglyceride profile of worms on these two bacteria are different and that mixing the bacteria restores *N. parisii* growth. Although we think that lipidomic profiling of worms grown on all the strains of the CeMbio set would be interesting, we feel that this is experiment is beyond the scope of our study and instead focused on the only two bacterial strains that caused increased microsporidia invasion and decreased spore production.

Another example is the spore incubation with bacterial supernatant - it's entirely unclear what is happening in this experiment. Possibilities include absorption of metabolites by the spores that then affect the worm or the infection process, in addition to direct effects of metabolites in the supernatants on the spores. To this reviewer, it is also unclear what "destruction" of spores could entail – their physical disintegration?

Although we have not directly observed spores being physically disintegrated, we observe a decrease in the number of spores, suggesting that they are being destroyed. In supplementary figure 7 we show that spores incubated in bacterial supernatant have the same level of infectivity whether we directly infect worms or wash the spores before infection. If there were metabolites that were affecting the infection we would anticipate noticing a difference in infection levels in this experiment. The molecule we identify as have anti-microsporidia activity, massetolide, has been reported to lyse other types of cells. We believe that taken together, our data supports a model of these *Pseudomonas* bacteria producing molecules which act directly on spores to prevent *N. parisii* invasion of *C. elegans*.

Like many other experimental observations, neither the role of PUFAs for microsporidia infection nor the mechanisms by which bacterial supernatant affect spores are investigated further.

We feel that identifying a metabolic pathway that influences microsporidia infection is a large advancement in our knowledge of how bacterial diet can influence host metabolism to effect microsporidia infection. Further, we show for the first time that *Pseudomonas* species secrete molecules which can inhibit microsporidia spores, identifying one of these molecules and providing biochemical and bioinformatic support for a diverse set of molecules being produced. Although we agree with the reviewer that further investigation into these topics would be interesting, we feel that this is beyond the scope of our study.

Interpretation of the many different observations reported in the manuscript is further made difficult by the lack of explanatory detail. For example, the description of the activity-guided fractionation is insufficient, since it is unclear as to whether the fractions containing the massetolids are active, and if so, whether they represented the most strongly active fractions, what concentrations the compounds were found at in the active fractions and whether, etc.

We have added in data from activity guided purification of *P. lurida* MYb11 in figure 5a to demonstrate that the active fractions 9-11 were pooled and used for the identification of Massetolides.

Lastly, using superficial "fitness" measurements, as done here, to compare the effects of different bacteria on disease susceptibility is problematic. C. elegans fitness as measured in the lab may not be indicative of fitness in the wild, where the animal is exposed to a complete different environment including diverse biotic and abiotic challenges. The authors should be cautious here (and elsewhere) not to overinterpret their observations.

We agree that fitness measurements in the lab are not necessarily indicative of fitness in the wild. Indeed, the motivation of ours and others in the field of using natural pathogens and naturally-associated bacteria is to more closely align laboratory experiments with what is observed in nature. However, we feel that our measurements of fitness are a useful approach to compare relative fitness between different conditions in a controlled laboratory environment. We have now directly addressed this limitation in the discussion in lines 389-391.

Taken together I find that this manuscript reports a vast and diverse assemblage of preliminary

results that support the idea that bacteria may play an important role in susceptibility of C. elegans to microsporidia infection. However, the precise mechanisms of any of the observed effects are not elucidated. Instead of jumping from one bacterial system to the next, it would seem more fruitful to carefully dissect one of the systems - e.g. the effects of Bigb0170.

We disagree with the reviewer's assessment that our manuscript is a collection of preliminary results. We feel that our study has contributed two main advances to the understanding of how bacteria influence infectious diseases. One, we show that a Bacteroidetes species alters unsaturated fatty acid metabolism in *C. elegans* and that linoleic acid is a limiting nutrient for *N. parisii* growth. Two, we show that *Pseudomonas* species make a variety of molecules which inactivate *N. parisii* spores. Although we agree with the reviewer that more detailed studies on the effects of these bacteria on lipid metabolism and the mechanism of inhibition of these molecules are interesting topics for further investigation, we feel that each of these lines of inquiry could be its own manuscript and thus is beyond the scope of this current study.

Minor points:
Figure 1: Parts of Figure 1 panels a through e appear to be cut off.

We apologize for this error that was introduced converting the word document to a PDF. This issue has now been corrected.

Figure 4: the condition "LB" is not explained in the legend (presumably this refers to incubation with LB media, used as control).

We have now specified that LB is lysogeny broth and modified figure legend 4 to indicate that LB media is used as a control.

Line 376/377: Yet another example for rather preliminary results that are difficult to interpret but could represent a starting point for a potentially interesting study.

As we mention above in our response to reviewer 1, we have now modified how we describe the motivation for this experiment (figure 6e and f) and how we interpret the results. These changes are included in the last paragraph of the results section.

line 413: "…immune activation by the microbiome may play a minor role…" This is just speculation. No experiments are conducted to test this hypothesis.

We have now modified this part of the discussion to make it clear that we are speculating based upon others results and not proposing a hypothesis that we tested.

line 698: Unclear what the is meant here. Even trace amounts of any metabolite are sufficient to obtain mass spectrometric data. Amounts of several microgram are needed only if NMR spectroscopic characterization was to be attempted.

We have now modified this line to clarify that we were unable to recover any material for further experimentation.

line 808: "(b) Ion counts of 808 PUFA triglycerides containing fewer than 17 carbons in N2 animals grown on the different bacterial diets." It is difficult to imagine (perhaps impossible?) the structure of any PUFA triglycerides that have less than 17 carbons.

In figure 2b we are quantifying ion counts of triglycerides containing both a PUFA acyl chain and an acyl chain containing fewer than 17 carbons. We have now modified the figure legend to make it clear what we are quantifying and that the chain with less than 17 carbons is not necessarily a PUFA.

Reviewer #3 (Remarks to the Author):

In this study, El Jarkass et al. demonstrate that natural microbiota isolates of C. elegans are able to influence infection with a natural eukaryotic obligate intracellular parasite, the microsporidia N. parisii. The authors screen a collection of natural microbiota isolates and identify two Bacteroidetes bacteria, S. multivorum BIGb0170 and C. scopthalmum JUb44, which decrease N. parisii sporulation in the host. Using untargeted metabolomics, they determine that S. multivorum BIGb0170 and C. scopthalmum JUb44 affect host unsaturated lipid levels. El Jarkass et al. are able to pinpoint linoleic acid as the limiting factor for N. parisii growth on S. multivorum BIGb0170. The authors also identify two isolates, Pseudomonas lurida and Pseudomonas mendocina, which secrete molecules that inactivate N. parisii spores and confer protection against infection. For P. lurida Myb11 they determine that it inhibits N. parisii through the production of massetolides. Furthermore, the authors establish that several Pseudomonas strains significantly reduce N. parisii infection and that a mixture of Pseudomonas species can greatly limit the amount of infection in C. elegans populations over many generations.

The work is highly significant and very timely. It hits on a very important subject and describes important ecological interactions of a host-microbiota-pathogen (natural infection route, important intracellular pathogen), specifically showing how susceptibility might be affected in a more natural state, given that all previous work has been done with microsporidia infection of worms grown on the standard lab bacterial food (the E. coli strain OP50). While the role of the natural C. elegans microbiota in pathogen defense has been demonstrated for viral and bacterial pathogens (Kissoyan et al. 2019 https://doi.org/10.1016/j.cub.2019.01.050; Montalvo-Katz et al. 2013 https://doi.org/10.1128/iai.00653-12; Vassallo et al. 2023 https://doi.org/10.7554/eLife.92534.3; González and Félix 2024 https://doi.org/10.1371/journal.ppat.1011947), it is unknown if the C. elegans microbiota influences infection with eukaryotic obligate intracellular parasites. The authors thoroughly investigate the influence of naturally associated bacteria on different N. parisii infection stages. The study highlights nutritional impacts of the microbiota on the host when challenged with microsporidia, as well as direct interactions between bacterial microbiota and microsporidia. The authors are able to identify two metabolites, massetolide E and massetolide F, that reduce the number of microsporidia spores. While the molecules identified are known antifungal and antibacterial compounds and while it is known that P. lurida MYb11 produce massetolide E (Kissoyan et al. 2019 https://doi.org/10.1016/j.cub.2019.01.050), less is known about their function in providing protection against microsporidia infection. In addition, this study provides a broader wealth of information for Pseudomonas isolates that are naturally associated with C. elegans and provide protection against microsporidia infection. Altogether, the study highlights the critical role of host-microsporidia-bacteria interactions in determining microsporidia infection in C. elegans and demonstrates that this model organism can be used to identify bacteria that produce anti-microsporidia compounds.

We thank the reviewer for their comments.

I have one major comment concerning the interpretation of the results. For this paper, El

Jarkass et al. first focus on two C. elegans microbiota isolates, S. multivorum BIGb0170 and C. scopthalmum JUb44, which affect infection by N. parisii as first assessed by a reduction in the fraction of worms producing spores. However, worms on these two isolates are initially more infected. Also, S. multivorum BIGb0170 and C. scopthalmum JUb44 were previously reported to result in developmental delay of C. elegans (Dirksen et al., 2020 https://doi.org/10.1534/g3.120.401309; Samuel et al., 2016 https://doi.org/10.1073/pnas.1607183113) and worms exposed to N. parisii and grown on S. multivorum BIGb0170 and C. scopthalmum JUb44 displayed reduced fitness (this study). These data indicate that the two bacterial isolates represent 'poor quality' food for the worms and do not provide all the required nutrients for the worms to develop and produce progeny, as also discussed in this study. Indeed, metabolomic analysis revealed that these diets caused a disruption to host unsaturated lipid levels (this study). The authors establish that linoleic acid is the limiting host factor for N. parisii growth on S. multivorum BIGb0170 (the limiting host factor(s) on C. scopthalmum JUb44 remain unknown) and conclude that the bacterial C. elegans microbiome influences microsporidia infection through nutrient limitation. Because the authors emphasize that worms exposed to these two bacteria exhibit reduced pathogen load the reader gets the impression that the microbiota isolates are protective or beneficial for the host. However, since the bacteria represent 'poor quality' food and worms on the two microbiota isolates are more infected and show reduced fitness the observed impact on pathogen load can only be regarded as an indirect, positive side effect of poorly nourished worms (a poorly nourished host cannot be exploited by the parasite as well as a well-nourished host).

It may be more prudent to use wording that more carefully discusses the different, positive and negative, impacts of S. multivorum BIGb0170 and C. scopthalmum JUb44 on the nematode host.

The following sentence has been added to the discussion to help clarify this point.

"Thus, while infected nematodes grown on *S. multivorum* BIGb0170 or *C. scopthalmum* JUb44 display overall reduced pathogen loads, this is likely due to the poor growth conditions of the host. Given the extreme host dependence displayed by microsporidia, poor host resources may not support robust *N. parisii* growth, thereby indirectly protecting the host from infection."

Minor comments:

The methodology is sound. However, in the methods section I miss the following information:
1. How exactly was host fitness determined (% gravid)?

The following statement was added to the methods under" Microscopy and Image quantification" to help clarify this point:
"Gravidity is defined as the presence of at least one embryo per worm and the fraction of gravid adults is defined as the number of animals containing at least one embryo over the total number of animals quantified"

2. Statistical analysis of data: Figures 1-6, In the figure legend it is stated that "Data is from three independent replicates of […] worms each." Does this mean that the data of three experimental runs was pooled? Why were the data pooled? Did the authors want to increase statistical power or to decrease noise from particular experiments? When the data from several experimental runs is not kept separate, variation across experimental (biological) replicates

cannot be evaluated.

The data was displayed in this way to show the individual data points across the replicates. The mean and the SD displayed in figures represents the average of three independent replicates, not the average of all data points pooled together as one. We have now modified the bar graphs to show the biological replicates as individual points. Additionally, the following statement has been added to the methods section under “Statistical Analysis” for added clarity:
“The mean and standard deviations were calculated based on three individual biological replicates.”

There is a problem with the designation of the bacterial strains:
Line178 and before: S. multivorum BIGb0170
Line 184 and onwards: C. scopthalmum BIGb0170
This is obviously confusing and I hope that I understood correctly that both isolates increased invasion by N. parisii , linoleic acid only affects N. parisii growth on S. multivorum BIGb0170 (not on C. scopthalmum JUb44), oleic acid has an effect on pumping and sporoplasm invasion only on S. multivorum BIGb0170 (not on C. scopthalmum JUb44).

We inadvertently mislabelled the species name of this strain and corrections have now been made throughout the manuscript to clearly indicate BIGb0170 as *S. multivorum.*

Line 242 onwards: “To determine if vitamin B12 is produced in P. lurida MYb11, we monitored the expression levels of acyl-CoA dehydrogenase acdh-1, a dietary sensory of B12 deficiency (Watson et al. 2014). When Pacdh-1::gfp L1 animals were grown on lawns of Comamonas aquatica DA1877, a bacterial species known to produce vitamin B12, or P. lurida MYb11, GFP expression was repressed (Figure S6a).” Repression of Pacdh-1::gfp
expression by P. lurida MYb11 was recently demonstrated by Pees et al.
2024 https://doi.org/10.1128/mbio.03463-23.

We have now included this reference on line 237.

We thank the reviewers for their comments. We have provided a revised manuscript (where all changes are highlighted) to address the reviewers concerns. Points raised by the reviewers have been incorporated into the manuscript (including several additional figure panels) and we have provided a point-by-point response to each of the reviewer's comments. We apologize for the long delay between revisions, which was due to the first author leaving the lab and thus needing to have additional personnel complete the requested experiments and revisions.

REVIEWER COMMENTS

Reviewer #1 (Remarks to the Author):

I thank the authors for their thoughtful responses.

We thank the reviewer for their comments.

However, I still feel that points 10 and 12 have not been adequately addressed. Regarding comment 10, using a one-way ANOVA for all experiments with more than two samples is not acceptable, especially for a journal like Nature Communications. While examples of poor statistical analysis can be found in the literature, the current standard requires authors to perform normality tests (for example, these are available as a simple function in GraphPad Prism, which includes four types of tests). Based on the data shown in the graphs, the authors' claim that the sample size is insufficient for normality tests seems highly unlikely. Even if this were the case, the tests would report that the sample size is too small. If the data are not normally distributed, non-parametric tests (e.g., Kruskal-Wallis test) should be used, which are also readily available in programs like GraphPad.

We apologize for our earlier response where we mistakenly said that normality tests could not be performed with only three biological replicates. We have now tested data normality using several statical tests on the residuals of ANOVA tests. All of our experiments consisted of normally distributed data, as indicated by not having statistically significant values in at least one of these tests. We have included the results of these tests in a newly added Supplementary Table 6.

Additionally, as we were performing these normality tests, we realized that some of our previous statistical tests were performed on in individual data points within a biological replicate, instead of on the means of the replicates. We have now fixed these errors as well as removing some panels of data that were not significant. Removing these data did not change any of our main conclusions.

For point 12, the authors justify additional incubation of bacterial lawns at room temperature to ensure enough food for the worms. However, this undermines the purpose of standardizing culture density by reaching a similar OD600, as bacterial cultures grow at different rates at room temperature. The authors should explicitly acknowledge that bacterial culture density was not adequately controlled. The reasoning of needing sufficient food is also not acceptable, as the authors could have grown larger volumes of bacteria, concentrated them by centrifugation, adjusted to the desired OD600, and spotted plates that were ready to be used.

We agree that once the bacteria was added to plates that the density of the cultures was no longer controlled. We have modified the methods in line 472 to explicitly acknowledge this.

Reviewer #2 (Remarks to the Author):

In my review of the initial submission I pointed out that changing bacterial diets can affect host physiology in very many different ways, directly and indirectly. Therefore, mechanistic insights are critical for evaluating any effects of bacteria on host physiology, and C. elegans provides unique opportunities to do this.

We thank the reviewer for their comments.

However, the revised manuscript still falls short of providing any substantial mechanistic insights. The observations this study is based on - (i) that two bacteria on which C. elegans grows poorly are associated with increased initial microsporidia invasion but reduced pathogen loads, and (ii) that Pseudomonas strains reduce infection, likely by producing massetolide or another natural product - are interesting, but not studied to any depth. Let me give a few examples for follow-up experiments that would be expected.

To follow up on the observation that worms on C. scopthalmum and S. multivorum have increased initial microsporidia invasion but reduced pathogen loads, one would compare the worm's metabolome and proteome or transcriptome on these two bacteria with thos of OP50-fed worms. This was not done, instead the authors focused on lipidomics, for unclear reasons.

Because growth on *S. multivorum* BIGb0170 and *C. scopthalmum* JUb44 caused delayed development, altered feeding behavior, and OP50 could suppress the microsporidia growth phenotype on BIGb0170, we hypothesised that these bacteria alter nematode metabolism. To determine if growth on these two bacterial isolates changed the metabolites present in *C. elegans*, we performed untargeted metabolomics, including measuring both polar and lipid metabolites. We have now included polar metabolomic data (Supplementary Table 1) to complement our previously included lipidomic data (Supplementary Table 2). We have now modified lines 148-149 to make this rationale more clear.

Then, following up on the finding that worms on C. scopthalmum and S. multivorum have altered levels of unsaturated fatty acids, one would measure expression of a series of fatty acid desaturases, not only fat-2 (see here for the full pathway https://pmc.ncbi.nlm.nih.gov/articles/PMC2665873/). Next one would test – as the authors did – whether fat-2 mutants (or mutants of fat-7 or another component of lipid metabolism, selected based on the comparative transcriptomics or proteomics data) display reduced pathogen burden, and whether this can be rescued by adding the putative product of FAT-2, linoleic acid.

As our metabolism data showed differences in triglyceride levels, we tested whether fatty acids are necessary for microsporidia growth on *S. multivorum* BIGb0170 and *C. scopthalmum* JUb44

by supplementing bacterial diets with various fatty acids. We have now modified line 163-165 to make this rationale clear.

The results of this experiment (shown in Figure 2d) demonstrate that adding linoleic acid, even at a high level, does not rescue the phenotype. Linoleic acid has almost no effect on the very low pathogen burden of fat-2 mutant worms fed OP50. BiGb0170-fed worms do show some increase of pathogen burden, but it's unclear how that can be interpreted, given the OP50 result, and given that supplementing linoleic acid to BiGb0170 may simply rescue some of the developmental delay the worms experience on this bacteria. In fact, BigB0170-fed fat-2 mutant worms supplemented with linoleic acid have a higher pathogen load than OP-50-fed fat-2 worms supplemented with linoleic acid, strongly indicating that linoleic acid production is not the key determinant here.

We provide several lines of evidence supporting the role of linoleic acid in *N. parisii* growth. 1) We show altered levels of polyunsaturated triglyceride levels when worms are grown on BiGb0170. 2) We show that supplementation of linoleic acid increases sporulation of *N. parisii* in worms fed BiGb0170. 3) We show mutants in fat-2 cause a strong delay in spore production. 4) We show that adding linoleic acid partially reverses the disruption in spore production caused by fat-2 mutants.

We disagree with the interpretation of the reviewer that linoleic acid production is not the key determinant for rescuing spore production in fat-2 mutants. In both bacterial diets the mutants without linoleic acid do not produce any spores at the 72-hour timepoint. Upon supplementation with linoleic acid a few animals produce spores on OP50 and more animals produce spores on BigB01070. In both cases the only difference is the addition of linoleic acid which partially restores spore production. Although we do not know why we see a stronger rescue with linoleic acid in fat-2 mutant worms on OP50 compared to BigB0170, it is possible that the bacteria causes differential uptake of linoleic acid into the worms.

Similarly, the theory that production of massetolide or another natural product is responsible for the protective effects of Pseudomonas, at a minimum, needs to be followed up by testing a bacterial mutant that are deficient in the production of massetolides (see e.g., here https://pmc.ncbi.nlm.nih.gov/articles/PMC2293227/).

These are just simple examples for where very basic follow up experiments are missing or the interpretation of the data is not consistent or backed up by additional lines of evidence.

To determine if massetolides are necessary for the antimicrobial activity of Myb11, we have now tested several mutants in the biosynthetic pathway which synthesis massetolide. Mutations in any of the three genes in the pathway we tested abrogate the anti-microsporidia activity of Myb11. This data is included in the new figure panels 5c and d.

We thank the reviewers for their comments. We have provided a revised manuscript (where all changes are highlighted) to address the reviewer's concerns. Points raised by the reviewer have been incorporated into the manuscript and we have provided a point-by-point response to each of the reviewer's comments.

REVIEWERS' COMMENTS

Reviewer #1 (Remarks to the Author):

The authors have appropriately addressed my concerns.

We thank the reviewer for their comments.

Reviewer #2 (Remarks to the Author):

The authors have added new data that show that massetolid biosynthesis is required for the inhibitory activity of P. lurida MYb11. This addresses one major concern of my previous review. However, most of the hypotheses put forward to explain the effects of bacteria on N. parisii infection of C. elegans are either not well supported or not further pursued.

We thank the reviewer for their comments.

1. The role of linoleic acid. What remains unanswered in whether worms fed BIGb0170 indeed have lower 18:2 levels, and also in what other ways the FA profile may differ. The authors need to profile relative abundances of different FAs in animals grown on the different bacteria, most importantly, a comparison of 18:2 levels is needed. This is best done via total hydrolysis of all lipids (see work by Watts et al.). Unless data for changes in 18:2 abundance in the animals are presented, the author's model for the role of changes in 18:2 production remains entirely speculative.

We do not claim that 18:2 levels are lowered, just that lipid levels are altered, and sporulation is promoted by linoleic acid supplementation. We agree with the reviewer that it would be interesting to understand if BIGb0170 is altering 18:2 levels and the mechanism by which this is occurring. We have now acknowledged this limitation in the discussion in Lines 416-419: "Although our data support the role of linoleic acid as being important for *N. parisii* sporulation, the mechanism by which *S. multivorum* BIGb0170 alters lipid levels and its effects on linoleic acid levels remain to be determined."

2. Line 271 - Here the hypothesis that bacterial supernatant causes in vitro spore destruction is put forward. However, this is not further explained and I have to admit that to me it's unclear how spores could simply disappear as a result of exposure to bacterial culture supernatant.

We do not know the mechanism by which these bacterial produced compounds reduce spore levels, but this is a phenotype that we and others have previously observed (doi: 10.1126/sciadv.abf3114 and doi: 10.1038/s41598-020-68420-5). One possibility is that the chitin layer of the spore isn't present and thus the spores can not be stained with the chitin-binding dye DY96, which is how we observe the presence of the spores. We have now modified this

line to say “DY96-stained spores” to make it clear that our assay relies on the ability to stain the spores.

3. The purpose of the paragraph starting at line 295 is entirely unclear. It would be interesting to identify other anti-microsporidia compounds produced by Pseudomonas, but this is not done - the main text mentions "activity-guided purification" but this seems to end after a single Combiflash fractionation, which yielded ranged of fractions with activity.

This data is included to show that a strain of *Pseudomonas* which is not predicted to synthesize massetolides contains two separate activity peaks, suggesting that small-molecules produced by this strain have anti-microsporidia activity and that their fraction profile is distinct from that of a massetolide producing strain.

4. Similarly, whether in fact viscosin production underlies is not tested. This lack of follow-up essentially renders the preceding comparative antismash analysis of the genomes of different inhibitory and non-inhibitory species useless.

Our antismash analysis supports the presence of other compounds besides massetolides as having anti-microsporidia activity.

5. The purpose of the multi-generation experiment is unclear - the fact that protection does not wane after one generation is supportive of the idea that specific compounds produced by the bacteria inhibit or prevent infection, but seems otherwise unremarkable.

Our multi-generational experiment allows us to examine conditions closer to what infected worms would experience in the wild. This experiment allows us to show that under these more ecologically relevant conditions, that these bacteria strains can provide protection against infection for multiple generations, and that a combination of bacteria provides the strongest protection. We have now modified the results in line 372-373 to make the rationale for this experiment more clear: “To determine how continuous exposure to protective Pseudomonas isolates affects infection over successive cycles of parasite replication and transmission…”

I’m left with the impression of an assemblage of interesting observations that all show that bacteria have significant, multifaceted influence on the susceptibility of C. elegans to N. parissii infection. Most of these observations are followed up upon only superficially, and thus mechanistic hypotheses either cannot be developed or remain exceedingly speculative. The exception is the case of the massetolids, but these compounds have previously been shown to be protective of pathogen infection in C. elegans.

We disagree with the reviewer’s assessment that our manuscript is just “an assemblage of interesting observations”. We feel that our study has contributed two main advances to the understanding of how bacteria influence infectious disease caused by microsporidia. One, we show that a Bacteroidetes species alters unsaturated fatty acid metabolism in *C. elegans* and that linoleic acid is a limiting nutrient for *N. parisii* growth. Two, we show that *Pseudomonas* species make a variety of molecules, which inactivate *N. parisii* spores. We identify one of these molecules as massetolides, which have not been previously shown to provide protection against microsporidia infection. We do agree that this study setups up additional interesting lines of inquiry, which could be addressed in subsequent studies.
